# Nitazoxanide inhibits acetylated KLF5-induced bone metastasis by modulating KLF5 function in prostate cancer

**DOI:** 10.1186/s12916-023-02763-4

**Published:** 2023-02-21

**Authors:** Qingqing Huang, Mingcheng Liu, Duo Zhang, Bing-Biao Lin, Xing Fu, Zhiqian Zhang, Baotong Zhang, Jin-Tang Dong

**Affiliations:** 1 Department of Human Cell Biology and Genetics, School of Medicine, Southern University of Science and Technology, 1088 Xueyuan Blvd, Shenzhen, 518055 China; 2 Department of Urology, Kidney and Urology Center, Pelvic Floor Disorders Center, the Seventh Affiliated Hospital, Sun Yat-sen University, Shenzhen, 518000 China

**Keywords:** Prostate cancer, Bone metastasis, TGF-β, Acetylated-KLF5, Nitazoxanide, MYBL2

## Abstract

**Background:**

Castration-resistant prostate cancer often metastasizes to the bone, and such bone metastases eventually become resistant to available therapies, leading to the death of patients. Enriched in the bone, TGF-β plays a pivotal role in bone metastasis development. However, directly targeting TGF-β or its receptors has been challenging for the treatment of bone metastasis. We previously found that TGF-β induces and then depends on the acetylation of transcription factor KLF5 at K369 to regulate multiple biological processes, including the induction of EMT, cellular invasiveness, and bone metastasis. Acetylated KLF5 (Ac-KLF5) and its downstream effectors are thus potential therapeutic targets for treating TGF-β-induced bone metastasis in prostate cancer.

**Methods:**

A spheroid invasion assay was applied to prostate cancer cells expressing KLF5^K369Q^, which mimics Ac-KLF5, to screen 1987 FDA-approved drugs for invasion suppression. Luciferase- and KLF5^K369Q^-expressing cells were injected into nude mice via the tail artery to model bone metastasis. Bioluminescence imaging, micro-CT), and histological analyses were applied to monitor and evaluate bone metastases. RNA-sequencing, bioinformatic, and biochemical analyses were used to understand nitazoxanide (NTZ)-regulated genes, signaling pathways, and the underlying mechanisms. The binding of NTZ to KLF5 proteins was evaluated using fluorescence titration, high-performance liquid chromatography (HPLC), and circular dichroism (CD) analysis.

**Results:**

NTZ, an anthelmintic agent, was identified as a potent invasion inhibitor in the screening and validation assays. In KLF5^K369Q^-induced bone metastasis, NTZ exerted a potent inhibitory effect in preventive and therapeutic modes. NTZ also inhibited osteoclast differentiation, a cellular process responsible for bone metastasis induced by KLF5^K369Q^. NTZ attenuated the function of KLF5^K369Q^ in 127 genes’ upregulation and 114 genes’ downregulation. Some genes’ expression changes were significantly associated with worse overall survival in patients with prostate cancer. One such change was the upregulation of *MYBL2*, which functionally promotes bone metastasis in prostate cancer. Additional analyses demonstrated that NTZ bound to the KLF5 protein, KLF5^K369Q^ bound to the promoter of *MYBL2* to activate its transcription, and NTZ attenuated the binding of KLF5^K369Q^ to the *MYBL2* promoter.

**Conclusions:**

NTZ is a potential therapeutic agent for bone metastasis induced by the TGF-β/Ac-KLF5 signaling axis in prostate cancer and likely other cancers.

**Supplementary Information:**

The online version contains supplementary material available at 10.1186/s12916-023-02763-4.

## Background

Prostate cancer (PCa) is one of the leading causes of cancer-related mortality in men [1]. In the initial stage of PCa, localized tumors can be successfully treated by surgery or radiation therapy, but about 20–30% of patients will relapse [2]. Although relapsed patients are usually initially sensitive to androgen deprivation therapy, they will eventually develop castration-resistant prostate cancer (CRPC) [3]. Most CRPCs metastasize, and about 90% of them metastasize to the bones [4, 5]. Similar to bone metastases of other malignancies, metastases of CRPC in the bone initially respond to targeted therapies such as taxanes. Still, virtually all of them develop therapy resistance and become incurable. For drug-resistant bone metastases, available treatments such as denosumab, zoledronic acid, and bisphosphonates can only alleviate symptoms or morbidity and prevent or delay skeletal-related events (SRE). Such treatments do not significantly improve the overall survival of patients [6, 7]. Therefore, developing novel and effective therapies against drug-resistant bone metastasis is urgently needed for prostate cancer and other types of malignancies that develop bone metastases, including breast cancer, lung cancer, and multiple myeloma.

The bone environment is rich in TGF-β (transforming growth factor-β), a cytokine regulating various physiological and pathological processes. TGF-β plays a pivotal role in the development of bone metastasis, even though it suppresses cell proliferation and tumor growth in the early stages of tumorigenesis [8–11]. Although the TGF-β signaling is an attractive target for treating bone metastasis, directly targeting TGF-β and its receptors have proven challenging for different reasons [12–14]. For example, TGF-β has critical functions in tissue homeostasis [14], and TGF-β-targeting compounds are often not selective and thus could cause toxicities to cardiac tissues and skin [15, 16].

Our previous studies demonstrated that, in epithelial cells, TGF-β induces the acetylation of the transcription factor KLF5 (Krüppel-like factor 5) at lysine 369 (K369) [17, 18]. Subsequently, TGF-β and acetylated KLF5 (Ac-KLF5) form a signaling axis to regulate gene transcription and various cellular processes such as cell proliferation, cell motility and invasion, and epithelial-mesenchymal transition (EMT) [17–21]. More recently, we found that the TGF-β/Ac-KLF5 axis also induces bone metastasis and drug resistance in prostate cancer [22, 23]. Importantly, prostate cancer metastases from the TGF-β-rich bone environment indeed express higher levels of Ac-KLF5 than those from visceral tissues [23]. The facts that TGF-β and Ac-KLF5 form an axis and that Ac-KLF5 is essential for TGF-β to induce bone metastasis suggest that Ac-KLF5 and its downstream effectors are alternative therapeutic targets for the treatment of TGF-β-induced bone metastasis in PCa.

In this study, we adopted an in vitro spheroid invasion screening assay to prostate cancer cells expressing KLF5^K369Q^, a mutant mimicking Ac-KLF5 [22, 23]. Using the system, we screened 1987 FDA-approved drugs to identify drugs that inhibit cell invasion and bone metastasis. Here, we describe the screening outcomes and present data that an anthelmintic drug-nitazoxanide (NTZ), as a potent inhibitor of bone metastasis in a mouse model. We also present cellular and molecular data that support NTZ’s inhibitory effect on bone metastasis and provide mechanisms for how NTZ acts to suppress bone metastasis.

## Methods

### Drugs

FDA-approved drug library mini (Cat #: HY-L022M), which contained 1987 drugs in 96-well plates and dissolved in 10 μL dimethyl sulfoxide (DMSO) at 10 mM, was purchased from MedChemExpress (Shanghai, China). Additional nitazoxanide (Cat #: HY-B0217) was purchased from MedChemExpress and dissolved in DMSO at 100 mM concentration.

### Cell lines

We have previously established stable PC-3 and DU 145 human PCa cell lines that express the wild-type KLF5, the Ac-KLF5-mimicking mutant KLF5^K369Q^ (KQ), the acetylation-deficient mutant KLF5^K369R^ (KR), and the pLHCX vector control [22]. PC-3 cells expressing different forms of KLF5, including PC-3-KLF5, PC-3-KQ, PC-3-KR, and PC-3-pLHCX, were maintained in RPMI-1640 medium supplemented with 10% fetal bovine serum (FBS, Biological Industries, HAMEK, Israel) and 1% penicillin/streptomycin (100 U/mL, Biological Industries). DU 145 cells expressing different forms of KLF5 were cultured in the minimum Eagle’s medium (MEM) (Corning, New York, USA) with 10% FBS.

PC-3-KLF5, PC-3-KQ, and PC-3-KR cells were infected with lentiviruses expressing the luciferase (HBLV-LUC-PURO, Hanbio, Shanghai, China). Infected cells were selected for stable cell populations in a medium containing puromycin (2 μg/mL).

RAW264.7 macrophage cell line was obtained from the American Type Cell Culture (ATCC, Manassas, VA, USA) and cultured in Dulbecco's modified eagle medium (DMEM) (Biological Industries) supplemented with 10% FBS. All cells were cultured at 37°C in a humidified atmosphere with 5% CO_2_.

### Migration assay

PC-3 cells expressing different forms of KLF5 were suspended in a serum-free medium and seeded onto the upper chamber of a trans-well device (Cat #: 353097, Corning) at 5 × 10^4^ cells per well. Seven hundred and fifty μL complete medium containing 15% FBS was added to the lower chamber. Forty-eight hours later, cells on the upper side of the membrane were scraped using a cotton swap, while cells on the lower side were stained with purple crystal (Cat #: C0121, Beyotime Biotechnology, Shanghai, China) and photographed using a stereoscope (Mshot, Guangzhou, China). The membrane was then placed into 500 μL of 33% acetic acid in the lower chamber to dissolve cells. Optical densities of dissolved cells were measured using a microplate reader (BioTek, Winooski, Vermont, USA) at 570 nm.

### Invasion assay and screening for invasion-inhibiting drugs

**Fig. 1:**
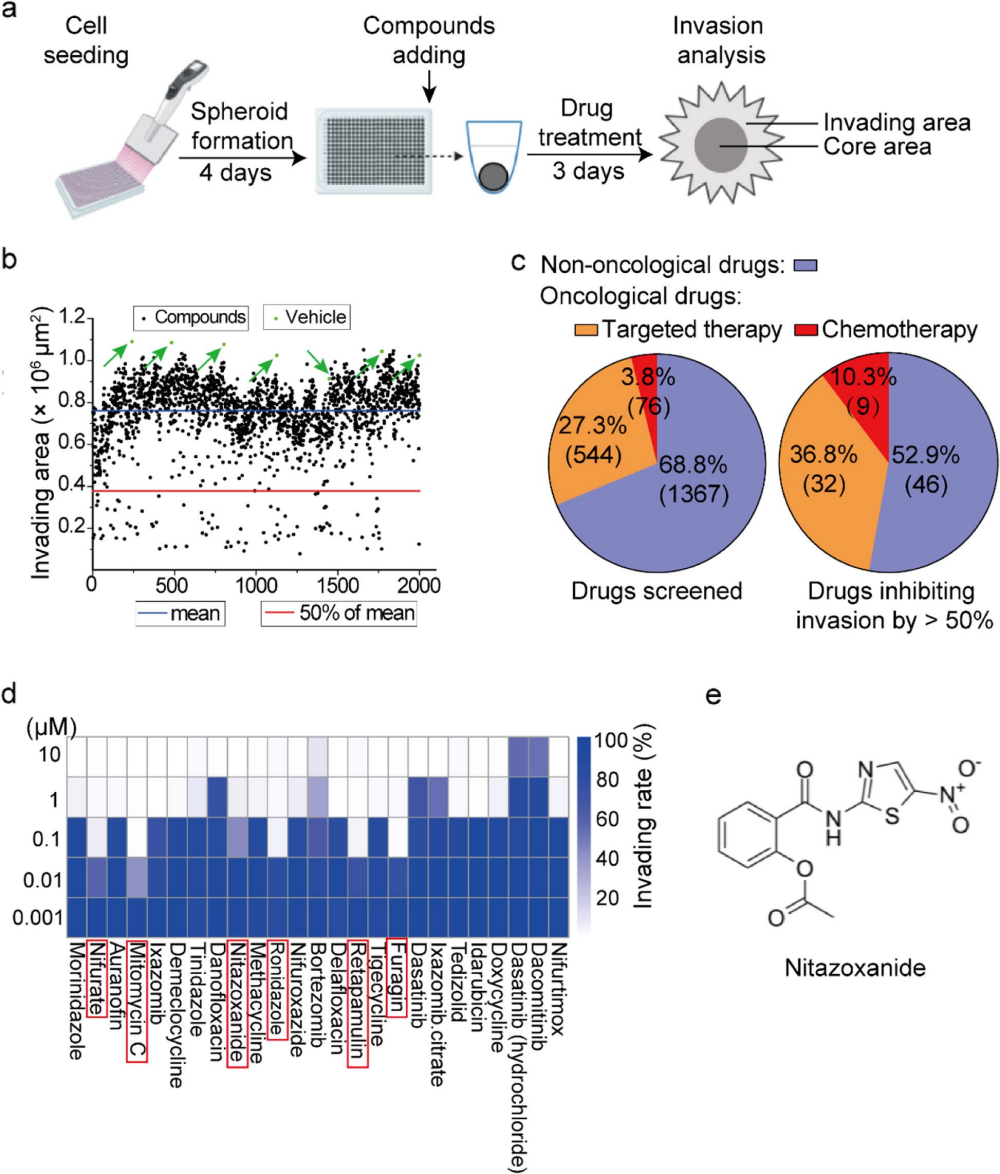
Screening of FDA-approved drugs for those that inhibit cellular invasion induced by acetylated KLF5. **a** Schematic of the screening workflow. **b** Inhibitory effects of 1987 FDA-approved drugs, along with vehicle control (green dots pointed by green arrows), on the invasion of PC-3-KQ cells, as analyzed using the 3D spheroid invasion assay. Each dot represents a drug or control, and the dots are aligned along the *X*-axis. *Y*-axis indicates the average invasion area between two well for each drug. The blue horizontal line indicates the mean invasion area of all drugs and controls, while the red horizontal line indicates 50% of the mean invasion area. **c** Proportions of the 1987 drugs that are approved as non-oncological drugs (purple color), oncological targeted therapeutic drugs (orange color), and oncological chemotherapeutic drugs (red color). **d** Identification of the most effective 25 drugs by using the 3D spheroid invasion assay with multiple concentrations (μM). The heatmap shows the invasion rates (%) of each drug’s multiple concentrations when compared to the vehicle control, as indicated by blue grids with varying intensities. Names of the 25 drugs are shown at the bottom, with 6 of the most effective ones marked by red boxes. **e** The chemical structure of nitazoxanide

The invasion assay was performed as previously described [24]. Briefly, PC-3-KQ cells at 80-90% confluency were plated onto 384-well round bottom plates with ultra-low attachment (Cat #: 4516, Corning) at 500 cells per well in 80 μL medium. The plates were then centrifuged at 320 g for 4 min to cluster cells in the bottom of wells and incubated for 3 days. On the fourth day, 40 μL medium was removed from each well, 40 μL of the matrigel-medium mixture was added to each well, and cells were cultured for another 3 days. The matrigel (Cat #: 354234, Corning) was from Corning, and the medium contained 1% FBS. At the end of the incubation, images were taken using a phase contrast microscope (Eclipse Ti2, Nikon, Tokyo, Japan) with 10× object lenses, and the total area of cells and the core sphere area of cells (Fig. 1a) were measured using the Image J software. The invading area was calculated using the following formula: invasion area = total area of cells – core sphere area [25].

For drug screening, drugs were added to the matrigel-medium mixture when replacing the culture medium on day 4. For the initial round of screening, a final concentration of 10 μM was used for all drugs. Multiple drug concentrations were used for the second round of screening, including 0, 0.001, 0.01, 0.1, 1, and 10 μM. Duplicate wells were repeated for each concentration.

### Cell Counting Kit-8 (CCK-8) assay

According to the manufacturer's instructions, cell viability was measured using a kit from Beyotime Biotechnology. Briefly, cells were seeded onto 96-well plates at 5 × 10^3^ cells per well, incubated overnight, and then treated for 48 h with drugs at 0, 0.02, 0.05, 0.14, 0.41, 1.23, 3.7, 11.1, 33.3, and 100 μM. Four drugs were analyzed: furagin, nifuratel, nitazoxanide, and retapamulin. At the end of drug treatment, the medium was removed, the CCK-8 solution was added into each well at 100 μL/well and incubated for 1.5 h at 37°C, and the optical density (OD) was measured at 450 nm using a microplate reader (BioTek).

We also tested a wider range of NTZ concentrations in PC-3-KQ and DU 145-KQ cells using the CCK-8 assay by seeding 2 × 10^3^ cells and 2.5 × 10^3^ cells onto each well of 96-well plates. PC-3-KQ cells were treated with NTZ at 0, 0.01, 0.1, 1,25, 2.5, and 5 μM, while DU 145-KQ cells were treated with NTZ at 0, 0.01, 0.1, 5, 10, and 20 μM for 0, 24, 48, and 72 h. The number of viable cells was determined using the CCK-8 solution as described above.

### Clone formation assay

PC-3-KQ and DU 145-KQ cells were seeded onto 6-well plates at 1 × 10^3^ cells/well. After incubation for 24 h, PC-3-KQ cells were treated with NTZ at 0, 1.25, 2.5, and 5 μM, while DU 145-KQ cells were treated with NTZ at 0, 5, 10, and 20 μM for 8 days. The drug-containing medium was replaced every 2 days. On the 8^th^ day, cells were rinsed with cold PBS, fixed with 4% paraformaldehyde for 10 min, stained with purple crystal for 10 min, and photographed, and the images were analyzed using the Image J software.

### Bone metastasis assay

Male Balb/c nude mice at 3–4 weeks old were purchased from Charles River (Beijing, China). Upon arrival at the Animal Center of the Southern University of Science and Technology (SUSTech), mice were quarantined for one week before use. All mice were maintained and handled following the National Institutes of Health guide for the Care and Use of Laboratory Animals.

To measure if NTZ inhibits Ac-KLF5-induced bone metastasis, we used both prevention and therapy modes. For the prevention model, following a recently published method [26], mice were anesthetized, the tail artery was wiped with alcohol to expand the blood vessel, and 1×10^6^ cells cancer cells (PC-3-KQ-Luc or PC-3-KR-Luc cells) in 100 μL PBS were injected into the tail artery in a short time (< 3 s) [26]. Then the mice were randomly divided into vehicle and NTZ groups (six mice in each group) and received treatment at day 0. The tumor growth and body weight were determined every week. After 5 weeks of treatment, all mice were sacrificed and bone tissues and major organs were resected for hematoxylin and eosin (H&E) staining. The selection of 5 weeks for investigation is based on the pilot experiment, in which clear bone metastases formed at 5 weeks after injection but no tumors were detected in other organs, including the brain, liver, lung, spleen, heart, prostate, seminal vesicle, small intestine, and testes (data not shown).

For the therapy model, 1×10^6^ PC-3-KQ-Luc cells in 100 μL PBS were injected into the caudal artery of each mouse as described above. Every 7 days, the mice were performed with bioluminescence (BL) imaging and randomly divided into the vehicle and NTZ groups (*n* = 6 each group) and received treatment at day 7. The tumor growth and body weight were measured each week. At 35 days after the final treatment, the bone tissues of each group were stripped out for high-resolution microcomputed tomography (micro-CT), H&E staining, and immunohistochemical staining.

Tumor growth in the bone was determined once a week by in vivo BL imaging using the Living Image Software 4.4 (IVIS Spectrum, PerkinElmer Health Sciences, Massachusetts, USA). Ten minutes after intraperitoneal injection of D-luciferin sodium salt (15 mg/mL, 10 μL/g BW) (GM-040611, Genomeditech, Shanghai, China), bioluminescence images were acquired with the following conditions: open emission filter, exposure time = 60 s, binning = medium: 8, field of view = 12.9 ×12.9 cm, and f/stop = 1. Images were then analyzed using the Living Image 4.4 software (PerkinElmer). At the end of treatment, bioluminescence intensities were measured by photon flux of the region of interest (ROI) and compared between the vehicle and NTZ treatment groups.

For drug treatment, 1% sodium carboxymethyl cellulose (CMC) (Cat #: CC0113, Leagene Biotechnology, Beijing, China) was used as vehicle control, and NTZ was solved in CMC. NTZ was delivered into mice via intragastric administration (100 mg/kg BW) every day.

### Micro-CT scanning and analysis

After the sacrifice of mice, hind limbs were removed and fixed in 4% paraformaldehyde for 48 h. Limbs were then scanned using a micro-CT scanner (Skyscan1276, Bruker, Kontich, Belgium). For micro-CT imaging, the scan parameters were: source voltage, 60 kV; source current, 100 μA; AI 0.5 mm filter; pixel size, 10 μm; rotation step, 0.4 degrees. The images were then processed and constructed using the NRecon software (Version 1.7.1.6, Bruker) and converted using Dataviewer (Version 1.5.4, Bruker). The region of interest (ROI), set at 0.5 mm from the femur growth plate, was analyzed using the CT-Analyser (CTAn) program. Parameters included bone volume/total volume (BV/TV), trabecular number (Tb.N), trabecular separation (Tb.Sp), and trabecular thickness (Tb.Th). Finally, the 3D images were performed in CTvox software (Version 2.0, Bruker).

### Histological analysis of bone tissues

Femurs were decalcified in ethylenediaminetetraacetic (EDTA) decalcification solution (Cat #: R20403-5L, OKA, Beijing, China) for 14–21 days. After decalcification, bone tissues were dehydrated in gradient ethanol from 70 to 95%, soaked in n-butanol for 6 h, and then embedded in paraffin. Tissue blocks were cut into 4 μm sections. After baking in an oven at 63°C for 1.5 h, tissue slides were deparaffinized with xylene, hydrated with gradient ethanol, and then stained with H&E (Cat #: BA4025, BaSO, Zhuhai, China). Slides were then mounted for analysis with neutral resins (Cat #: 25608-33-7, Sigma-Aldrich, MO, USA).

### Immunohistochemical staining

Immunohistochemical staining was conducted following established procedures. Briefly, 4-μm sections of bone tissues were deparaffinized in xylene and rehydrated in gradient ethanol. The endogenous antigen was retrieved by incubating slides in 10 mM sodium citrate buffer (Cat #: C1010, Solarbio, Beijing, China) overnight in an oven at 65°C. After antigen retrieving, tissue sections were incubated with 3% hydrogen peroxide at room temperature for 10 min to block the endogenous catalase activity. They were then incubated in a blocking solution (0.1% albumin bovine V mixed with 10% goat serum) for 40 min, and a solution containing a primary antibody (MMP9, 1:1000, Cat #: ab76003, Abcam, Cambridge, UK; MYBL2, 1:100, Cat #: ab76009, Abcam) for 1.5 h. After rinsing with PBS for 3 times, slides were incubated with the secondary antibody solution for 15 min and then with the DAB solution using the MaxVision II HRP kit (Cat #: KIT-5920, MXB Biotechnologies, Fuzhou, China). Nuclei were counterstained with hematoxylin for 2 min. Slides were then dehydrated with gradient ethanol (75–100%) and xylene and mounted with coverslips using the quick-hardening mounting medium. All slides were scanned using an Aperio VERSA 8 Scanner System (Leica, Wetzlar, Germany) and analyzed using the Image J software (Image J 1.48v, NIH, Bethesda, MD, USA).

### Osteoclastogenesis assays in cultured cells and tissues

Three different culture systems were used for the in vitro osteoclast differentiation assay. In the first one, RAW264.7 cells were seeded onto 96-well plates at 1 × 10^3^ cells/well and cultured overnight. Cells were then stimulated with recombinant mouse RANKL (Cat #: CR06, Novoprotein, Shanghai, China) at 50 ng/mL in the presence or absence of various concentrations of NTZ (0, 6.25, 12.5, 25 μM). The medium was replaced every 2 days until osteoclasts were observed in the control group. Cells were then fixed in 4% paraformaldehyde for 10 min, rinsed with prewarmed deionized water, and stained with tartrate-resistant acid phosphatase (TRAP) using a kit from Sigma-Aldrich (Cat #: A387) to visualize osteoclasts (≥ 3 nuclei/cell). The effect of NTZ on osteoclast differentiation was determined by counting the number of multinucleated cells using the Image J software (Image J 1.48v).

In the second in vitro assay, we used conditioned medium (CM) from NTZ-treated Ac-KLF5-expressing cells. PC-3-KQ cells were seeded in 6-well plates at 1 × 10^5^ cells per well and cultured overnight. Cells were treated with NTZ at 0, 1.25, 2.5, and 5 μM for 36 h in complete media (RPMI1640 with 10% FBS and 1% penicillin-streptomycin). The medium was removed, and cells were washed twice with the RPMI1640 medium containing 0.5% FBS and cultured in the same medium for another 12 h. Subsequently, the conditioned media (CM) was collected from each group, centrifugated, filtered, aliquoted, and stored at -80°C for further use or used immediately in the co-cultured experiments. CM from each group was mixed with the DMEM medium (10 % FBS) at a ratio of 1:3, and RANKL was added to a lower concentration (10 ng/mL). RAW264.7 cells in 96-well plates with 24-h culture were incubated with the CM-containing medium for 7 days, with the medium replaced every 2 days. The TRAP staining assay was performed to determine the number of differentiated osteoclasts according to the manufacturer's instructions.

The third in vitro assay involved direct co-culture between RAW264.7 cells and cancer cells. RAW264.7 cells were seeded in 96-well plates at 700 cells per well and allowed to adhere for 12 h. PC-3-KQ cells were added to each well at 300 cells/well, and the culture continued for 12 h. NTZ was added to treat cells for 48 h at various final concentrations (0, 1.25, 2.5, 5 μM). The RAW264.7 medium was mixed with the PC-3 medium at 7:3, and the mixture was used to replace the culture medium every 2 days. After 7 days, the osteoclast differentiation was determined as described above.

The procedure for TRAP staining in tissues was described in our previous study [23]. Briefly, deparaffinized and hydrated bone sections were incubated in 0.2 M acetic acid for 20 min and then in 0.2 M acetic acid containing the fast red TR salt (1.1 mg/mL) and naphthol AS-MX phosphate (0.5 mg/mL) for about 45 min at 37°C. When TRAP-positive cells turn red, as monitored using microscopy, slides were counterstained with hematoxylin and mounted with coverslips using glycerin. Slides were then scanned and analyzed as described above.

### RNA-sequencing and bioinformatic analyses

PC-3-KQ and PC-3-KR cells were seeded onto 6-well plates at 1 × 10^5^ cells/well and cultured for 24 h. The culture medium was then replaced with the one containing 0 or 5 μM NTZ, and the treatment was for 48 h. Cells were then rinsed once with pre-chilled PBS and collected in the TRIzol reagent (Cat #: 15596018, Invitrogen, Carlsbad, California, USA). Extraction of RNA, construction of libraries using the SE100 protocol, and sequencing with a DNBSEQ platform with pair-end 100 bp sequencing length were performed by the Beijing Genomics Institute (Wuhan, China). Raw data processing was completed as previously described [14]. All RNA-seq data were processed by FASTQC for quality assessment and aligned to the human genome using Bowtie2 (V2.2.5) [27].

The edgeR algorithm was performed to identify differentially expressed genes between KLF5^K369Q^- and KLF5^K369R^-expressing cells and NTZ treatment and control in KLF5^K369Q^-expressing cells, with a |fold change| ≥ 1.5 and a false discovery rate < 0.05. Our sequencing data have been deposited to the Gene Expression Omnibus database with the accession number GSE216126.

### Survival analysis

For the genes whose expression was modulated specifically by KLF5^K369Q^ and such modulation was attenuated by NTZ, the Kaplan–Meier survival analysis was performed to evaluate whether a gene’s expression changes in human prostate cancer is related to patient survival. We analyzed data from the Stand Up To Cancer/Prostate Cancer Foundation (SU2C/PCF) East Coast Dream Team (ECDT) (https://www.cbioportal.org/ ) using the Survival R package. Patients were stratified into two groups based on a gene’s median expression level in each dataset. Log-rank test was used to calculate p values. Univariate hazard ratios were also calculated.

Genes significantly associated with survival were compared for expression levels among normal prostate tissues, prostate cancers, and bone metastasis using the SU2C/PCF dataset [28] and the GSE21034 dataset [29]. Wilcoxon rank-sum test was used to determine p values for comparisons between two groups, while the Kruskal-Wallis test was used for multi-group comparisons. Two-sided statistical testing was performed, and a *p* value < 0.05 was considered statistically significant. All analyses were run in R4.1.3 (https://www.R-project.org/).

### Quantitative real-time PCR (qRT-PCR)

Total RNA was extracted from cells using the Eastep Super total RNA extraction kit (Cat #: LS1040, Promega, Shanghai, China) and quantified using the NanoDrop One (Thermo Fisher Scientific, Madison, USA). Single-strand cDNA was synthesized from 1 μg of total RNA using the HiScript III All-in-one RT SuperMix kit (Cat #: R333-01, Vazyme, Nanjing, China). For qRT-PCR, the cDNA template was mixed with the SYBR Green reagent in enzyme-free water, and the Qtower3 touch system (Analytik Jena, Jena, Germany) was used for PCR with the following program: initial denaturation at 95°C for 5 min, 40 cycles of 95°C for 30 s, 60°C for 30 s, and 72 °C for 30 s, and a final melting for 15 s. GAPDH was used as an internal control. Primer sequences were as follows: 5′-CTTGAGCGAGTCCAAAGACTG-3′ (MYBL2 forward), 5′-AGTTGGTCAGAAGACTTCCCT-3′ (MYBL2 reverse), 5′-CAGCATTTCATCGAGGTAGAGAC-3′ (TIMM8A forward), 5′-AGCCCGACTGTCCAACTTTG-3′ (TIMM8A reverse), 5′-TGAAGGTGACAGAGCCTCTGGAT-3′ (E-cadherin forward), 5′-TGGGTGAATTCGGGCTTGTT-3′ (E-cadherin reverse), 5′-TGAAGGTGACAGAGCCTCTGGAT-3′ (Vimentin forward), 5′-CTTGTAGGAGTGTCGGTTGTTAAG-3′ (Vimentin reverse), 5′-GACAATGCCCCTCAAGTGTT-3′ (N-cadherin forward), 5′-CCATTAAGCCGAGTGATGGT-3′ (N-cadherin reverse), 5′-CTTCCAGCAGCCCTACGAC-3′ (Snail forward), 5′-CGGTGGGGTTGAGGATCT-3′ (Snail reverse), 5′-TGTTTGCAAGATCTGCGGC-3′ (Slug forward), 5′-TGCAGTCAGGGCAAGAAAAA-3′ (Slug reverse), 5′-CCATAAAGGGCAACCAAGAG-3′ (Fibronectin forward), 5′-ACCTCGGTGTTGTAAGGTGG-3′ (Fibronectin reverse), 5′-TGCAGTCAGGGCAAGAAAAA-3′ (Slug reverse), 5′-TGTACCGCTATGGTTACACTCG -3′ (MMP9 forward), 5′-GGCAGGGACAGTTGCTTCT-3′ (MMP9 reverse), 5′-GGTGGTCTCCTCTGACTTCAACA-3′ (GAPDH forward), and 5′-GTTGCTGTAGCCAAATTCGTTGT-3′ (GAPDH reverse).

### Western blotting

Cells in 6-well plates were washed with cold PBS and collected in a lysis buffer containing phosphatase and protease inhibitors (Cat #: P1045, Beyotime) to extract proteins. After centrifugation for 10 min at 4°C in a refrigerated centrifuge, the supernatant was collected and mixed with the 4× loading buffer, boiled in a metal heat bath at 100°C for 10 min, and then subjected to 10% SDS-PAGE. Proteins were transferred onto an activated PVDF membrane (0.45 μm pore-size, Cat #: IPVH00010, Millipore, Carrigtwohill, Ireland). The membrane was blocked with 5% skimmed milk for 40 min at room temperature, incubated with a primary antibody at 4°C overnight, washed with PBS 3 times (10 min each), and incubated with HRP-conjugated secondary antibody against the rabbit IgG (1:5000, Cat #:7074S, Cell Signaling Technology, Danvers, MA, USA) for 1 h at room temperature. Signals were visualized using the ECL substrate reagent (Cat #: K-12043-D20, Advansta, California, USA) in an automatic chemiluminescence analyzer (ChampChemi 610 plus, Beijing, China). The GAPDH antibody was purchased from Cell Signaling Technology (1:2000, Cat #: 5174S), the KLF5 antibody was from Proteintech (1:1000, Cat #: 21017-1-AP, Chicago, USA), the MYBL2 antibody was from Abcam (1:2000, Cat #: ab76009, Cambridge, MA, USA) and the MMP9 antibody was from Abcam (1:2000, Cat#: ab 76003).

### RNA interference

Small interfering RNAs (siRNAs) for *KLF5* and negative control were synthesized by Ribobio (Guangzhou, China) and used to transfect cells at 50 nM using the Lipofectamine RNAiMax reagent (Cat #: 13778150, Invitrogen) according to the manufacturer’s instruction. After 48 h, cells were collected for analyses, including the knockdown efficiency test by western blotting. The sequence of *KLF5* siRNA was 5′-AAGCUCACCUGAGGACUCA-3′ [30].

### Chromatin immunoprecipitation (ChIP) assay

ChIP assay was performed using the SimpleChIP Enzymatic Chromatin IP kit (Cat #: 9003s, Cell Signaling Technology) according to the manufacturer's instructions. Briefly, cells were treated with 1% formaldehyde for 10 min for cross-linking, quenched with glycine for 5 min at room temperature, collected, and digested with micrococcal nuclease for 20 min at 37°C. After stopping the reaction by adding EDTA, DNA was fragmented by sonication, and the extracts were incubated with KLF5 antibody (Cat #: 21017-1-AP, Proteintech) or IgG (Cat #: 2729P, Cell Signaling Technology). Eluted DNA fragments were detected by regular PCR and quantitative real-time PCR using the following primers: 5′-CCTTCCTCGGTCTTCGCTAT-3′ (MYBL2 #1 forward), 5′-GCACTTTTCTATCTCCCGCCA-3′ (MYBL2#1 reverse), 5′-CCTGGAGATACTGGTGTGCAT-3′ (MYBL2 #2 forward), and 5′-GGCCAAAAGAAACGGCCTCT-3′ (MYBL2 #2 reverse).

### Expression and purification of KLF5 protein

Wild-type *KLF5* and the *KLF5*^*K369Q*^ and *KLF5*^*K369R*^ mutants were cloned into the pET15b expression vector (Cat #: ZK146, Zoman Biotechnology, Beijing, China), in which a histidine tag was added to the N terminus of KLF5 protein upon expression. The plasmids were expressed in the BL21 DE3 strain of *E. coli* (Cat #: EC1003, Weidi Biotechnology, Shanghai, China). Bacteria were collected by centrifugation, sonicated in 10 mM imidazole in PBS (10 mM, pH 7.4), and incubated with the His60 Ni Superflow resin (Cat #: L00666, GenScript, Nanjing, China). His-KLF5 resin complexes were washed and eluted with 300 mM imidazole in PBS (10 mM, pH 7.4). KLF5 protein was further purified by gel filtration on a Superdex 200 Increase 10/300 column (Cat #: 10243519, GE, MA, USA) using a PBS (10 mM, pH 7.4) for use in binding assays.

### Fluorescence titration of KLF5 proteins in the presence of NTZ

Wild-type or mutant KLF5 protein at 1 μM in 2 mL PBS was measured for fluorescence spectrum using a fluorescence spectrometer (HORIBA, Kyoto, Japan), and the fluorescence spectra were obtained for wavelengths of 300–500 nm. NTZ with various concentration ranges and various increments were used in the analysis, including 0.1–1 μM with an increment of 0.1 μM, 1–5 μM with an increment of 0.5 μM, 5–10 μM with an increment of 1 μM, and 10–30 μM with an increment of 5 μM. The incubation with NTZ was for 5 s at room temperature. Data were processed and fitted to obtain the binding affinities using the *Origin* software (OriginLab, Northampton, MA, USA), and Kd values were determined by the responses at 290 nm wavelength.

### Detection of KLF5-bound NTZ using high-performance liquid chromatography (HPLC)

Purified KLF5 protein or its mutant at 0.5 mg/mL in 0.5 mL was incubated with His60 Ni Superflow resin for 30 min at room temperature. After washing twice, the protein-beads complexes were diluted to 1 mL in PBS. His60 Ni Superflow resin without protein was used as a negative control (i.e., blank). Ten microliters of 1 mM NTZ was added to the protein-beads complexes and incubated for 1 h at room temperature. The reactions were washed with PBS and then incubated with acetonitrile to denature protein and release captured NTZ. NTZ in the supernatant was then detected by HPLC analysis using the Agilent 1260 Infinity II instrument (California, USA) and the C18 column (25 cm × 4.6 mm, 5 μm) with a flow rate of 0.5 mL/min for 20 min at the wavelength of 298 nm.

### Circular dichroism (CD) analysis

Wild-type or mutant KLF5 protein in 2 mL PBS at 0.01 mg/mL was analyzed using a CD spectropolarimeter (Chirascan, Applied Photophysics, Surrey, UK) at 200–260 nm. The spectra were recorded in a 1 cm path-length cuvette. An average of three repeated scans were performed on each spectrum. NTZ was added to the protein solution at a final concentration of 1 μM and incubated for 2 h at 4°C before CD detection.

### Statistical analysis

Quantitative data were presented as means ± SD, and all experiments were repeated at least three. Student’s *t*-test or one-way ANOVA was used for statistical analysis. All statistical analyses were conducted using GraphPad Prism 6 software [31]. A *p* value < 0.05 was considered statistically significant.

## Results

### Identification of NTZ as a potent novel inhibitor of Ac-KLF5-induced invasion of prostate cancer cells

We confirmed that, among the commonly used prostate cancer cell lines (LNCaP, C4-2B, PC-3, and DU 145), PC-3 cells expressed the highest level of KLF5 (Additional file 1: Fig. S1a). We thus chose the previously established KLF5-null PC-3 cell lines expressing the wild-type KLF5, the Ac-KLF5-mimicking KLF5^K369Q^ mutant, and the acetylation-deficient KLF5^K369R^ mutant [23] for drug screening and metastasis modeling. Expression levels of KLF5 in these cell lines are shown in Fig. S1b. We also validated that KLF5^K369Q^-expressing cells were more migrative (Additional file 1: Fig. S1c, S1d) and invasive in the transwell assay (Additional file 1: Fig. S1e, S1f) than KLF5- and KLF5^K369R^-expressing cells. As expected, KLF5^K369Q^-expressing cells also expressed higher levels of mesenchymal markers, including vimentin, N-cadherin, snail, slug, and fibronectin (Additional file 1: Fig. S1g, S1h).

The 3D spheroid invasion assay was used to test 1987 FDA-approved drugs for their ability to inhibit cell invasion (Fig. 1a). At 10 μM, 87 of the 1987 drugs inhibited cell invasion by at least 50%, including 9 chemotherapy agents, 32 targeted cancer drugs, and 46 non-oncological drugs (Fig. 1b, c; Additional file 2: Table S1).

For the 87 drugs, we repeated the invasion screening using a series of concentrations for each drug, including 0, 0.001, 0.01, 0.1, 1, and 10 μM. These drugs’ effects on cell invasion are listed in Table S1. The invasion assay with multiple drug concentrations was repeated for the top 25 of the 87 drugs that showed a dose-dependent invasion inhibition, and their invasion inhibition capabilities were confirmed again (Additional file 2: Table S2). These 25 drugs are listed in Table 1 in the order of their invasion inhibitory capabilities.

**Table 1:**
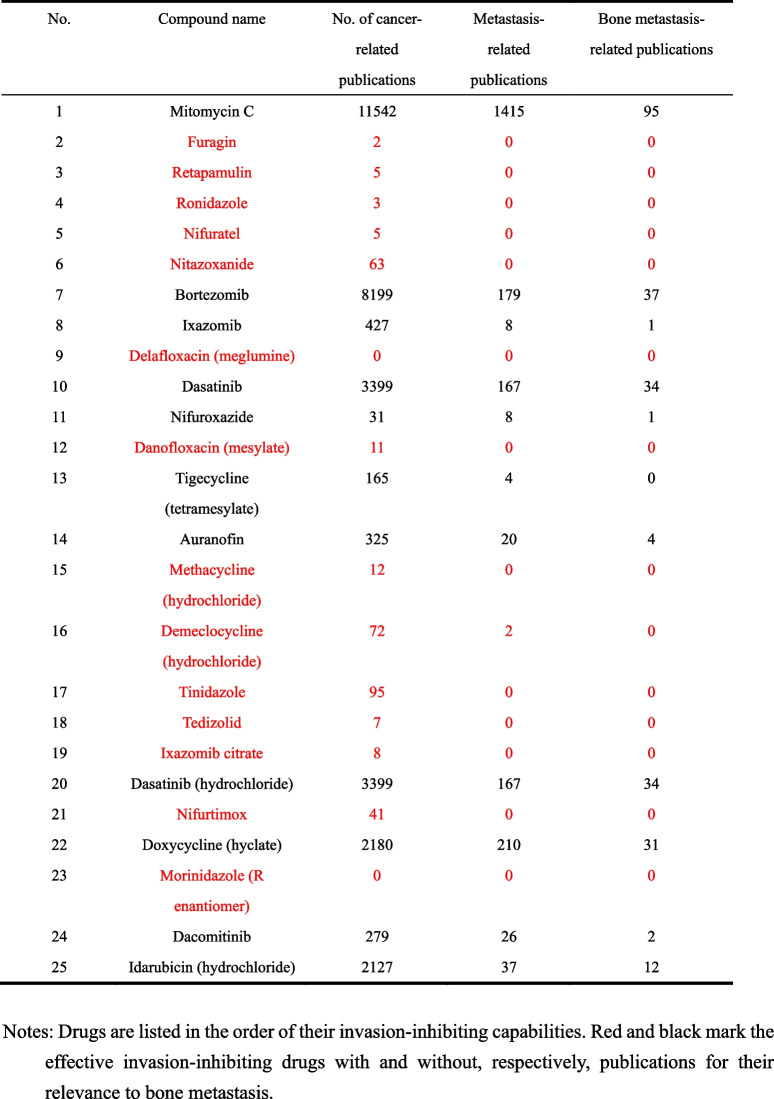
List of the most effective 25 drugs after the second round of invasion screening

For these 25 drugs, we searched the PubMed database for publications that contained a drug’s name and “cancer,” “metastasis,” or “bone metastasis” to evaluate whether these drugs have been implicated in cancer and metastasis. Of the 25 drugs, 11 had been well implicated in cancer or metastasis in the literature, while 14 had limited or no cancer-related publications (Table 1).

Of the 25 drugs, 6 inhibited cell invasion by more than 50% at 0.1 μM, including nifuratel, mitomycin C, nitazoxanide, ronidazole, retapamulin, and furagin (Fig. 1d, Table 1). Of the 6 drugs, mitomycin C and ronidazole were excluded for further study, as the former has been widely used to treat cancer and cancer metastasis, while the latter is an antiprotozoal agent used in veterinary medicine but not approved for human use. For the remaining 4 drugs, we determined the killing specificity and IC_50_ values in PC-3 and DU 145 prostate cancer cells expressing KLF5, KLF5^K369Q^, and KLF5^K369Q^ using the CCK-8 assay. Only nitazoxanide (NTZ) of the 4 drugs showed a relatively smaller IC_50_ on KLF5^K369Q^-expressing cells than KLF5- and KLF5^K369R^-expressing cells (Table 2). NTZ was also more potent in invasion inhibition in expressing KLF5^K369Q^ than KLF5-expressing cells (Additional file 1: Fig. S2a, S2b). NTZ was therefore selected for additional analyses.

**Table 2:** IC_50_ values (μM) of the 5 most effective invasion-inhibiting drugs with limited or no implications in cancer or metastasis. PC-3 and DU 145 cells expressing different forms of KLF5 were used

	PC-3 (*KLF5* null)			DU 145 (*KLF5* null)		
	KLF5	KLF5^K369Q^	KLF5^K369R^	KLF5	KLF5^K369Q^	KLF5^K369R^
Furagin	17.3 ± 2.2	28.6 ± 1.9	100.9 ± 27.9	31.4 ± 4.0	63.3 ± 2.4	358.9 ± 127.2
Nifuratel	32.0 ± 1.6	18.6 ± 0.7	18.7 ± 1.0	7.1 ± 0.7	16.2 ± 0.8	14.0 ± 0.2
Nitazoxanide	7.0 ± 0.7	5.4 ± 0.5***	11.4 ± 1.0	55.6 ± 1.4	22.7 ± 2.3**	49.2 ± 10.1
Retapamulin	1.6 ± 0.3	12.6 ± 2.3	12.2 ± 1.1	13.7 ± 2.7	14.7 ± 1.5	11.1 ± 0.8

A wider range of NTZ concentrations was tested for their effect on PC-3-KQ and DU 145-KQ cells using the CCK-8 assay. As shown in Figs. S3a and S3b, NTZ did not affect cell number at lower concentrations (0–0.1 μM) but decreased cell number at higher concentrations. In the colony formation assay, NTZ reduced colony number with higher concentrations (Additional file 1: Fig. S3c-S3f).

### NTZ inhibits Ac-KLF5-induced bone metastasis of prostate cancer cells in both prevention and therapy modes

**Fig. 2:**
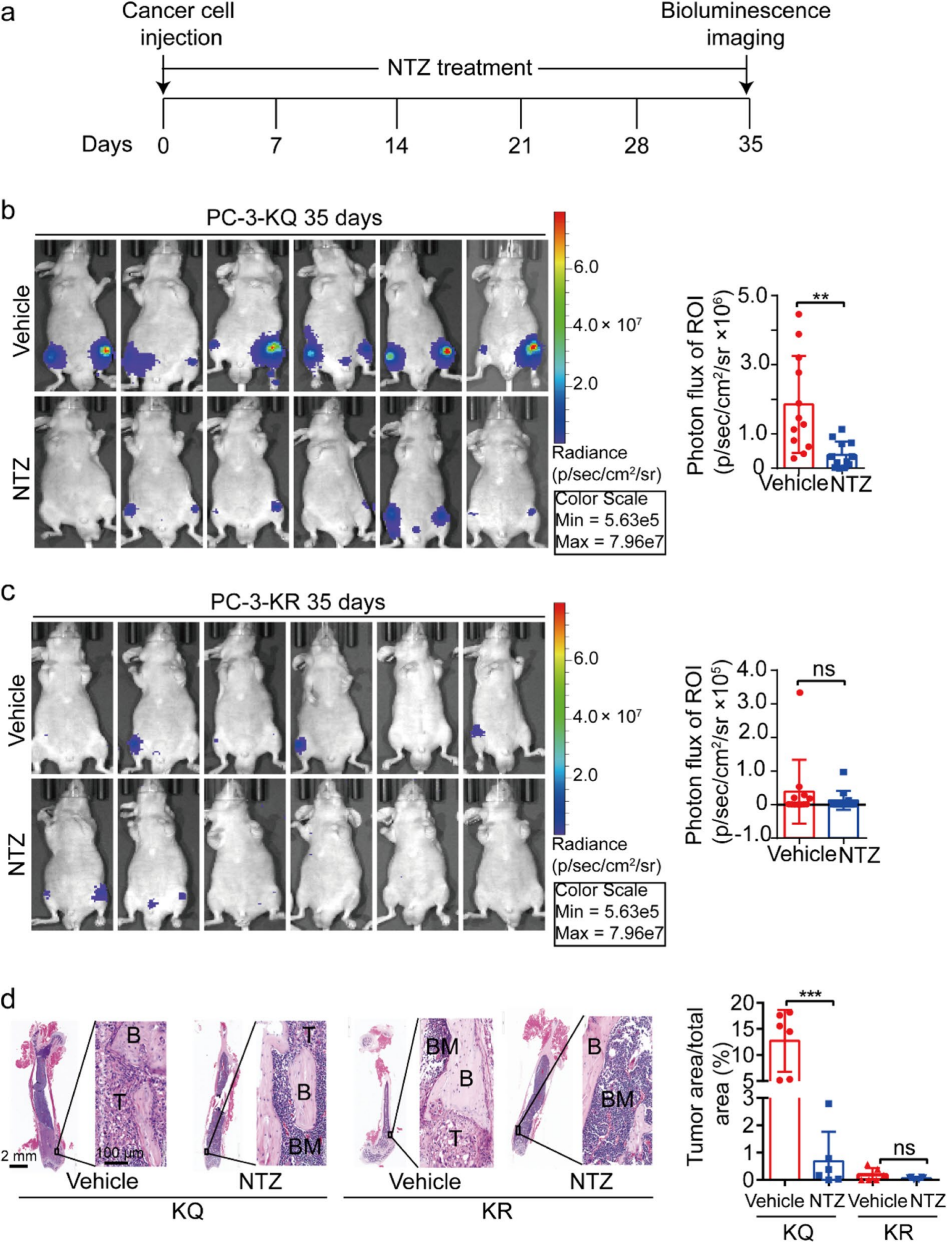
Nitazoxanide exerts a preventive effect on Ac-KLF5-induced bone metastasis in prostate cancer cells. **a** Diagram of the experimental timeline. On day 0, cancer cells (PC-3-KQ-Luc or PC-3-KR-Luc) were injected into mice via the tail caudal artery, while nitazoxanide was delivered via intragastric administration. Bioluminescence intensities were measured at day 35. **b**, **c** Bioluminescence images (left) of mice carrying PC-3-KQ cells (**b**) or PC-3-KR cells (**c**) and receiving nitazoxanide (NTZ) or the solvent control (vehicle). Bioluminescence intensities are indicated by photon flux of the region of interest (ROI) (right panel, each dot represents a leg of a mouse). *n* = 12 legs/group. **d** Nitazoxanide treatment significantly reduced the bone tumor areas formed by PC-3-KQ cells while not affecting those of PC-3-KR cells. Femur sections were stained with H&E, and areas of tumor cells and entire bone sections were measured and compared between the NTZ treatment and the control group. Representative images are shown on the left, and statistical outputs are shown on the right. *n* = 6 tumors for each group. B, trabecular bone regions; BM, bone marrow regions; T, tumor regions. Scale bar, 100 μm. All data are presented as mean ± SD. **p < 0.01; ***, *p* < 0.001; ns, not significantly different; as estimated by student's *t*-test

Both prevention and therapy modes were used to determine if NTZ inhibits Ac-KLF5-induced bone metastasis. In the prevention mode (Fig. 2a), prostate cancer cells expressing Ac-KLF5-mimicking KLF5^K369Q^ mutant (i.e., PC-3-KQ-Luc) were injected into mice via the caudal artery in the tail following a recently described procedure [26]. Five weeks after cell injection, bone metastasis was evident for PC-3-KQ-Luc cells, as the bioluminescence intensity was quite strong, according to a pilot experiment (data not shown).

In this model, administration of NTZ at the same time as tumor cell injection dramatically reduced bone metastasis, as indicated by the bioluminescence signals (Fig. 2b). Meanwhile, PC-3-KR-Luc cells generated much weaker bioluminescence signals (Fig. 2c). NTZ did not have a significant effect on the bioluminescence intensity of PC-3-KR-Luc cells (Fig. 2c). Bone histomorphometry of bone tissue sections revealed that NTZ significantly decreased tumor cell area in mice with PC-3-KQ-Luc cells (Fig. 2d). In contrast, mice with PC-3-KR-Luc cells had much smaller tumor areas, which were not significantly affected by NTZ (Fig. 2d).

The body weight of NTZ-treated mice did not show a noticeable change after 5 weeks in any of the groups (Additional file 1: Fig. S4a, S4b). In addition, no mice in any NTZ-treated group had severe adverse events, mortality, or noticeable histopathological changes in the heart, lung, liver, spleen, and kidney (Additional file 1: Fig. S4c).

**Fig. 3:**
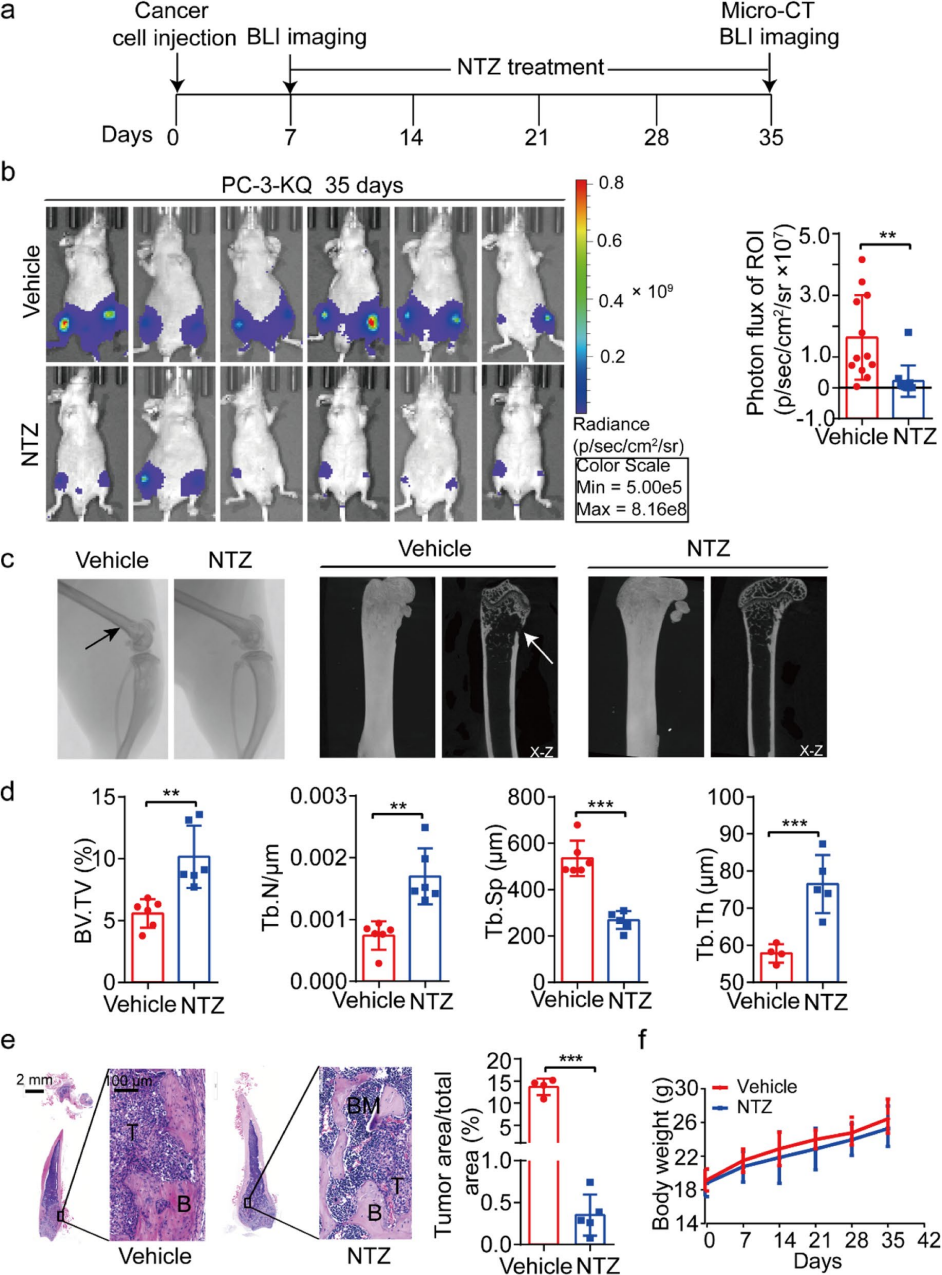
Nitazoxanide treatment inhibits osteolytic bone metastasis of prostate cancer in a mouse model. **a** Diagram of the experiment timeline. Tumor cells were allowed to colonize and grow for 7 days after injection via the caudal artery and before NTZ was administered via intragastric injection (six mice per group). **b** Bioluminescence images (left) and intensities (right) of mice after treatments with NTZ or vehicle for 28 days. *n* = 12 legs/group. **c** Representative micro-CT images of femurs and tibias (left) and micro-CT photomicrographs of femurs (X-Z at right). The white arrow points to the osteolysis formed in the femur metaphysis, which disappeared after NTZ treatment. **d** Quantifications of several parameters for bone microstructure, including bone volume per tissue volume (BV/TV, *n* = 6 femurs for each group), trabecular number (Tb.N, *n* = 6 femurs for each group), trabecular separation (Tb.Sp, *n* = 6 femurs in control group, *n* = 5 femurs in NTZ group), and trabecular thickness (Tb.Th, *n* = 4 femurs in control group, *n* = 5 femurs in NTZ group). **e** H&E staining of bone sections showing areas of tumor cells (T), bone (B), and bone marrow (BM) (left). The tumor area to the bone area ratio was calculated for each mouse and presented in the plot at right. Scale bar in H&E images, 100 μm. *n* = 4 femurs in control group, *n* = 5 femurs in NTZ group. **f** Body weights of mice at different time points after treatment with NTZ or vehicle. Data are presented as mean ± SD. **, *p* < 0.01; ***, *p* < 0.001; as estimated by Student’s *t*-test. NTZ, nitazoxanide

In the therapy mode, PC-3-KQ-Luc cells injected into mice were allowed to develop bone metastasis for 7 days before NTZ was administered. At 7 days after cell injection, bioluminescence signals were evident in most mice (Additional file 1: Fig. S5). Mice were divided into two groups according to mice’s bioluminescence signal intensities (Fig. 3a and Additional file 1: Fig. S5). Mice were sacrificed for analysis after 4 weeks of NTZ treatment. Similar to the finding from the preventive mode, NTZ treatment sharply decreased bioluminescence intensities (Fig. 3b). Micro-CT analysis demonstrated that, while bone lesions were evident in the control group, NTZ treatment significantly reduced such lesions (Fig. 3c). Quantitative analysis of bone parameters revealed that NTZ significantly increased BV/TV, Tb. N, and Tb. Th while potently decreased Tb. Sp (Fig. 3d). Quantification of H&E sections of bone tissues confirmed that NTZ significantly reduced the tumor area in the bone (Fig. 3e). The body weights of mice were not affected by NTZ (Fig. 3f).

### NTZ attenuates Ac-KLF5-induced osteoclasts formation

**Fig. 4:**
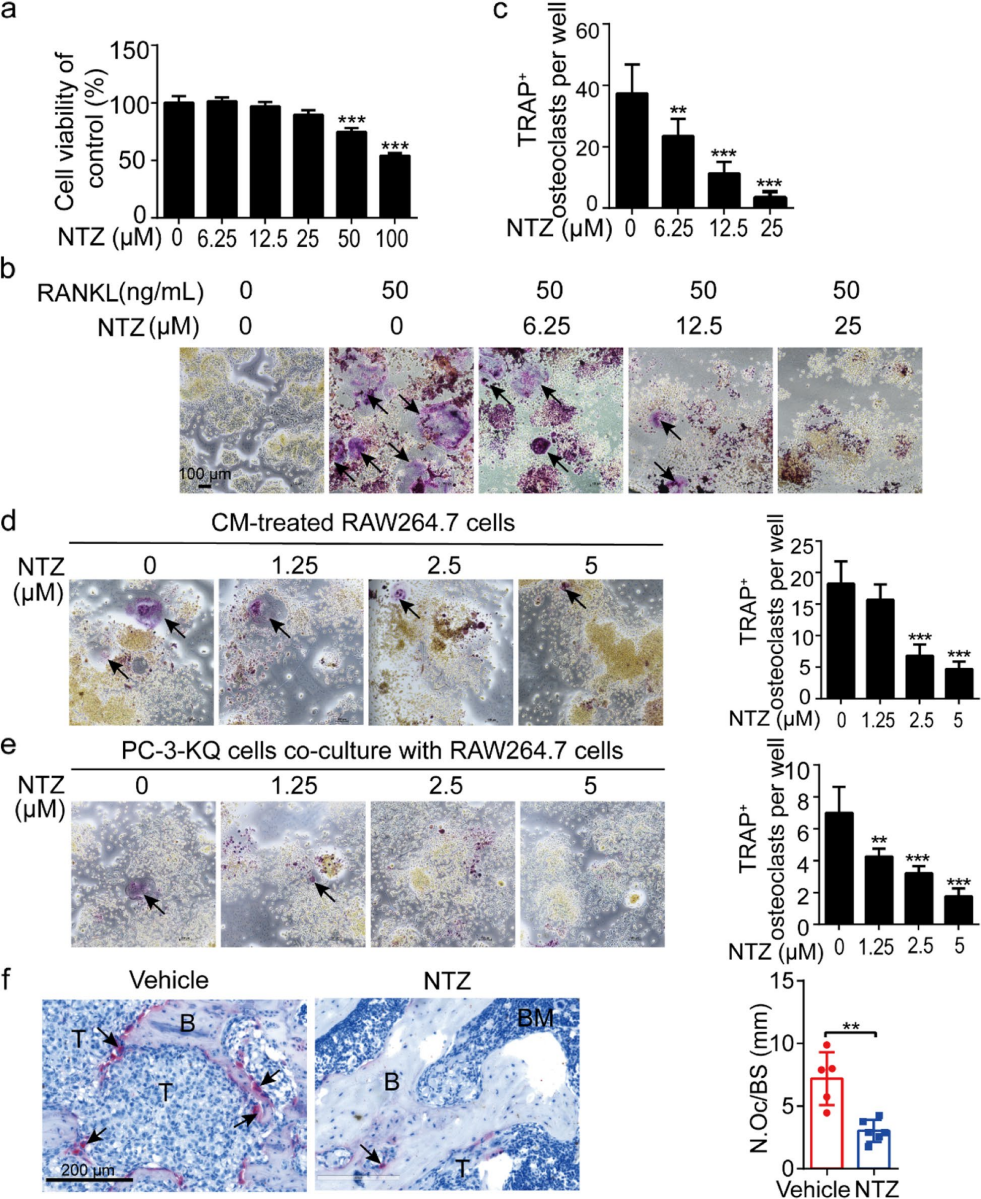
Nitazoxanide attenuates Ac-KLF5-induced osteoclast differentiation. **a** Effects of NTZ at different concentrations on the viability of RAW264.7 cells after 48 h treatment, as determined by the CCK8 assay. **b**, **c** NTZ inhibits osteoclast differentiation in a dose-dependent manner, as determined by the TRAP staining of RAW264.7 cells treated with RANKL (50 ng/mL) and various concentrations of NTZ for 5 days. Shown are representative images of TRAP^+^ and multinucleated cells (nuclei >3) (**b**) and their numbers per well (**c**). One-way ANOVA analysis was performed on the data. **d** NTZ inhibited osteoclast differentiation of RAW264.7 cells caused by conditioned medium (CM) from PC-3-KQ cells, as indicated by representative images of TRAP-stained cells (left) and the numbers of TRAP^+^ cells per well (right) after NTZ treatments. One-way ANOVA analysis was performed on the data. **e** NTZ inhibited osteoclast differentiation of RAW264.7 cells co-cultured with PC-3-KQ cells, as indicated by representative images of TRAP-stained cells (left) and the numbers of TRAP^+^ cells per well (right) after NTZ treatments. One-way ANOVA analysis was performed on the data. **f** NTZ decreased the number of Ac-KLF5-induced osteoclasts in mouse bones, as indicated by images of bone sections with TRAP^+^ cells (left) and the number of osteoclasts (N.Oc) per mm of bone surface (BS). Scale bar, 200 μm. Student’s *t*-test was performed. *n* = 5 femurs in vehicle group, *n* = 5 femurs in NTZ group. For all bar graphs, data are shown as mean ± SD. **, *p* < 0.01; ***, *p* < 0.001. T, tumor cells; B, bone; BM, bone marrow; NTZ, nitazoxanide; RANKL, receptor activator of the nuclear factor-κB ligand; TRAP, tartrate-resistant acid phosphatase

Osteoclast differentiation is an essential mechanism for Ac-KLF5 to induce bone metastasis in prostate cancer [23]. To determine whether NTZ attenuates Ac-KLF5-induced osteoclast differentiation, we first tested the cytotoxicity of NTZ in RAW264.7 cells, a cell line model of osteoclast differentiation. In the CCK-8 cell viability assay, NTZ did not exhibit detectable cytotoxicity in RAW264.7 cells at concentrations smaller than 25 μM after 48 h of treatment (Fig. 4a). During RANKL-induced osteoclast differentiation in RAW264.7 cells, which lasted for 7 days, NTZ treatment decreased the number of TRAP^+^ multinucleated cells in a dose-dependent manner (Fig. 4b, c). Even 6.25 and 12.5 μM of NTZ, which did not cause cytotoxicity in RAW264.7 cells (Fig. 4a), still significantly decreased the number of TRAP^+^ multinucleated cells (Fig. 4c). Therefore, NTZ likely attenuates the osteoclast differentiation.

We also collected conditioned media (CM) from PC-3-KQ cells treated with NTZ at varying concentrations (0, 1.25, 2.5, and 5 μM) for 36 h and used the CM to treat RAW264.7 cells in the presence of a more diluted RANKL (10 ng/mL) for 7 days. The number of TRAP^+^ multinucleated cells was significantly reduced by CM from cells treated with NTZ at 2.5 or 5 μM (Fig. 4d).

The third model for Ac-KLF5-induced osteoclasts was the co-culture of RAW264.7 cells with PC-3-KQ cells in the presence of NTZ (0, 1.25, 2.5, and 5 μM) for 7 days and the subsequent TRAP staining. The co-culture significantly increased, while NTZ treatment at all 3 concentrations significantly decreased the number of TRAP^+^ multinucleated cells (Fig. 4e). In mouse femurs, TRAP staining revealed that NTZ treatment significantly reduced the number of TRAP^+^ cells compared to the vehicle group (Fig. 4f).

MMP9 is critical in PCa metastasis [32], including bone resorption during bone metastasis [33]. We found that the Ac-KLF55-mimicking KLF5^K369Q^ significantly induced MMP9 expression at both protein and mRNA levels compared to acetylation-deficient KLF5^K369R^ (Additional file 1: Fig. S6a, S6b) and that NTZ decreased MMP9 expression in a dose-dependent manner in KLF5^K369Q^-expressing cells (Additional file 1: Fig. S6a, S6b). The decrease of MMP9 protein expression by NTZ was confirmed in KLF5^k369Q^-induced bone metastases by Immunohistochemical staining (Additional file 1: Fig. S6c). Therefore, NTZ-mediated inhibition of bone metastasis may also involve the downregulation of MMP9 by NTZ.

### NTZ reverses the effects of Ac-KLF5 on the expression of many genes

**Fig. 5:**
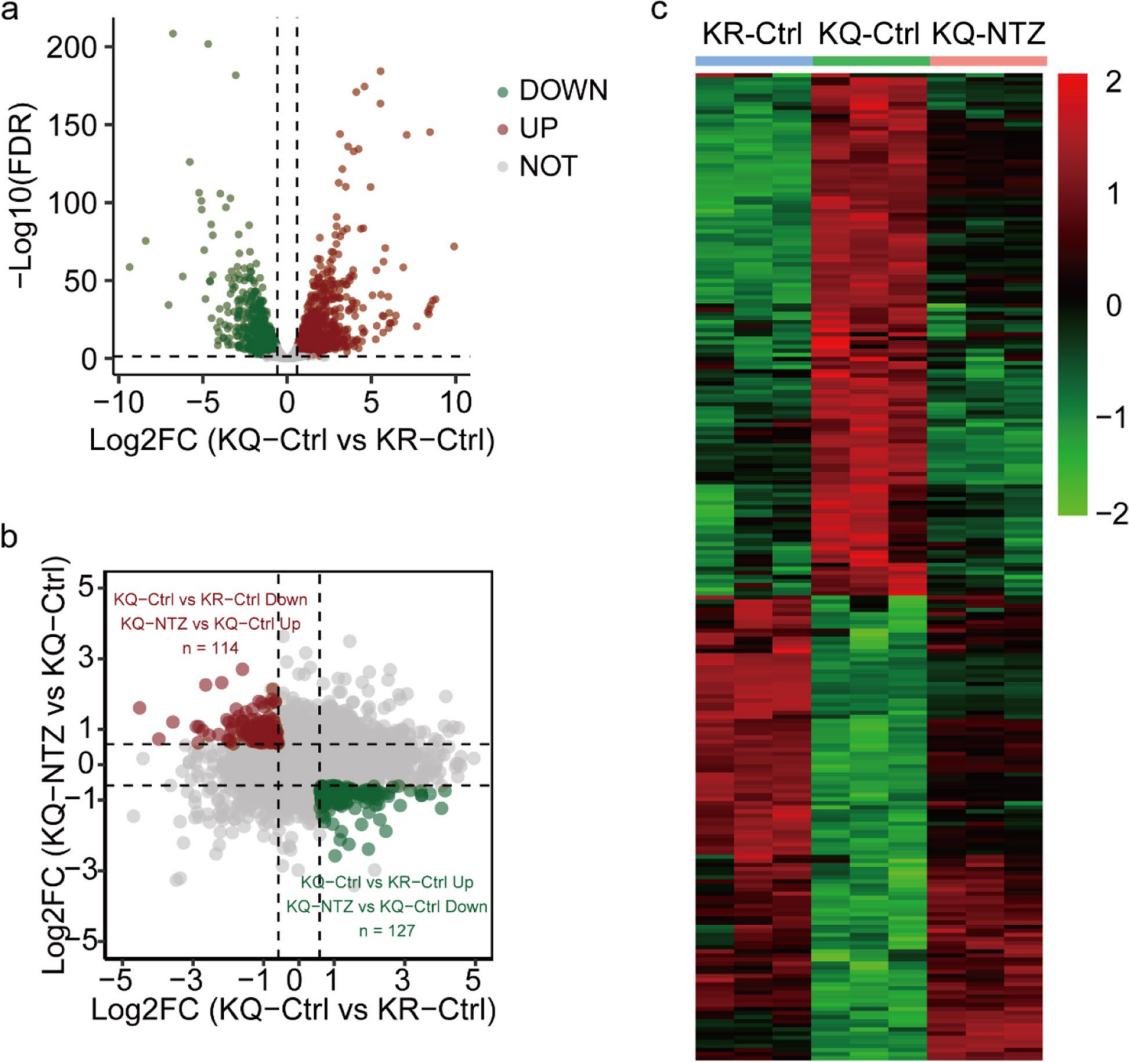
Nitazoxanide reverses the effects of Ac-KLF5 on the expression of many genes in PC-3 cells. **a** Volcano plots of all quantified genes from the gene expression profiles between the KLF5^K369Q^ and KLF5^K369R^ in PC-3 cells. **b** Scatter plot of differentially expressed genes (DEGs) between PC-3-KQ cells with and without NTZ treatment and DEGs between PC-3-KQ and PC-3-KR cells. Dots in the upper left quadrant represent genes that were downregulated by KLF5^K369Q^ but upregulated by NTZ, whereas dots in the lower right quadrant indicate genes that were upregulated by KLF5^K369Q^ but downregulated by NTZ in PC-3 cells. An adjusted *p* value <0.05 was used to define all altered genes. **c** Heatmap showing DEGs in panel A’s upper left and lower right quadrants. The color bar at the right indicates log2 fold changes (Log2FC)

To understand how NTZ suppresses KLF5^K369Q^-induced bone metastasis, RNA sequencing was performed using PC-3-KQ and PC-3-KR cells in the presence or absence of NTZ (5 μM). We focused on the genes whose expression patterns were modulated by KLF5^K369Q^, but the modulation of KLF5^K369Q^ was reversed by NTZ treatment. In total, there were 2836 differentially expressed genes between KLF5^K369Q^ and KLF5^K369R^, with 1779 upregulated and 1057 downregulated by KLF5^K369Q^ (Fig. 5a). KLF5^K369Q^-modulated expression patterns were reversed by NTZ treatment for 241 of 2836 differentially expressed genes. The 241 genes included 127 upregulated and 114 downregulated by KLF5^K369Q^ (Fig. 5b, c; Additional file 2: Table S3, Table S4).

### Expression levels of MYBL2, TIMM8A, and some other Ac-KLF5- and NTZ-responsive genes are associated with patient survival in prostate cancer

**Fig. 6:**
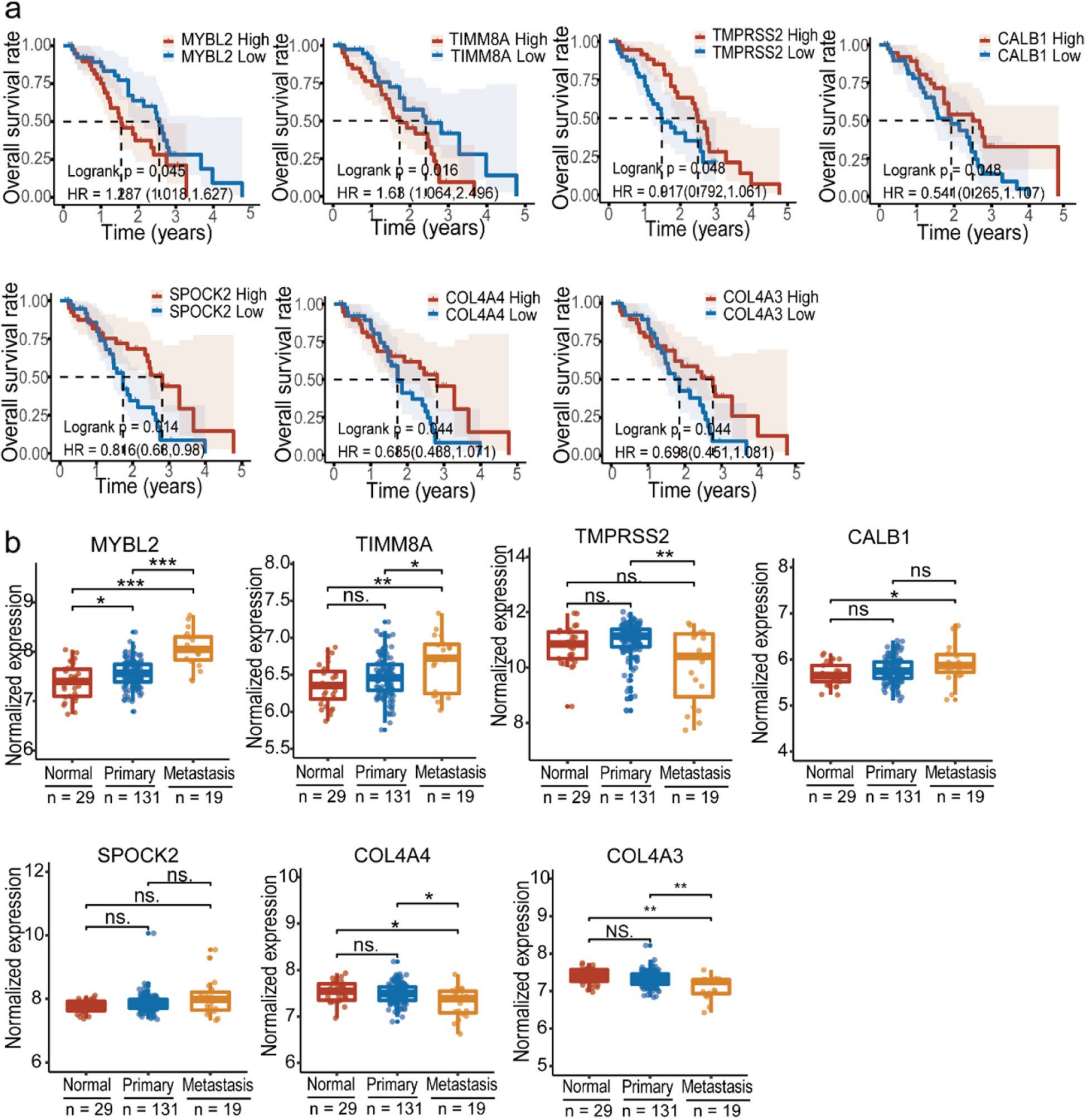
Association of Ac-KLF5- and NTZ-responsive genes with patient survival. **a** Higher expression levels of 7 genes correlate with overall survival (OS) in prostate cancer patients, as determined by the Kaplan-Meier analysis using the SU2C dataset. **b** Expression levels of 7 Ac-KLF5- and NTZ-responsive genes were analyzed using the GSE21034 dataset [29].

We evaluated the 241 KLF5^K369Q^-modulated and NTZ-responsive genes for the association of their expression levels with patient survival in prostate cancer using the SU2C database. Survival data were available for 119 of the 127 KLF5^K369Q^-upregulated and NTZ-downregulated genes and 111 of the 114 KLF5^K369Q^-downregulated and NTZ-upregulated genes (Additional file 2: Table S5). Of the 119 KLF5^K369Q^-upregulated and NTZ-downregulated genes, only the upregulation of *MYBL2* and *TIMM8A* was significantly associated with a worse patient OS (Fig. 6a), a pattern consistent with NTZ’s metastasis suppression function. The upregulation of 5 genes, including *INHBB*, *COL4A6*, *CACNG8*, *PIANP*, and *C2orf78*, was significantly associated with a better OS instead of a worse OS (Additional file 1: Fig. S7a). Upregulation of the remaining 112 genes did not significantly affect patient survival (Additional file 2: Table S5). Therefore, *MYBL2* and *TIMM8A* are more likely to mediate the induction of bone metastasis by Ac-KLF5.

In the GSE21034 dataset, expression levels of both *MYBL2* and *TIMM8A* were available in primary tumors, visceral metastases, and bone metastases of prostate cancer [29]. Whereas both *MYBL2* and *TIMM8A* were expressed at higher levels in metastases than primary tumors when visceral and bone metastases were combined (Fig. 6b), only *MYBL2* showed a significantly higher expression level in bone metastasis than primary tumors when bone metastases were analyzed separately (Additional file 1: Fig. S7b).

Of the 111 KLF5^K369Q^-downregulated and NTZ-upregulated genes in the SU2C dataset, higher levels of *TMPRSS2*, *CALB1*, *SPOCK2*, *COL4A4*, and *COL4A3* were significantly associated with a better OS (Additional file 1: Fig. 6a). However, higher levels of 2 genes (FTH1 and BDH1) were associated with a worse patient OS (Additional file 1: Fig. S7a).

### Ac-KLF5 activates but NTZ attenuates the transcription of MYBL2 in prostate cancer cells

**Fig. 7:**
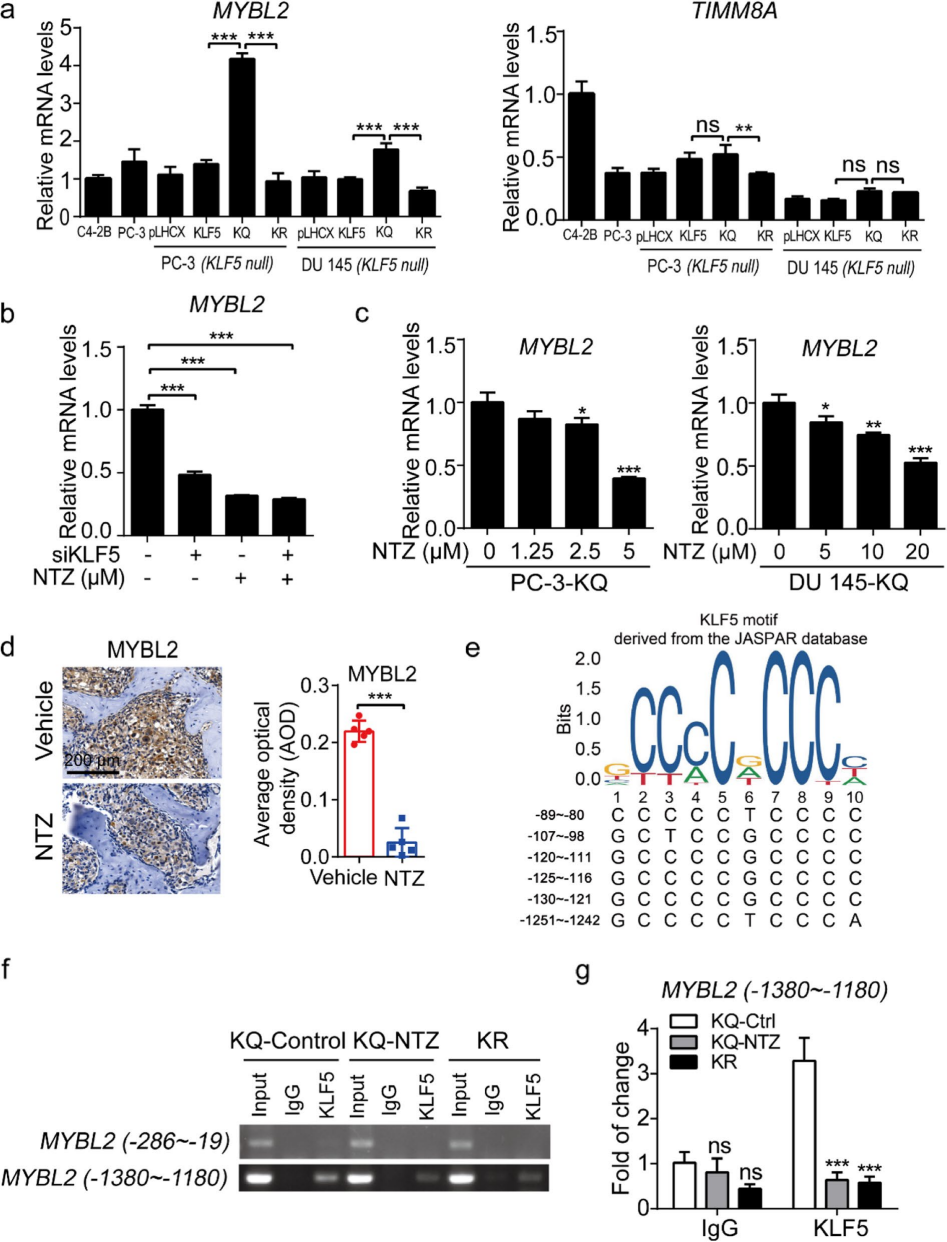
Nitazoxanide attenuates the upregulation of MYBL2 expression by Ac-KLF5 in prostate cancer cells. **a** Expression of MYBL2 and TIMM8A in PC-3 and DU 145 cells expressing different forms of KLF5, as determined using qPCR. C4-2B and parental PC-3 cell lines were used as controls, and GAPDH was used as a loading control. Statistical analysis was performed using one-way ANOVA with multiple comparisons. **b** Cells were transfected with 50 nM siKLF5 concomitantly with or without NTZ (5 μM) treatment for 24 h and then processed real-time qPCR. Statistical analysis was performed using one-way ANOVA with multiple comparisons. **c** NTZ downregulated MYBL2 expression in PC-3-KQ cells and DU 145-KQ cells at mRNA levels, as detected by qPCR. NTZ treatments were at indicated concentrations for 48 h. Statistical analysis was performed using one-way ANOVA with multiple comparisons. **d** NTZ treatment in mice reduced MYBL2 expression in PC-3-KQ bone metastasis, as detected by IHC staining and indicated by IHC images and intensities of staining signals. *n* = 5 tumors for each group. Student’s *t*-test was performed. Scale bar, 200 μm. **e** The *MYBL2* gene promoter contained multiple potential KLF5 binding sites, as predicted by the JASPAR program. **f**, **g** NTZ had different effects on the binding of Ac-KLF5 to different Ac-KLF5-binding sites of the *MYBL2* gene promoter, as determined by ChIP and regular RT-PCR (**f**) or real-time qPCR (**g**) using PC-3-KQ cells treated with NTZ for 48 h and PC-3-KR cells. All bar graphs are shown as mean ± SD of three independent experiments. *, *p* < 0.05; **, *p* < 0.01; ***, *p* < 0.001; ns, no statistically significant

We further tested whether Ac-KLF5 transcriptionally regulates *MYBL2* and *TIMM8A*. In PC-3 and DU 145 cells expressing different forms of KLF5, quantitative RT-PCR revealed that *MYBL2* was significantly induced by KLF5^K369Q^ in both PC-3 and DU 145 cells, whereas the induction of *TIMM8A* was not significant (Fig. 7a; Additional file 1: Fig. S8a). When *KLF5* was knocked down by siRNAs in PC-3-KQ cells, the expression of *MYBL2* was significantly reduced at both mRNA and protein levels (Fig. 7b; Additional file 1: Fig. S8b, S8c). MYBL2 was recently demonstrated to promote metastasis and castration resistance of prostate cancer [34]. Therefore, we focused on the transactivational regulation of *MYBL2* by KLF5^K369Q^.

In both PC-3 and DU 145 cells expressing KLF5^K369Q^, NTZ treatment reduced the expression of *MYBL2* at both mRNA and protein in a dose- or time-dependent manner (Fig. 7c; Additional file 1: Fig. S8d, Fig. S8e). As revealed by immunohistochemical staining, NTZ treatment in mice also significantly decreased MYBL2 protein expression in bone tissues (Fig. 7d).

To further test the effect of NTZ on Ac-KLF5-induced transcription of *MYBL2*, we analyzed the promoter sequence of *MYBL2* using the Jaspar Software to identify potential KLF5 binding sites (Fig. 7e) and performed ChIP-PCR using primers spanning the 6 potential KLF5 binding sites with the highest binding scores. For the 5 potential binding sites between −80 and −130 nucleotides, no evident binding was detected using 2 pairs of primers (Fig. 7f). For the possible binding site between -1242 and -1251 nucleotides, CHIP-PCR revealed evident occupancy of KLF5^K369Q^ but not KLF5^K369R^ on *MYBL2* promoter. NTZ treatment significantly decreased KLF5^K369Q^-bound DNA (Fig. 7f and g).

### NTZ binds to the KLF5 protein regardless of its acetylation status

**Fig. 8:**
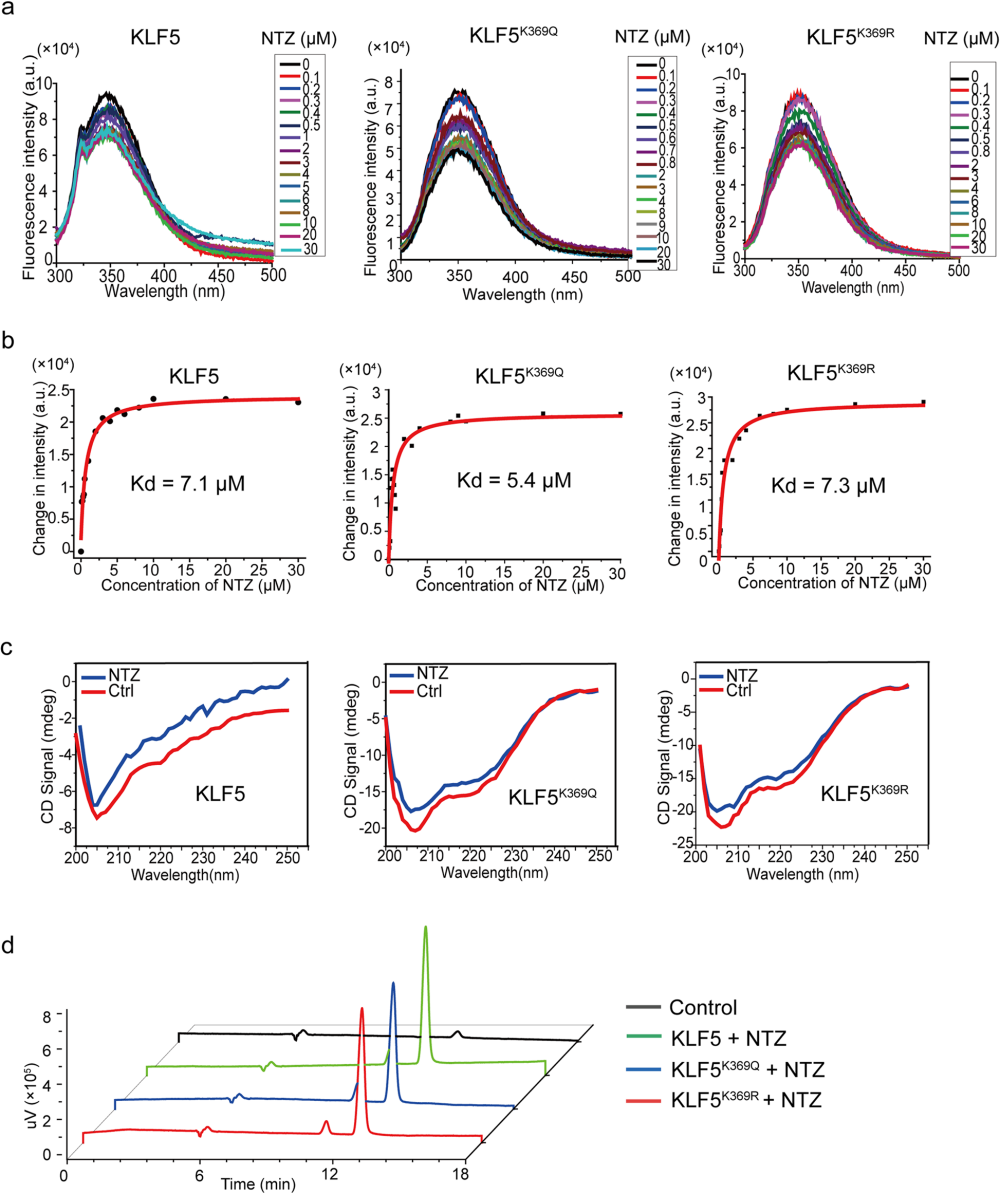
NTZ binds to KLF5 protein regardless of its acetylation status. **a** Fluorescence intensities of KLF5, KLF5^K369Q^, and KLF5^K369R^ proteins were measured after incubation with different concentrations of NTZ. **b** Calculation of the Kd values for dissociation constant using the Origin software and by fitting the fluorescence quenching data. **c** Circular dichroism (CD) spectra of KLF5, KLF5^K369Q^, and KLF5^K369R^ incubated with NTZ. The concentrations of both proteins and NTZ are 0.2 μM. **d** Binding of NTZ to the purified different forms of KLF5 proteins using HPLC analysis

We tested whether NTZ directly binds to the KLF5 protein using different assays (Fig. 8). In the fluorescence quenching assay, NTZ reduced the fluorescence intensity of KLF5, KLF5^K369Q^, and KLF5^K369R^ in a dose-dependent manner (Fig. 8a), with a binding constant Kd of 7.2, 5.4, and 7.3 μM, respectively (Fig. 8b). In the CD spectroscopy, all three forms of KLF5 exhibited negative peaks between 205 nm and 220 nm, which indicates modifications in a protein’s secondary structure [35]. NTZ treatment decreased the intensity of these negative peaks without changing the spectrum shape (Fig. 8c), suggesting a loss of α-helix structures. HPLC analysis also confirmed that NTZ binds to the KLF5 protein regardless of KLF5 acetylation status (Fig. 8d).

In these drug-protein binding assays, we used the synthetic retinoid Am80 as a negative control, as Am80 binds to RARα to promote its association with KLF5 in gene regulation without direct binding to KLF5 [36]. Am80 did not bind to any form of the KLF5 protein in the fluorescence quenching assay, as the Kd was greater than 30 μM (Additional file 1: Fig. S9a and S9b). Am80 did not change the secondary structure of KLF5 either, regardless of NTZ treatment (Additional file 1: Fig. S9c).

## Discussion

Findings in this study present nitazoxanide (NTZ), a drug currently approved for treating parasitic and viral infections [37, 38], as a potential therapeutic agent for treating PCa bone metastasis. The direct evidence for this conclusion came from the animal experiments, where NTZ treatment drastically suppressed the formation of bone metastases in an established mouse model, i.e., of KLF5^K369Q^-induced bone metastasis (Figs. 2 and 3). The inhibition of bone metastasis was effective in both the preventive and therapeutic modes, as similar efficacies were observed when NTZ was administered at the same time of cell inoculation (Fig. 2) or one week after (Fig. 3).

Cancers of the prostate, breast, and lung often metastasize to the bone [39–41]. Whereas breast and lung cancers mostly undergo osteolytic metastasis, prostate cancer causes both osteolytic and osteoblastic lesions [39–41]. Various factors affect the balance between pro-osteoclastic and pro-osteoblastic activities of prostate cancer cells [40, 41]. For example, in the bone microenvironment, prostate cancer cells secret bone-related soluble factors such as RANKL and M-CSF to activate osteoclasts; and osteoclasts in turn release TGF-β and IGF-1 to support the growth of prostate cancer cells [40, 41]. In addition, osteoclasts release osteolytic factors such as parathyroid hormone-related peptide (PTHrP) to promote osteoblast differentiation and survival [39, 40]. Therefore, osteoclast differentiation plays an important role in prostate cancer bone metastasis, and inhibiting osteoclast differentiation may effectively attenuate prostate cancer bone metastasis. Our previous study demonstrated that osteoclast differentiation is a cellular mechanism for KLF5^K369Q^ to induce osteolytic bone metastasis in prostate cancer [23]. Even in C4-2B cells, which primarily promote osteoblastic lesions in the bone [42], ectopic expression of KLF5^K369Q^ or KLF5 still caused osteolytic bone lesion [23]. Consistent with its therapeutic effect on bone metastasis, NTZ treatment indeed attenuated KLF5^K369Q^-induced osteoclast differentiation. Such an inhibitory effect was first demonstrated in an in vitro model of osteoclast differentiation, i.e., RAW264.7 cells treated with RANKL or co-cultured with KLF5^K369Q^-expressing cancer cells (Fig. 4b–e). NTZ’s inhibitory effect on osteoclasts was also confirmed in the mouse model (Fig. 4f). It is worth noting that even at concentrations that did not show cytotoxicity to RAW264.7 cells (Fig. 4a), NTZ still decreased osteoclasts (Fig. 4b, c). However, considering that prostate cancer more often induces osteoblastic lesions, it is meaningful to investigate whether NTZ also inhibits the differentiation and function of osteoblasts.

Inhibition of KLF5^K369Q^-induced osteoclast differentiation in vitro and in vivo is consistent with a recent study in which NTZ reduces bone loss in ovariectomized mice by inhibiting RANKL-induced osteoclastogenesis [43].

At the molecular level, it appears that NTZ directly modulates KLF5^K369Q^’s function in gene transcription in inhibiting bone metastasis. KLF5 is a transcription factor, and KLF5^K369Q^-mediated bone metastasis is mediated by a series of genes transcriptionally regulated by KLF5^K369Q^, including CXCR4, IL11, and others [23]. KLF5^K369Q^ upregulated or downregulated 2836 genes compared to KLF5^K369R^ (Fig. 5a). NTZ treatment reversed 241 genes’ upregulation or downregulation by KLF5^K369Q^ (Fig. 5b, c; Additional file 2: Table S3, Table S4). These findings indicate that NTZ changes the function of KLF5^K369Q^ in many genes’ transcription.

Multiple genes could mediate the effect of NTZ on KLF5^K369Q^-mediated bone metastasis. For example, two Ac-KLF5-upregulated and NTZ-downregulated genes, *MYBL2* and *TIMM8A*, were upregulated in human prostate cancer. Their upregulation was significantly associated with worse overall survival in patients with prostate cancer (Fig. 6a). There were also 5 KLF5^K369Q^-downregulated and NTZ-upregulated genes whose lower expression levels in human prostate cancers were also significantly associated with worse overall survival, including *TMPRSS2*, *CALB1*, *SPOCK2*, *COL4A4*, and *COL4A3* (Fig. 6a). These 7 genes could more likely impact KLF5^K369Q^’s promoting role in bone metastasis, in addition to CXCR4 and IL11 described in a previous study [23].

Among the 7 KLF5^K369Q^-regulated and NTZ-responsive genes, the *MYBL2* (Myb-related protein B) is particularly interesting, as a previous study has demonstrated that MYBL2 promotes castration-resistant growth and bone metastasis of prostate cancer [34]. In addition, targeting MYBL2 blocks bone metastasis in prostate cancer cells, and higher MYBL2 expression levels are associated with higher tumor stage, higher tumor grade, higher risk of metastatic relapse, and worse prognosis in patients with prostate cancer [34]. MYBL2 regulates cell cycle progression, survival, and differentiation in cancer cells [44]. We found that the *MYBL2* gene was indeed transcriptionally activated by KLF5^K369Q^ in prostate cancer cells. For example, KLF5^K369Q^ upregulated while its silencing reduced *MYBL2* expression, and KLF5^K369Q^ bound to specific sequences of the *MYBL2* promoter to activate its transcription while KLF5^K369R^ did not (Fig. 7; Additional file 1: Fig. S8). In addition, NTZ treatment reduced the expression of MYBL2 in both cultured cells and tumors grown in mice and compromised the binding of KLF5^K369Q^ to the *MYBL2* promoter (Fig. 7).

Also worth noting is that KLF5^K369Q^ upregulated MMP9 expression, and the upregulation was attenuated by NTZ treatment (Additional file 2: Table S4; Additional file 1: Fig. S6). Activated osteoclasts produce acid and proteinases such as matrix metalloproteinases (MMPs) to degrade the bone matrix, releasing more TGF-β and other growth factors in the bone. Such factors in turn stimulate bone metastasis, resulting in the so-called vicious circle [45]. Inhibition of MMP9 expression by NTZ in KLF5^K369Q^-expressing cells could thus also contribute to the inhibition of bone metastasis by NTZ.

NTZ appears to directly act on the KLF5 protein to modulate its functions, as revealed by different drug-protein binding assays (Fig. 8). While NTZ binds to the KLF5 molecule, the binding does not appear to be specific to KLF5^K369Q^, as KLF5 and KLF5^K369R^ also showed similar binding capacities (Fig. 8). It remains to be determined how NTZ acts on KLF5, and whether such an act directly impacts the function of KLF5 in gene transcription.

The inhibitory effect of NTZ on bone metastasis could apply to other types of malignancies where bone metastasis frequently occurs, and TGF-β is an inducer, including the carcinomas of the breast, lung, and prostate and multiple myeloma [46, 47]. We previously reported that TGF-β induces the acetylation of KLF5 in epithelial cells, and acetylation of KLF5 is essential for TGF-β to regulate gene expression and multiple cellular processes such as cell proliferation and EMT [17, 18, 20, 23, 48]. The Ac-KLF5-mimicking KLF5^K369Q^ mutant maintains EMT, promotes cell invasion, and induces bone metastasis in prostate cancer cells [23]. Therefore, the therapeutic effect of NTZ on KLF5^K369Q^-induced bone metastasis established in this study should also apply to TGF-β-induced bone metastasis in other types of malignancies.

Other drugs that also suppressed the invasion of KLF5^K369Q^-expressing cells in the cell spheroid invasion assay could also be capable of suppressing bone metastasis. The screening strategy was developed by Cribbes et al. previously [24]. The discovery of NTZ’s suppressive activity in bone metastasis validates this screening strategy with KLF5^K369Q^-expressing cells (Fig. 1). In addition to NTZ, 5 other drugs also inhibited cell spheroid invasion by more than 50% at 0.1 μM. They included nifuratel, mitomycin C, ronidazole, retapamulin, and furagin (Fig. 1d, Table 1). While mitomycin C is widely used in the treatment of metastasis [49, 50] and ronidazole acts as a veterinary agent, none of the remaining 3 drugs has been implicated in cancer metastasis in the literature. These 3 drugs are thus worth testing for their potential therapeutic roles in bone metastasis.

NTZ is approved for the treatment of various infectious diseases, including protozoal, anthelmintic, viral, and bacterial infections [51]. Although our study is the first to demonstrate a therapeutic effect of NTZ on the bone metastasis of prostate cancer, its anticancer activity has been reported in recent studies in different types of cancers involving various molecular mechanisms. For example, it suppressed colon tumor growth likely by inducing cell cycle arrest and altering the expression of multiple molecules [52, 53]; it suppressed ovarian cancer growth partially by inhibiting the protein disulfide isomerase (PDI) activity [54]; it inhibited sphere formation in 3D cultures of HCC and CRC cells involving the inhibition of OXPHOS [55, 56]; and it suppressed glioma growth by causing the G2/M cell cycle arrest, inducing apoptosis, and inhibiting autophagy likely via CDK1 inhibition, ING1 upregulation, etc. [57, 58]. NTZ could also prevent the induction of mammary tumors by MNU in rats [59].

At the molecular level, previous studies suggest that NTZ could impact the functions of multiple molecules and modulate various signaling pathways [51]. For example, NTZ was identified as an MYC inhibitor in breast cancer cells using an HTS screening system [60]; NTZ could act as a moderate inhibitor of the STAT3 pathway [61]; inhibition of the Wnt signaling activity by NTZ could be independent of APC but involves PAD2 targeting and subsequent increase in the deamination and turnover of β-catenin in colon cancer cells [62]. Bioinformatic analyses suggest that in HCC cells, NTZ could target many molecules, biological processes, and signaling pathways [63]. Induction of cell death by NTZ and some of its derivatives also involves targeting the 20S proteasome [64].

## Conclusions

In summary, we adopted the recently developed spheroid invasion screening assay to identify FDA-approved drugs that target bone metastasis induced by acetylated-KLF5 in prostate cancer. Six of the 1987 drugs inhibited cell spheroid invasion by more than 50% at 0.1 μM. They included nitazoxanide, nifuratel, mitomycin C, ronidazole, retapamulin, and furagin. Functional experiments revealed that NTZ suppressed KLF5^K369Q^-induced bone metastasis in both preventive and therapeutic modes in mice. NTZ inhibited osteoclastogenesis, a cellular process responsible for KLF5^K369Q^-induced bone metastasis. Mechanistically, NTZ bound to the KLF5 molecule and reversed KLF5^K369Q^’s function in the transcription of many genes. Two KLF5^K369Q^-upregulated and NTZ-downregulated genes, *MYBL2* and *TIMM8A*, were upregulated in human prostate cancer, and the upregulation was associated with worse patient survival. Upregulation of *MYBL2* by KLF5^K369Q^ involved direct promoter binding, and NTZ attenuated the binding. These findings present NTZ as a potential therapeutic agent for bone metastasis induced by Ac-KLF5 in prostate cancer.

## Supplementary Information

**Additional file 1: Figure S1.** Comparison of migration and invasion ability among different forms of KLF5 expressing cells, related to Fig. 1. **Figure S2.** The effect of NTZ on acetylated (KQ) and non-mutated KLF5 cell invasion, related to Fig. 1. **Figure S3.** The effect of NTZ on cell proliferation in acetylated KLF5 expressing cells, related to Fig. 1. **Figure S4.** NTZ caused no noticeable toxicity in vivo, related to Fig. 2. **Figure S5.** Represensitive BL images of each mouse on day 7 (left) and BL intensity analysis (right), related to Fig. 3. **Figure S6.** NTZ downregulated acetylated-KLF5 induced MMP9 expression. **Figure S7.** Differential genes between KQ-Ctrl and KR-Ctrl groups and Overall survival analysis of 7 differential genes, related Fig. 5 and Fig. 6. **Figure S8.** Nitazoxanide reduces acetylated-KLF5 -induced MYBL2 production, related to Fig. 7. **Figure S9.** Am80 as a negative control does not bind to KLF5, KLF5^K369Q^, and KLF5^K369R^ proteins, related to Fig. 8.

**Additional file 2: Table S1.** Secondary drug screening of 87 hit compounds. **Table S2.** Third drug screening of 25 hit compounds. **Table S3.** Differential genes affected by NTZ in RNA-Seq. **Table S4.** TPM of PC-3 cells with KR and KQ in presence or absence of NTZ in RNA-Seq. **Table S5.** The significance of overall survival of NTZ-downregulated genes or NTZ-upregulated genes in SU2C database.

**Additional file 3.** The uncropped immunoblot or gels images.

## Data Availability

Data supporting this study, including the compound library screen results and RNA sequencing analysis results, are included in the supplementary data files. The dataset analyzed during the current study is available via NCBI Gene Expression Omnibus (GEO) repository with the accession no. GSE21034, https://identifiers.org/geo:GSE21034. The raw sequence data in this study have been deposited in the GEO with accession number GSE216126 and the web link for this study is https://www.ncbi.nlm.nih.gov/gds/?term=GSE216126.

## Abbreviations

KLF5: Krüppel-like factor 5
Ac-KLF5: Acetylated KLF5
KQ: Acetylated KLF5
KR: Deacetylated KLF5
PCa: Prostate cancer
mCRPC: Metastatic castration-resistant prostate cancer
TGF-β: Transforming growth factor-β
EMT: Epithelial-mesenchymal transformation
DMSO: Dimethyl sulfoxide
FDA: Food and Drug Administration
NTZ: Nitazoxanide
CCK-8: Cell Counting Kit-8
CMC: Sodium carboxymethyl cellulose
RANKL: Receptor activator of nuclear factor-κB
TRAP: Tartrate-resistant acid phosphatase
FDR: False discovery rate
SU2C/PCF: Stand Up to Cancer/Prostate Cancer Foundation
SRE: Skeletal-related events
IC50: Half-maximal inhibitory concentration
OD: Optical density
