# Supplementary material for: Nitazoxanide inhibits acetylated KLF5-induced bone metastasis by modulating KLF5 function in prostate cancer
Source: BMC Med. 2023 Feb 21;21:68. doi: 10.1186/s12916-023-02763-4 (PMC9945734; doi:10.1186/s12916-023-02763-4)
Supplement: Supplementary file 1 — Additional file 1: Figure S1. Comparison of migration and invasion ability among different forms of KLF5 expressing cells, related to Fig. 1. Figure S2. The effect of NTZ on acetylated (KQ) and non-mutated KLF5 cell invasion, related to Fig. 1. Figure S3. The effect of NTZ on cell proliferation in acetylated KLF5 expressing cells, related to Fig. 1. Figure S4. NTZ caused no noticeable toxicity in vivo, related to Fig. 2. Figure S5. Represensitive BL images of each mouse on day 7 (left) and BL intensity analysis (right), related to Fig. 3. Figure S6. NTZ downregulated acetylated-KLF5 induced MMP9 expression. Figure S7. Differential genes between KQ-Ctrl and KR-Ctrl groups and Overall survival analysis of 7 differential genes, related Fig. 5 and Fig. 6. Figure S8. Nitazoxanide reduces acetylated-KLF5 -induced MYBL2 production, related to Fig. 7. Figure S9. Am80 as a negative control does not bind to KLF5, KLF5K369Q, and KLF5K369R proteins, related to Fig. 8. [file 12916_2023_2763_MOESM1_ESM.docx]

**Supplementary information**

**Nitazoxanide inhibits acetylated KLF5-induced bone metastasis by modulating KLF5 function in prostate cancer**

**Huang et al.**

| **Contents** | **Pages** |
| --- | --- |
| Additional file 1 |  |
| Figure S1 | 2 |
| Figure S2 | 3 |
| Figure S3 | 4 |
| Figure S4 | 5 |
| Figure S5 | 5 |
| Figure S6 | 6 |
| Figure S7 | 7 |
| Figure S8 | 8 |
| Figure S9 | 9 |
| Additional file 2 |  |
| Table S1 | 10-13 |
| Table S2 | 14 |
| Table S3 | 15-24 |
| Table S4 | 25-34 |
| Table S5 | 35-39 |
| Additional file 3 |  |
| uncropped blots. | 40 |

**Ⅰ. Additional file 1**

**
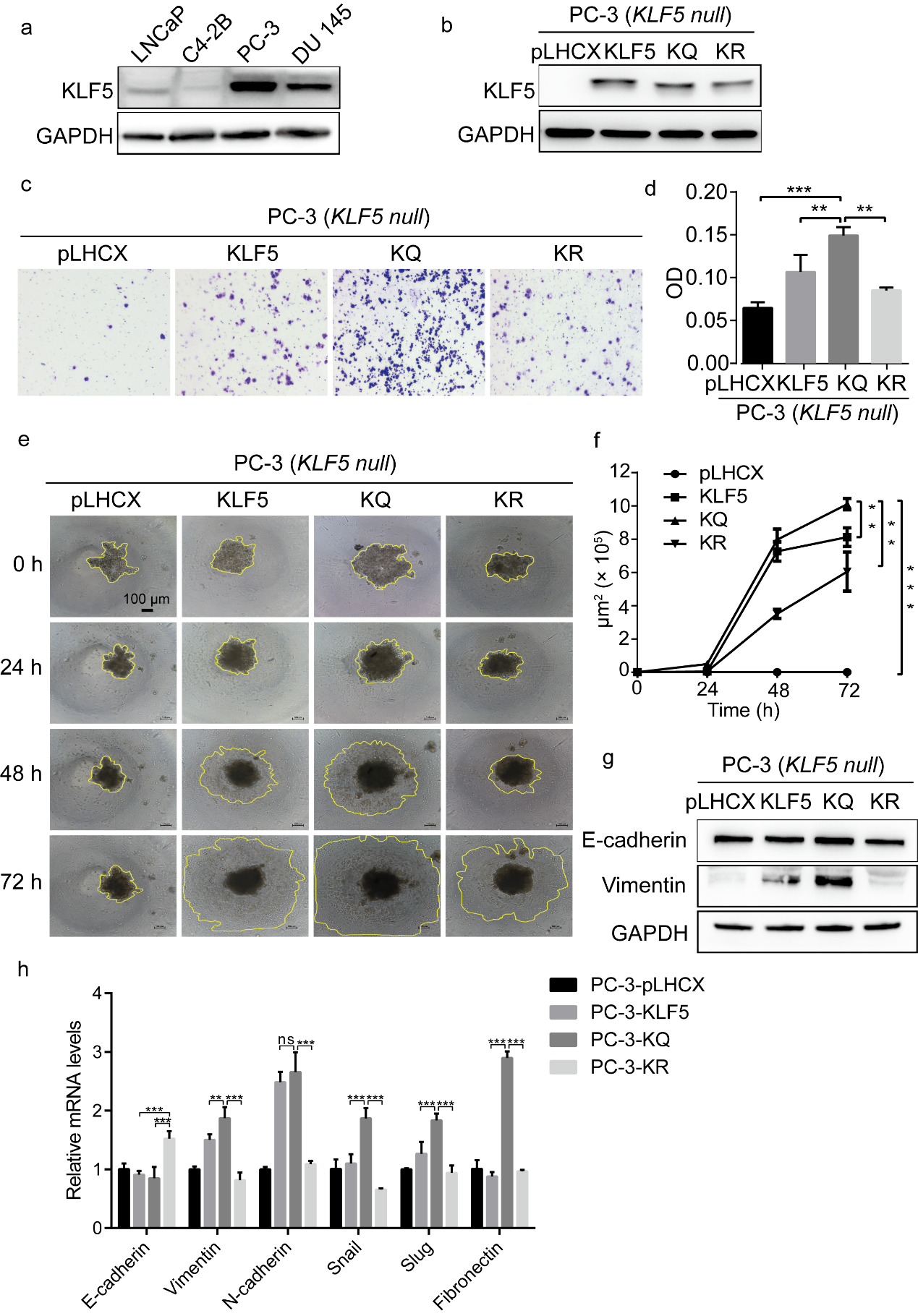
**

**Figure S1. Comparison of migration and invasion ability among different forms of KLF5 expressing cells.** (a) The expression level of KLF5 in PCa cells (LNCaP, C4-2B, PC-3 and DU 145). (b) The expression level of KLF5 in different forms of KLF5 expressing cells. (c) Representative images of migration of PC-3 cells expressing different forms of KLF5 (pLHCX, KLF5, KQ, and KR). (d) The OD value indirectly reflects the number of cells that PC-3-pLHCX, PC-3-KLF5, PC-3-KQ and PC-3-KR cells pass through the chamber. (e) Representative images of PC-3-pLHCX, PC-3-KLF5, PC-3-KQ and PC-3-KR spheroids invaded at 0, 24, 48 and 72 hours, respectively. (f) The line graph of the invasion area of PC-3-pLHCX, PC-3-KLF5, PC-3-KQ and PC-3-KR cell spheres at 0, 24, 48 and 72 hours, respectively. (g) The expression of E-cadherin and Vimentin in different forms of KLF5 expressing PC-3 cell spheroids. GAPDH was set as a loading control. (h) Relative mRNA levels of EMT markers (E-cadherin, Vimentin, N-cadherin, Snail, Slug and Fibronectin) among different forms of KLF5 expressing PC-3 cells. Data are shown as mean ± SD of three samples per group. ns, not significantly different ; **, *p* < 0.01; ***, *p* < 0.001; as estimated by one-way ANOVA.


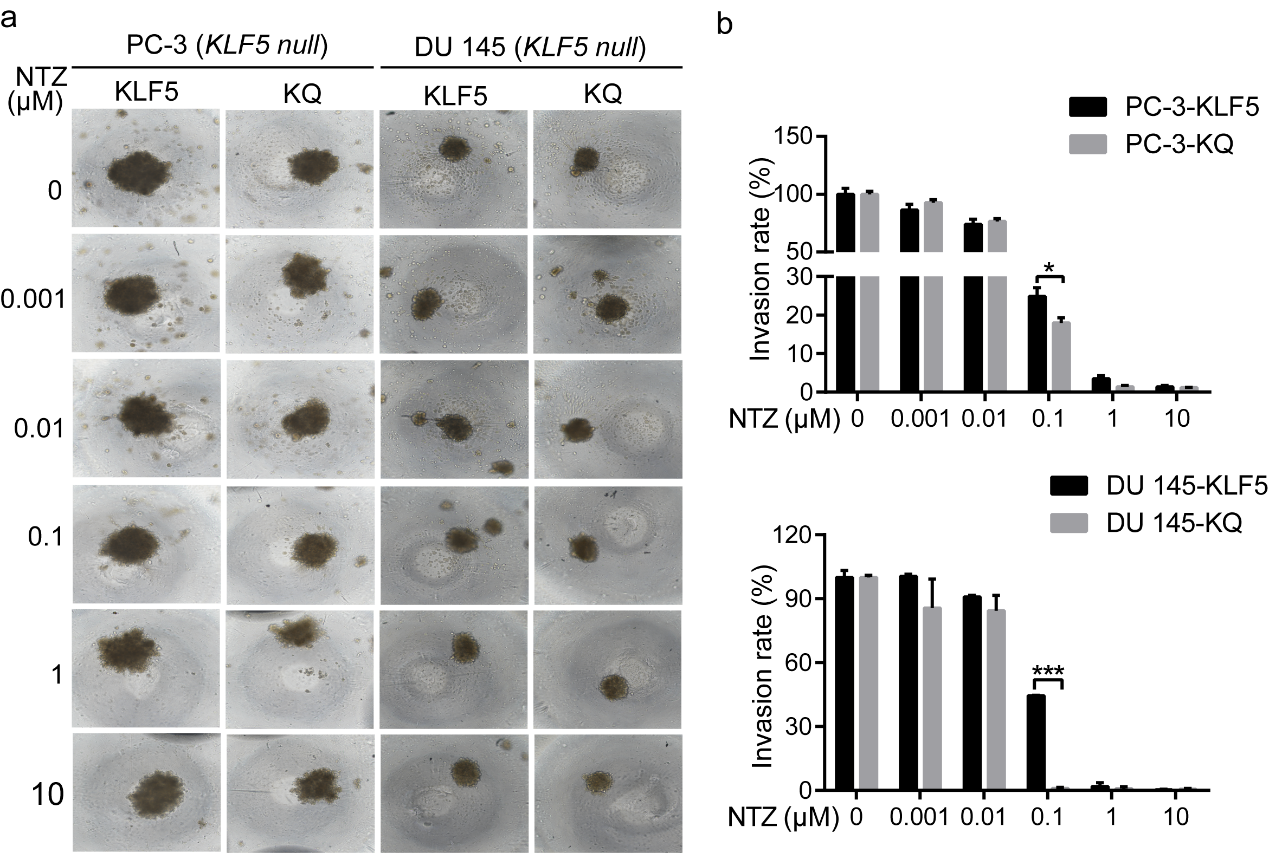


**Figure S2. The effect of NTZ on acetylated (KQ) and non-mutated KLF5 cell invasion.** (a) The effect of NTZ on the invasion of PC-3-KLF5, PC-3-KQ, DU 145-KLF5 and DU 145-KQ cell spheroids at different concentrations (0-10 μM) and quantitative analysis (b). *, *p* < 0.05; ***, *p* < 0.001.


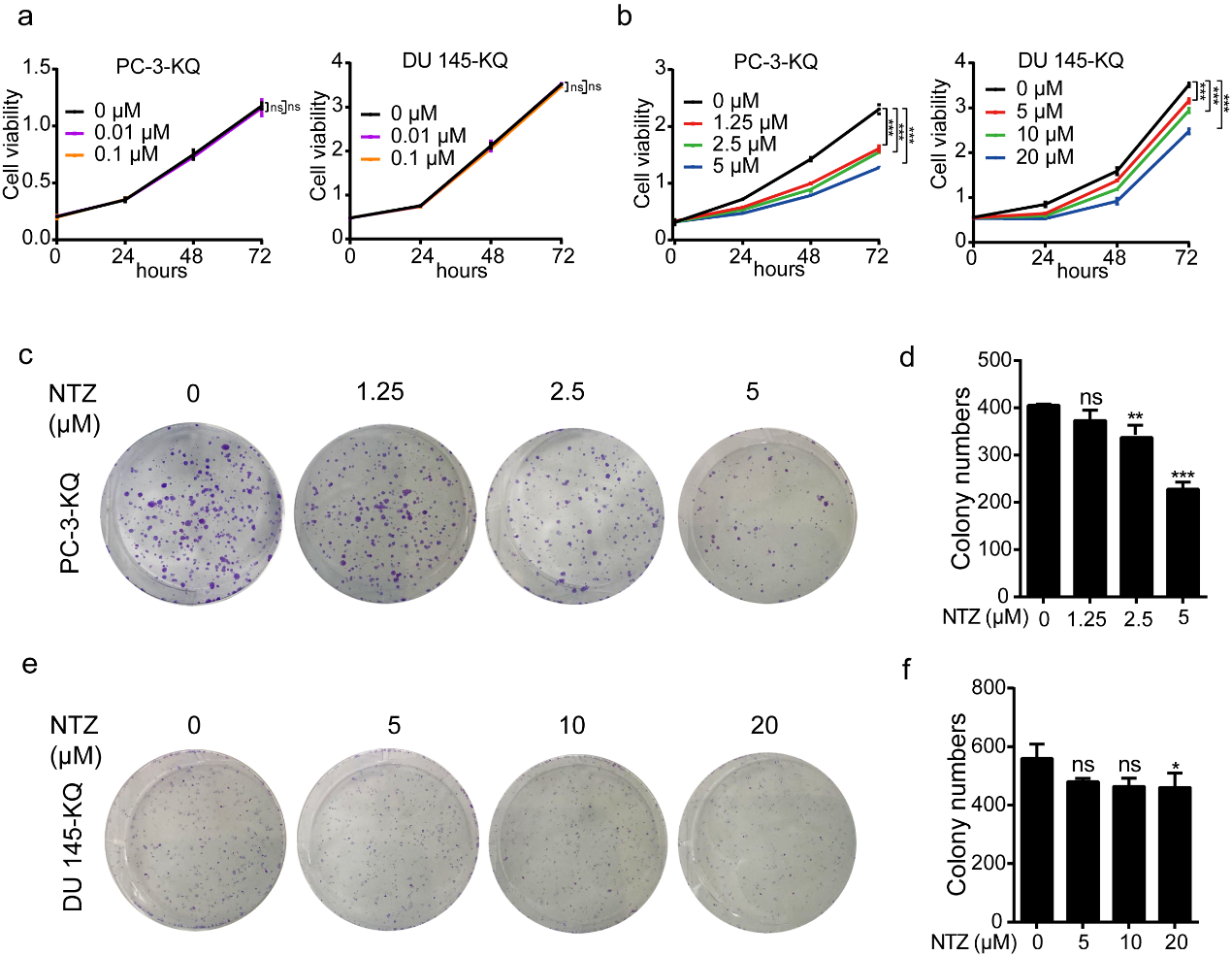


**Figure S3. The effect of NTZ on cell proliferation in acetylated KLF5 expressing cells.** (a) CCK8 assay measured the effect of low concentrations (0-0.01 μM) of NTZ on acetylated KLF5 expressing cells, as determined from 0 to 72 hours. (b) The effect of NTZ on cell growth in Ac-KLF5 expressing cells with indicated concentrations (0-5 μM or 0-20 μM). (c) Representative images of the effect of NTZ on colony formation in PC-3-KQ cells and quantitative analysis (d). Cells were treated NTZ with indicated concentrations (0-5 μM) for 8 days. (e) Representative images of the effect of NTZ on colony formation in DU 145-KQ cells (f). Cells were treated NTZ with indicated concentrations (0-20 μM) for 8 days. ns, not significantly different ; *, *p* < 0.05; **, *p* < 0.01; ***, *p* < 0.001; as estimated by one-way ANOVA.

**
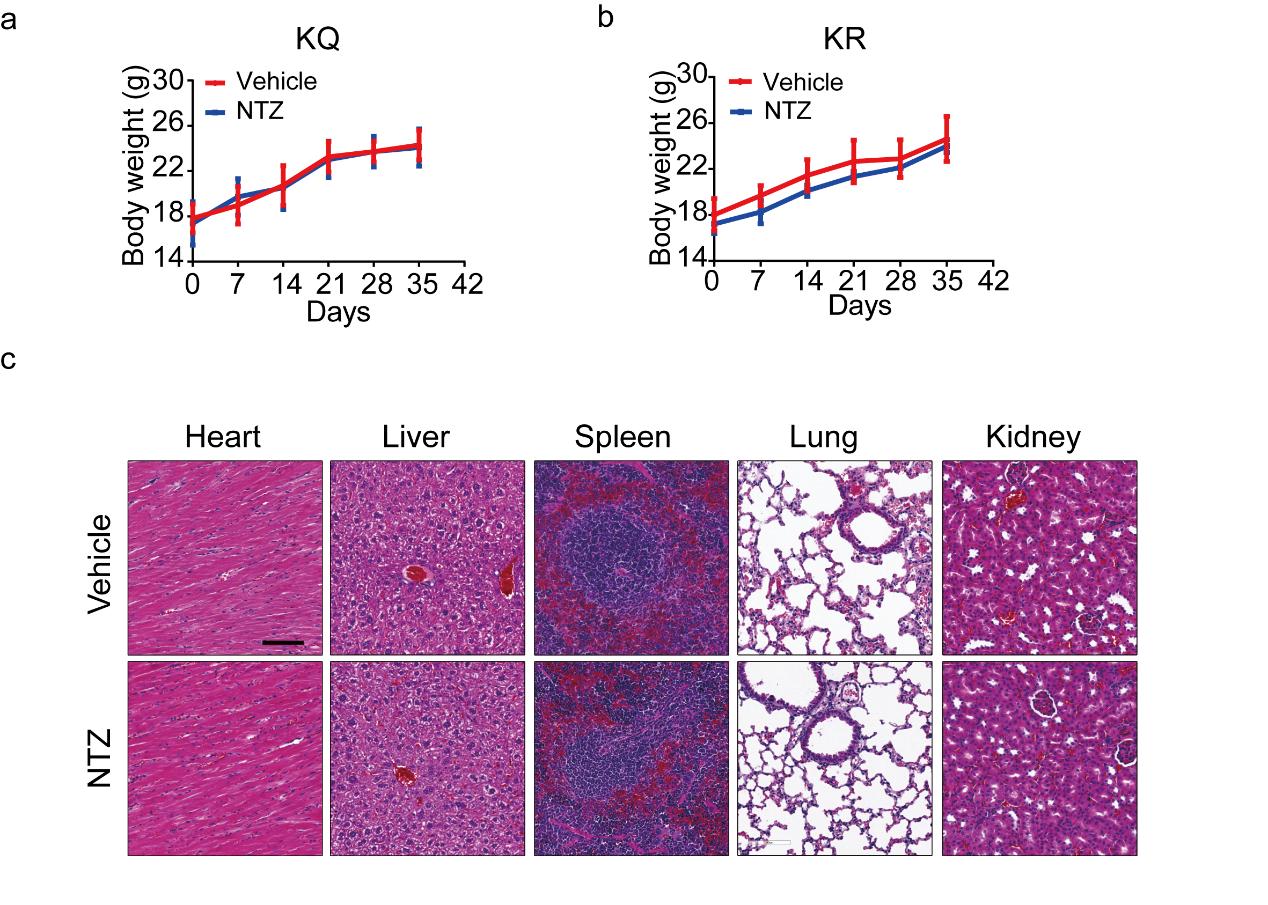
**

**Figure S4. NTZ caused no noticeable toxicity *in vivo*.** (a, b) The body weight change of mice in the PC-3-KQ-Luc groups or the PC-3-KQ-Luc groups at different time points during administration. n = 6 mice for each group. (c) H&E staining of main organs (Heart, Lung, Liver, Spleen and Kidney) from the mice. Scale bar, 100 μm.

**
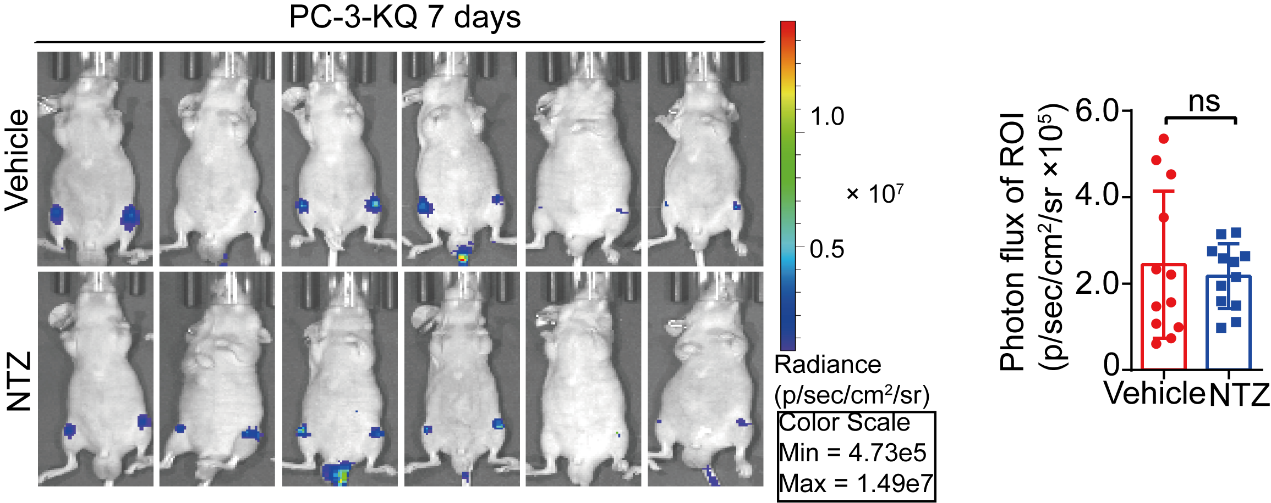
**

**Figure S5. Represensitive BL images of each mouse on day 7 (left) and BL intensity analysis (right).** Bioluminescence intensities are indicated by photon flux of the region of interest (ROI) (right panel, each dot represents a leg of a mouse). n = 12 legs/group. ns, not significantly different; as estimated by student's *t*-test.

**
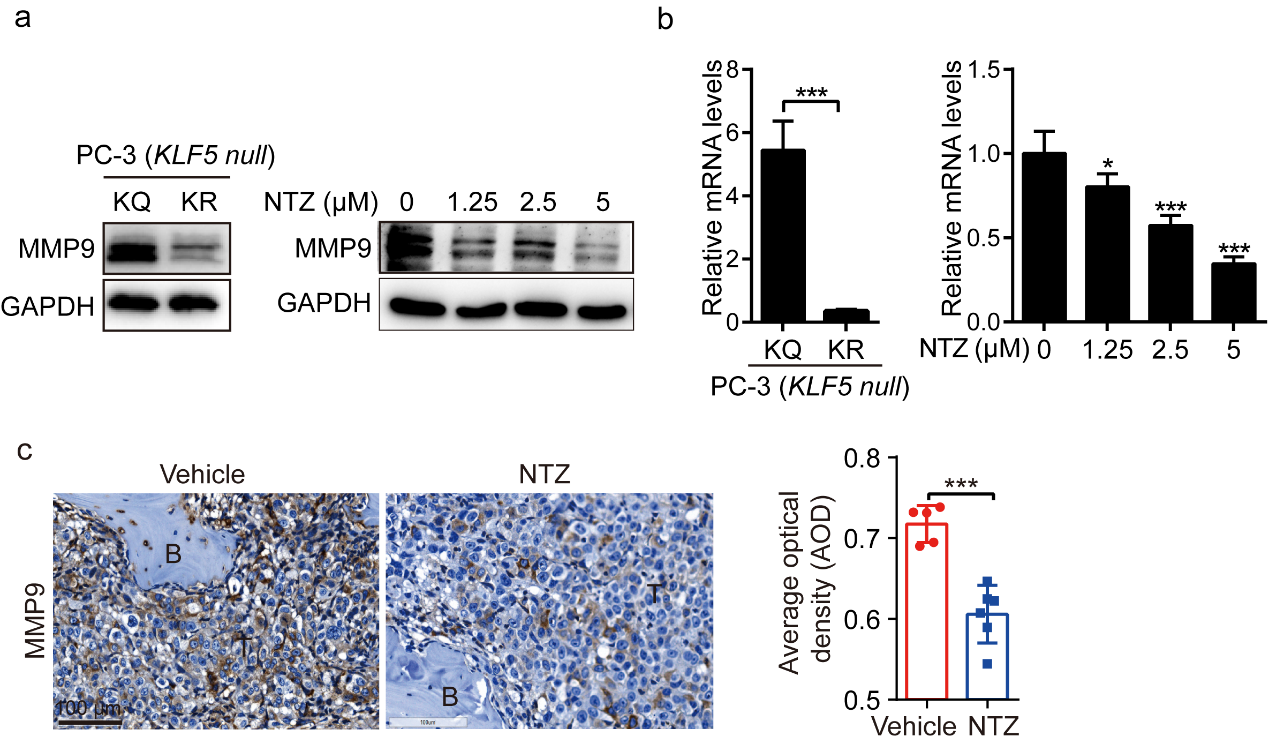
**

**Figure S6. NTZ downregulated acetylated-KLF5 induced MMP9 expression.** (a) Comparison of MMP9 protein expression between PC-3-KQ and PC-3-KR cells and the effect of NTZ on MMP9 expression in PC-3-KQ cells. (b) Comparison of MMP9 mRNA levels between PC-3-KQ and PC-3-KR cells and the effect of NTZ on MMP9 mRNA expression in PC-3-KQ cells. (c) NTZ decreased the expression of MMP9 in PC-3-KQ tumor cells, as indicated by representative images of IHC-stained tissue sections (left) and the quantifications of staining intensities (right). Scale bar, 100 μm. Student's *t*-test was performed. n = 5 femurs in vehicle group, n = 6 femurs in NTZ group. *, *p* < 0.05; ***, *p* < 0.001. T, tumor cells; B, bone.


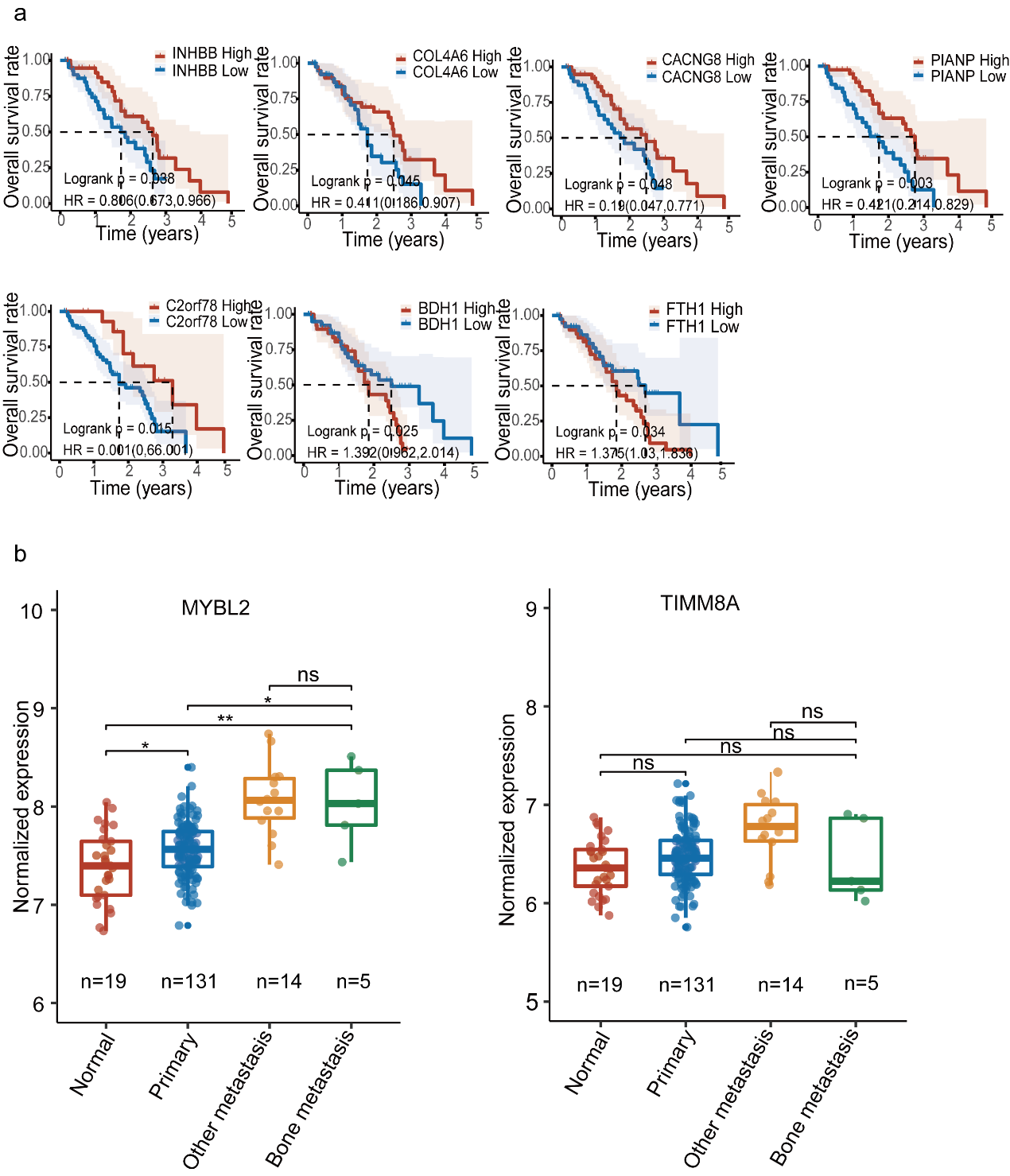


**Figure S7**. **Differential genes between KQ-Ctrl and KR-Ctrl groups and Overall survival analysis of 7 differential genes.** (a) Overall survival analysis of 7 differential genes, including *INHBB*, *COL4A6*, *CACNG8*, *PIANP*, *C2orf78*, *BDH1*, and *FTH1* in SU2C database. (b) Expression levels of *MYBL2* and *TIMM8A* were analyzed more specifically in GSE21034 dataset, including normal, primary, other metastasis and bone metastasis. *, *p* < 0.05; **, *p* < 0.01; ns, no statistically significant.

**
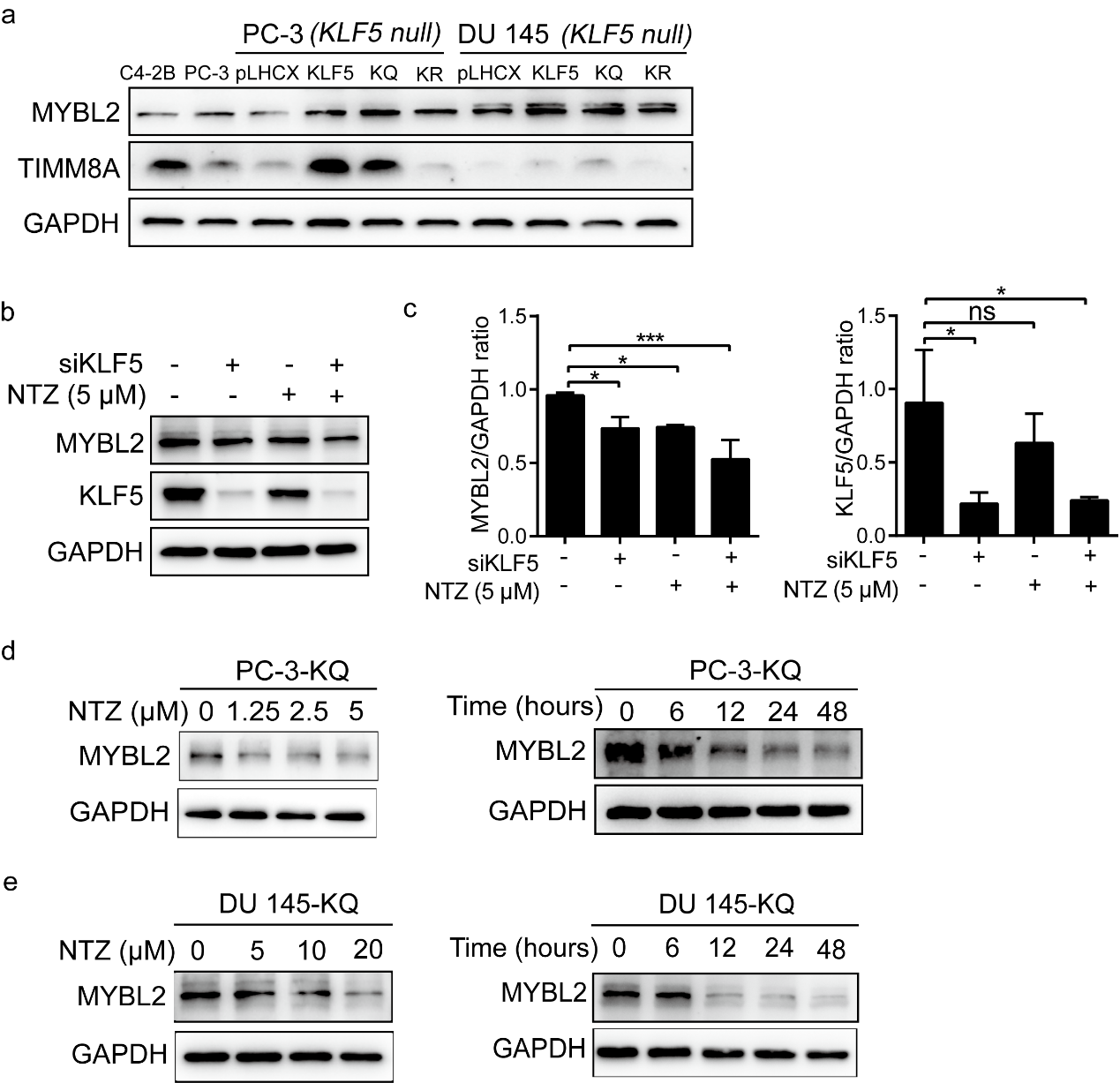
**

**Figure S8. Nitazoxanide reduces acetylated-KLF5 -induced MYBL2 production.** (a) Western blot analysis of the protein level of MYBL2 and TIMM8A in parental C4-2B, PC-3 and different forms of KLF5 expressing cells. (b) Cells were transfected with 50 nM siKLF5 concomitantly with or without NTZ (5μM) treatment for 24 hours and then processed western blot and their quantitative analysis (c). (d) NTZ downregulated MYBL2 expression in PC-3-KQ cells at protein levels, as detected by western blotting. NTZ treatments were at indicated concentrations for 48 hours or at 5 µM for indicated time points. (e) Representative images of the effects of NTZ on MYBL2 protein at the indicated concentrations (0-20 μM) and time points (0-48 hours) in DU 145-KQ cells. *, *p* < 0.05; ***, *p* < 0.001; ns, no statistically significant.

**
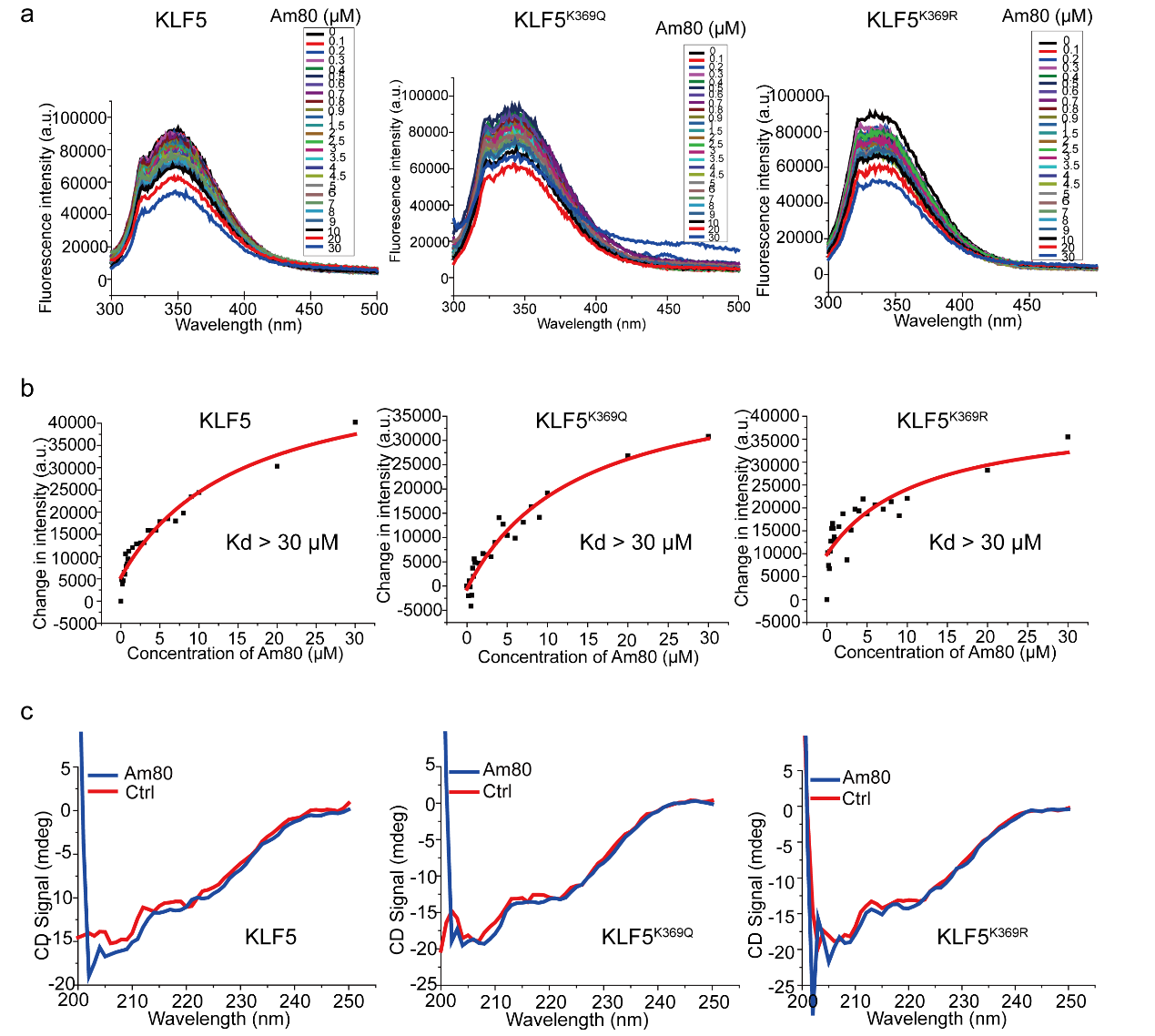
**

**Figure S9. Am80 as a negative control does not bind to KLF5, KLF5^K369Q^, and KLF5^K369R^ proteins.** (a) Fluorescence intensity of KLF5, KLF5^K369Q^, and KLF5^K369R^ with the addition of different concentrations of Am80. (b) Fluorescence quencing data were fitted to obtain Kd using Origin software. (c) CD spectra of KLF5, KLF5^K369Q^, and KLF5^K369R^ in the absence and presence of Am80. The concentrations of proteins and Am80 were 0.2 μM and 0.2 μM, respectively.
