# Supplementary material for: Nitazoxanide inhibits acetylated KLF5-induced bone metastasis by modulating KLF5 function in prostate cancer
Source: BMC Med. 2023 Feb 21;21:68. doi: 10.1186/s12916-023-02763-4 (PMC9945734; doi:10.1186/s12916-023-02763-4)
Supplement: Supplementary file 2 — Additional file 2: Table S1. Secondary drug screening of 87 hit compounds. Table S2. Third drug screening of 25 hit compounds. Table S3. Differential genes affected by NTZ in RNA-Seq. Table S4. TPM of PC-3 cells with KR and KQ in presence or absence of NTZ in RNA-Seq. Table S5. The significance of overall survival of NTZ-downregulated genes or NTZ-upregulated genes in SU2C database. [file 12916_2023_2763_MOESM2_ESM.docx]

**Ⅱ. Additional file 2**

**Table S1. Secondary drug screening of 87 hit compounds.**

|  | **Invasion rates (%) of each drug’s multiple concentrations (μM)** | | | | | |  | **Invasion rates (%) of each drug’s multiple concentrations (μM)** | | | | | |
| --- | --- | --- | --- | --- | --- | --- | --- | --- | --- | --- | --- | --- | --- |
| Drug name | 10 | 1 | 0.1 | 0.01 | 0.01 | 0 | Drug name | 10 | 1 | 0.1 | 0.01 | 0.01 | 0 |
| Fusidic acid (sodium salt) | 0.70 | 68.14 | 87.85 | 92.69 | 89.70 | 100.00 | Retapamulin | 4.68 | 7.28 | 7.18 | 7.43 | 34.60 | 100.00 |
| Demeclocycline (hydrochloride) | 4.21 | 41.75 | 81.08 | 92.92 | 97.24 | 100.00 | Tigecycline (tetramesylate) | 8.35 | 4.75 | 4.93 | 59.60 | 77.93 | 100.00 |
| Ornidazole (Levo-) | 4.84 | 71.01 | 87.04 | 90.43 | 93.59 | 100.00 | Furagin | 2.18 | 3.36 | 5.95 | 24.79 | 42.22 | 100.00 |
| Sorafenib | 43.30 | 76.25 | 91.93 | 91.87 | 101.48 | 100.00 | Ozenoxacin | 4.14 | 88.16 | 94.32 | 96.05 | 100.15 | 100.00 |
| Nifuratel | 0.26 | 8.43 | 60.61 | 69.96 | 82.60 | 100.00 | Tetracycline (hydrochloride) | 2.14 | 67.27 | 83.74 | 84.87 | 95.46 | 100.00 |
| Daptomycin | 1.26 | 88.47 | 95.71 | 96.29 | 96.99 | 100.00 | Tilorone (dihydrochloride) | 9.81 | 93.22 | 94.30 | 95.38 | 99.11 | 100.00 |
| Azathioprine | 33.55 | 68.34 | 80.04 | 85.19 | 91.60 | 100.00 | Dasatinib | 15.07 | 37.10 | 37.67 | 46.24 | 49.71 | 100.00 |
| Tamoxifen (Citrate) | 1.00 | 85.65 | 91.22 | 94.34 | 97.97 | 100.00 | Ixazomib citrate | 1.55 | 1.95 | 26.91 | 69.78 | 81.29 | 100.00 |
| Ponatinib | 17.74 | 91.02 | 94.85 | 94.38 | 94.47 | 100.00 | Thiamphenicol | 6.21 | 71.47 | 91.71 | 90.88 | 92.00 | 100.00 |
| Garenoxacin (Mesylate hydrate) | 48.76 | 96.56 | 85.98 | 89.65 | 95.96 | 100.00 | Benznidazol | 4.48 | 90.88 | 88.11 | 92.45 | 94.28 | 100.00 |
| 9-Aminoacridine | 1.63 | 86.44 | 94.72 | 96.05 | 93.35 | 100.00 | Danofloxacin (mesylate) | 3.38 | 14.57 | 86.22 | 94.09 | 90.56 | 100.00 |
| Linezolid | 3.51 | 64.49 | 80.01 | 94.37 | 96.56 | 100.00 | Chlortetracycline (hydrochloride) | 45.01 | 95.42 | 97.93 | 99.62 | 99.02 | 100.00 |
| Mitomycin C | 2.50 | 3.56 | 4.90 | 4.12 | 39.67 | 100.00 | Erdafitinib | 50.32 | 80.59 | 88.27 | 90.96 | 92.79 | 100.00 |
| Ixazomib | 1.01 | 3.86 | 22.28 | 35.25 | 76.33 | 100.00 | Spiramycin | 55.32 | 80.28 | 85.48 | 90.26 | 91.77 | 100.00 |
| Oxytetracycline | 8.26 | 71.26 | 82.22 | 84.12 | 91.02 | 100.00 | Galanthamine | 15.07 | 83.33 | 88.40 | 86.83 | 89.85 | 100.00 |
| Morinidazole | 4.00 | 59.95 | 75.48 | 83.26 | 89.72 | 100.00 | Dacomitinib | 5.75 | 44.71 | 73.13 | 78.13 | 77.80 | 100.00 |
| Mupirocin | 5.95 | 79.96 | 95.35 | 95.15 | 97.98 | 100.00 | Sunitinib | 0.80 | 61.12 | 83.70 | 88.60 | 94.86 | 100.00 |
| Secnidazole | 4.40 | 82.78 | 89.01 | 94.66 | 96.43 | 100.00 | Pirarubicin (Hydrochloride) | 10.50 | 81.69 | 86.00 | 87.28 | 91.46 | 100.00 |
| Teniposide | 3.21 | 77.16 | 84.98 | 93.11 | 91.78 | 100.00 | Idarubicin (hydrochloride) | 3.85 | 7.74 | 83.45 | 80.06 | 88.67 | 100.00 |
| Daunorubicin (Hydrochloride) | 2.25 | 64.89 | 93.45 | 95.45 | 98.71 | 100.00 | Minocycline (hydrochloride) | 5.40 | 62.17 | 80.93 | 87.07 | 98.27 | 100.00 |
| Nitazoxanide | 3.40 | 4.45 | 45.63 | 79.26 | 79.46 | 100.00 | Doxycycline (hyclate) | 1.59 | 3.13 | 74.92 | 91.64 | 100.52 | 100.00 |
| Osimertinib | 5.60 | 83.75 | 86.39 | 89.05 | 98.78 | 100.00 | Dasatinib (hydrochloride) | 38.63 | 49.82 | 53.75 | 54.32 | 60.68 | 100.00 |
| (R)-(-)-Phenylephrine (hydrochloride) | 4.27 | 96.01 | 98.23 | 103.19 | 103.23 | 100.00 | Nifurtimox | 1.95 | 32.35 | 78.62 | 96.17 | 96.17 | 100.00 |
| Josamycin | 15.49 | 78.00 | 86.70 | 88.64 | 86.88 | 100.00 | Dantrolene (sodium hemiheptahydrate) | 29.15 | 82.27 | 90.54 | 102.88 | 101.64 | 100.00 |
| Methacycline (hydrochloride) | 3.07 | 4.98 | 57.31 | 68.21 | 75.44 | 100.00 | Artemotil | 0.51 | 85.72 | 90.68 | 96.23 | 95.46 | 100.00 |
| Imatinib (Mesylate) | 47.07 | 53.41 | 64.53 | 80.03 | 75.31 | 100.00 | Gramicidin | 0.41 | 93.53 | 94.19 | 99.27 | 107.53 | 100.00 |
| Ronidazole | 5.14 | 5.96 | 27.59 | 64.19 | 67.26 | 100.00 | Morinidazole (R enantiomer) | 2.86 | 10.13 | 89.69 | 90.71 | 98.08 | 100.00 |
| Nifuroxazide | 2.06 | 9.13 | 50.16 | 68.06 | 86.39 | 100.00 | Nadifloxacin | 1.03 | 100.33 | 100.73 | 101.54 | 103.98 | 100.00 |
| Tinidazole | 2.67 | 6.59 | 47.07 | 78.92 | 90.73 | 100.00 | Auranofin | 0.38 | 2.03 | 95.62 | 98.81 | 100.66 | 100.00 |
| Aclacinomycin A hydrochloride | 1.28 | 81.36 | 76.95 | 84.24 | 96.55 | 100.00 | Fidaxomicin | 41.48 | 96.04 | 99.52 | 100.01 | 102.68 | 100.00 |
| Nitrofurazone | 6.30 | 70.67 | 80.38 | 94.84 | 98.83 | 100.00 | Dextrorotation nimorazole phosphate ester | 35.28 | 85.31 | 97.40 | 97.64 | 99.79 | 100.00 |
| Epirubicin (hydrochloride) | 4.65 | 88.57 | 93.22 | 94.42 | 95.60 | 100.00 | Bosutinib | 10.14 | 69.90 | 87.30 | 90.72 | 97.19 | 100.00 |
| Mitoxantrone (dihydrochloride) | 31.96 | 87.29 | 89.03 | 88.48 | 95.77 | 100.00 | Metronidazole | 10.08 | 85.68 | 94.10 | 93.56 | 93.89 | 100.00 |
| Doxorubicin (hydrochloride) | 1.92 | 70.83 | 89.01 | 93.34 | 92.49 | 100.00 | Simeprevir | 38.77 | 96.19 | 97.25 | 98.50 | 100.83 | 100.00 |
| Tedizolid (phosphate) | 3.35 | 3.59 | 70.69 | 88.98 | 91.72 | 100.00 | Ribociclib | 30.94 | 95.99 | 97.22 | 99.48 | 101.40 | 100.00 |
| Domiphen (bromide) | 66.00 | 77.63 | 85.67 | 89.87 | 94.08 | 100.00 | Abemaciclib (methanesulfonate) | 47.24 | 89.88 | 93.89 | 93.33 | 94.22 | 100.00 |
| Bortezomib | 0.98 | 4.37 | 5.79 | 29.50 | 75.35 | 100.00 | Midostaurin | 34.30 | 95.75 | 96.03 | 97.48 | 98.65 | 100.00 |
| (S)-10-Hydroxycamptothecin | 85.08 | 89.87 | 84.98 | 93.73 | 98.23 | 100.00 | Clemastine (fumarate) | 41.07 | 97.09 | 98.29 | 99.30 | 99.73 | 100.00 |
| Dimetridazole | 3.61 | 70.96 | 88.81 | 95.72 | 97.79 | 100.00 | Octenidine (dihydrochloride) | 46.51 | 95.54 | 97.53 | 97.90 | 100.69 | 100.00 |
| Tamoxifen | 24.29 | 91.06 | 96.97 | 96.17 | 95.40 | 100.00 | Mepyramine maleate | 48.90 | 97.89 | 99.11 | 100.58 | 98.34 | 100.00 |
| Acetylspiramycin | 2.37 | 87.68 | 96.77 | 96.71 | 96.46 | 100.00 | Liothyronine | 50.62 | 92.45 | 93.33 | 93.46 | 97.42 | 100.00 |
| Gefitinib | 27.44 | 60.25 | 77.77 | 86.76 | 85.32 | 100.00 | Nintedanib | 36.24 | 95.16 | 97.54 | 98.13 | 98.32 | 100.00 |
| Ceritinib dihydrochloride | 39.86 | 86.82 | 92.06 | 88.46 | 86.07 | 100.00 | Imatinib | 41.03 | 95.70 | 96.94 | 98.42 | 98.99 | 100.00 |
| Delafloxacin (meglumine) | 1.54 | 2.08 | 73.78 | 85.63 | 84.75 | 100.00 |  |  |  |  |  |  |  |

**Table S2. Third drug screening of 25 hit compounds.**

| **Drug name** | **Invasion rates (%) of each drug’s multiple concentrations (μM)** | | | | | |
| --- | --- | --- | --- | --- | --- | --- |
|  | 10 | 1 | 0.1 | 0.01 | 0.01 | 0 |
| Morinidazole (R enantiomer) | 0.38 | 6.21 | 94.21 | 95.71 | 97.64 | 100.00 |
| Nifuratel | 1.46 | 3.12 | 7.32 | 59.85 | 93.93 | 100.00 |
| Auranofin | 0.76 | 2.34 | 88.21 | 93.68 | 97.35 | 100.00 |
| Mitomycin C | 0.24 | 1.49 | 2.22 | 41.57 | 93.18 | 100.00 |
| Ixazomib | 0.30 | 0.53 | 76.12 | 83.07 | 95.73 | 100.00 |
| Demeclocycline (hydrochloride) | 0.30 | 2.65 | 89.49 | 93.85 | 95.74 | 100.00 |
| Tinidazole | 2.87 | 8.88 | 90.15 | 89.23 | 88.24 | 100.00 |
| Danofloxacin (mesylate) | 2.06 | 77.40 | 87.17 | 93.23 | 98.11 | 100.00 |
| Nitazoxanide | 1.24 | 6.41 | 44.01 | 94.08 | 94.27 | 100.00 |
| Methacycline (hydrochloride) | 2.20 | 5.12 | 89.39 | 93.06 | 92.10 | 100.00 |
| Ronidazole | 2.89 | 3.06 | 5.74 | 81.81 | 87.52 | 100.00 |
| Nifuroxazide | 1.51 | 7.31 | 86.52 | 97.20 | 94.33 | 100.00 |
| Bortezomib | 9.72 | 35.28 | 66.11 | 84.28 | 92.79 | 100.00 |
| Delafloxacin (meglumine) | 0.81 | 1.96 | 82.73 | 93.02 | 86.37 | 100.00 |
| Retapamulin | 2.42 | 1.65 | 4.92 | 74.43 | 94.33 | 100.00 |
| Tigecycline (tetramesylate) | 1.12 | 2.36 | 88.10 | 89.06 | 89.80 | 100.00 |
| Furagin | 1.29 | 2.30 | 2.26 | 77.08 | 97.52 | 100.00 |
| Dasatinib | 0.90 | 71.67 | 83.39 | 95.07 | 97.67 | 100.00 |
| Ixazomib citrate | 0.40 | 54.25 | 91.44 | 100.03 | 96.71 | 100.00 |
| Tedizolid | 2.87 | 3.28 | 90.65 | 99.77 | 96.13 | 100.00 |
| Idarubicin (hydrochloride) | 1.34 | 1.25 | 100.06 | 95.32 | 98.46 | 100.00 |
| Doxycycline (hyclate) | 1.58 | 5.76 | 93.64 | 101.94 | 98.24 | 100.00 |
| Dasatinib (hydrochloride) | 56.83 | 87.73 | 92.00 | 99.47 | 101.09 | 100.00 |
| Dacomitinib | 53.94 | 90.66 | 98.59 | 98.86 | 101.52 | 100.00 |
| Nifurtimox | 0.53 | 5.05 | 93.55 | 96.97 | 97.47 | 100.00 |

**Table S3. Differential genes affected by NTZ in RNA-Seq.**

| **genes** | **logFC** | **logCPM** | **LR** | **PValue** | **FDR** | **Change (KQ vs KR)** | **genes** | **logFC** | **logCPM** | **LR** | **PValue** | **FDR** | **Change (KQ-Control vs KQ-NTZ)** |
| --- | --- | --- | --- | --- | --- | --- | --- | --- | --- | --- | --- | --- | --- |
| CALB1 | -5.7817 | 4.11453 | 587.423 | 9.11E-130 | 8.77E-127 | DOWN | CALB1 | 1.0592 | 0.01703 | 16.9757 | 3.79E-05 | 0.0001584 | UP |
| SLFN11 | -3.9676 | 3.55879 | 493.347 | 2.66E-109 | 1.79E-106 | DOWN | SLFN11 | 0.72659 | 0.94588 | 12.2295 | 0.0004704 | 0.0015512 | UP |
| UGT2B7 | -4.5113 | 2.01556 | 401.832 | 2.20E-89 | 1.14E-86 | DOWN | UGT2B7 | 1.60778 | -0.4336 | 27.0066 | 2.03E-07 | 1.29E-06 | UP |
| FXYD3 | -2.5366 | 3.45588 | 271.108 | 6.51E-61 | 1.49E-58 | DOWN | FXYD3 | 0.8317 | 2.17042 | 37.2699 | 1.03E-09 | 9.40E-09 | UP |
| AKR1C3 | -2.9021 | 2.33691 | 252.573 | 7.14E-57 | 1.35E-54 | DOWN | AKR1C3 | 1.07812 | 0.91791 | 23.9408 | 9.93E-07 | 5.57E-06 | UP |
| SYN3 | -1.6104 | 6.33322 | 242.275 | 1.26E-54 | 2.09E-52 | DOWN | SYN3 | 1.22714 | 6.04795 | 266.738 | 5.83E-60 | 1.58E-57 | UP |
| PKIA | -2.2695 | 4.14273 | 241.564 | 1.79E-54 | 2.88E-52 | DOWN | PKIA | 0.85394 | 3.08888 | 66.2632 | 3.95E-16 | 7.74E-15 | UP |
| KRT17 | -2.8286 | 4.53091 | 237.29 | 1.53E-53 | 2.40E-51 | DOWN | KRT17 | 1.07552 | 3.14709 | 106.634 | 5.35E-25 | 2.31E-23 | UP |
| PLEKHA7 | -2.0155 | 4.96364 | 234.334 | 6.77E-53 | 1.01E-50 | DOWN | PLEKHA7 | 0.69958 | 4.01625 | 51.8209 | 6.08E-13 | 8.51E-12 | UP |
| LRRK1 | -1.4591 | 8.0821 | 213.425 | 2.46E-48 | 2.83E-46 | DOWN | LRRK1 | 0.87148 | 7.66896 | 137.368 | 1.00E-31 | 6.88E-30 | UP |
| NT5E | -1.8565 | 6.03804 | 201.707 | 8.86E-46 | 9.40E-44 | DOWN | NT5E | 0.59635 | 5.15197 | 45.6231 | 1.43E-11 | 1.69E-10 | UP |
| TRIM29 | -2.7245 | 1.7376 | 176.451 | 2.89E-40 | 2.32E-38 | DOWN | TRIM29 | 0.96934 | 0.41449 | 18.9486 | 1.34E-05 | 6.14E-05 | UP |
| WNT9A | -1.8891 | 4.44044 | 165.959 | 5.65E-38 | 3.99E-36 | DOWN | WNT9A | 1.03947 | 3.81277 | 105.481 | 9.58E-25 | 4.03E-23 | UP |
| TRIM31 | -3.5691 | 0.59401 | 158.847 | 2.02E-36 | 1.35E-34 | DOWN | TRIM31 | 1.20684 | -1.1912 | 8.91759 | 0.0028244 | 0.0076117 | UP |
| ADIRF | -2.8561 | 2.91766 | 130.364 | 3.41E-30 | 1.74E-28 | DOWN | ADIRF | 0.62071 | 1.24409 | 9.19763 | 0.0024233 | 0.0066513 | UP |
| KIAA0319 | -2.1882 | 1.42896 | 125.761 | 3.47E-29 | 1.69E-27 | DOWN | KIAA0319 | 2.32233 | 1.5263 | 151.935 | 6.55E-35 | 5.64E-33 | UP |
| CCNA1 | -1.4932 | 3.51808 | 111.846 | 3.86E-26 | 1.62E-24 | DOWN | CCNA1 | 0.9046 | 3.10354 | 61.9192 | 3.58E-15 | 6.37E-14 | UP |
| METRNL | -1.9667 | 4.56838 | 110.778 | 6.62E-26 | 2.72E-24 | DOWN | METRNL | 0.65235 | 3.63404 | 25.6567 | 4.08E-07 | 2.44E-06 | UP |
| FA2H | -1.7761 | 4.03734 | 106.544 | 5.60E-25 | 2.18E-23 | DOWN | FA2H | 1.57429 | 3.87693 | 158.841 | 2.03E-36 | 1.99E-34 | UP |
| TMPRSS2 | -1.9831 | 1.72888 | 100.393 | 1.25E-23 | 4.55E-22 | DOWN | TMPRSS2 | 1.20469 | 1.15231 | 45.2092 | 1.77E-11 | 2.05E-10 | UP |
| SDCBP2 | -1.837 | 2.25404 | 95.3228 | 1.62E-22 | 5.41E-21 | DOWN | SDCBP2 | 1.23764 | 1.8085 | 56.2693 | 6.32E-14 | 9.88E-13 | UP |
| GALNT12 | -1.2242 | 4.8041 | 89.9938 | 2.39E-21 | 7.24E-20 | DOWN | GALNT12 | 0.88616 | 4.56914 | 85.9346 | 1.86E-20 | 5.56E-19 | UP |
| IL32 | -1.6084 | 3.7749 | 89.0236 | 3.90E-21 | 1.17E-19 | DOWN | IL32 | 1.09893 | 3.40443 | 76.1653 | 2.61E-18 | 6.28E-17 | UP |
| BICDL1 | -1.9474 | 1.39424 | 88.8661 | 4.22E-21 | 1.26E-19 | DOWN | BICDL1 | 0.70503 | 0.53612 | 9.51857 | 0.002034 | 0.0056902 | UP |
| STK32A | -2.0428 | 1.81659 | 83.0599 | 7.96E-20 | 2.17E-18 | DOWN | STK32A | 1.27433 | 1.23797 | 41.9716 | 9.26E-11 | 9.83E-10 | UP |
| UST | -1.2488 | 2.97384 | 78.817 | 6.81E-19 | 1.72E-17 | DOWN | UST | 0.682 | 2.59579 | 31.2068 | 2.32E-08 | 1.71E-07 | UP |
| S100A6 | -1.1896 | 9.31551 | 75.4508 | 3.75E-18 | 8.86E-17 | DOWN | S100A6 | 0.64267 | 8.9528 | 62.4163 | 2.78E-15 | 4.98E-14 | UP |
| IDO1 | -1.273 | 2.4039 | 74.0283 | 7.70E-18 | 1.77E-16 | DOWN | IDO1 | 0.67206 | 2.00492 | 23.51 | 1.24E-06 | 6.85E-06 | UP |
| FUT3 | -1.1973 | 5.3804 | 70.2413 | 5.25E-17 | 1.13E-15 | DOWN | FUT3 | 0.76258 | 5.08609 | 85.3278 | 2.53E-20 | 7.41E-19 | UP |
| AKR1C1 | -1.6991 | 1.79013 | 68.9282 | 1.02E-16 | 2.13E-15 | DOWN | AKR1C1 | 1.37043 | 1.54342 | 55.9829 | 7.31E-14 | 1.13E-12 | UP |
| COL4A3 | -2.6402 | 0.0786 | 67.2436 | 2.40E-16 | 4.87E-15 | DOWN | COL4A3 | 2.25798 | -0.2316 | 53.3257 | 2.83E-13 | 4.10E-12 | UP |
| PLEKHG1 | -1.3377 | 2.85111 | 65.397 | 6.12E-16 | 1.20E-14 | DOWN | PLEKHG1 | 0.67109 | 2.40322 | 21.9136 | 2.85E-06 | 1.48E-05 | UP |
| BDH1 | -1.2803 | 3.33056 | 64.5864 | 9.24E-16 | 1.79E-14 | DOWN | BDH1 | 0.73092 | 2.9599 | 37.2682 | 1.03E-09 | 9.40E-09 | UP |
| KDELR2 | -0.797 | 8.13454 | 63.7194 | 1.43E-15 | 2.74E-14 | DOWN | KDELR2 | 0.62104 | 8.01934 | 86.8904 | 1.15E-20 | 3.51E-19 | UP |
| COL4A4 | -1.5734 | 1.25366 | 60.4969 | 7.37E-15 | 1.31E-13 | DOWN | COL4A4 | 0.81286 | 0.73643 | 15.4802 | 8.34E-05 | 0.0003259 | UP |
| SMCO4 | -1.3094 | 2.02321 | 60.1764 | 8.67E-15 | 1.53E-13 | DOWN | SMCO4 | 0.66645 | 1.59798 | 17.0065 | 3.73E-05 | 0.0001561 | UP |
| NAV1 | -1.0097 | 3.50475 | 58.2838 | 2.27E-14 | 3.89E-13 | DOWN | NAV1 | 1.22605 | 3.64348 | 149.967 | 1.76E-34 | 1.46E-32 | UP |
| FER1L6 | -1.1069 | 3.47598 | 58.1746 | 2.40E-14 | 4.09E-13 | DOWN | FER1L6 | 0.62225 | 3.15749 | 31.0504 | 2.51E-08 | 1.84E-07 | UP |
| SLC41A2 | -0.9735 | 4.00807 | 54.336 | 1.69E-13 | 2.65E-12 | DOWN | SLC41A2 | 0.84442 | 3.91625 | 83.9291 | 5.13E-20 | 1.45E-18 | UP |
| CSF1R | -1.5874 | 0.62721 | 50.1373 | 1.43E-12 | 2.05E-11 | DOWN | CSF1R | 0.94493 | 0.19089 | 12.9263 | 0.000324 | 0.0011053 | UP |
| SAT1 | -0.6712 | 8.14908 | 48.0777 | 4.10E-12 | 5.59E-11 | DOWN | SAT1 | 0.79515 | 8.21984 | 165.052 | 8.91E-38 | 9.73E-36 | UP |
| IL1RN | -1.2843 | 1.79723 | 47.8359 | 4.63E-12 | 6.30E-11 | DOWN | IL1RN | 1.09077 | 1.65803 | 41.5479 | 1.15E-10 | 1.21E-09 | UP |
| SLC44A4 | -0.9725 | 3.25236 | 47.8268 | 4.66E-12 | 6.32E-11 | DOWN | SLC44A4 | 1.04233 | 3.29096 | 84.5001 | 3.84E-20 | 1.10E-18 | UP |
| PRKAR1B | -0.9855 | 4.41543 | 47.2839 | 6.14E-12 | 8.27E-11 | DOWN | PRKAR1B | 0.80079 | 4.28902 | 65.2224 | 6.69E-16 | 1.28E-14 | UP |
| IL36RN | -1.0671 | 1.90503 | 43.1046 | 5.19E-11 | 6.39E-10 | DOWN | IL36RN | 0.63984 | 1.62866 | 17.8445 | 2.40E-05 | 0.0001047 | UP |
| BTN3A3 | -0.9282 | 3.30005 | 42.7971 | 6.07E-11 | 7.45E-10 | DOWN | BTN3A3 | 0.66967 | 3.13031 | 29.508 | 5.57E-08 | 3.86E-07 | UP |
| PDGFA | -0.8889 | 3.45118 | 41.71 | 1.06E-10 | 1.26E-09 | DOWN | PDGFA | 0.76219 | 3.36387 | 46.6161 | 8.63E-12 | 1.05E-10 | UP |
| FIBCD1 | -1.1918 | 1.32428 | 40.6463 | 1.82E-10 | 2.13E-09 | DOWN | FIBCD1 | 1.27951 | 1.37502 | 54.3879 | 1.65E-13 | 2.44E-12 | UP |
| NR4A2 | -1.9173 | -0.025 | 39.2337 | 3.76E-10 | 4.23E-09 | DOWN | NR4A2 | 0.95672 | -0.6685 | 8.32064 | 0.0039197 | 0.0101533 | UP |
| DMPK | -0.8398 | 4.74491 | 38.3009 | 6.06E-10 | 6.64E-09 | DOWN | DMPK | 0.63193 | 4.60892 | 51.2281 | 8.22E-13 | 1.13E-11 | UP |
| NECTIN4 | -0.7594 | 3.79637 | 38.2158 | 6.33E-10 | 6.92E-09 | DOWN | NECTIN4 | 1.85177 | 4.56028 | 440.888 | 6.94E-98 | 6.26E-95 | UP |
| IGDCC4 | -1.5015 | 0.75851 | 36.1432 | 1.83E-09 | 1.89E-08 | DOWN | IGDCC4 | 1.49264 | 0.74243 | 50.2495 | 1.35E-12 | 1.81E-11 | UP |
| GCNT3 | -1.6004 | 0.05196 | 34.1076 | 5.21E-09 | 5.06E-08 | DOWN | GCNT3 | 2.70123 | 0.89105 | 90.7257 | 1.65E-21 | 5.46E-20 | UP |
| TJP3 | -1.0151 | 3.84179 | 34.0617 | 5.34E-09 | 5.17E-08 | DOWN | TJP3 | 0.61337 | 3.58119 | 21.0407 | 4.50E-06 | 2.25E-05 | UP |
| ESPN | -1.0902 | 3.34943 | 33.9526 | 5.65E-09 | 5.46E-08 | DOWN | ESPN | 0.66884 | 3.07298 | 26.3549 | 2.84E-07 | 1.75E-06 | UP |
| MVP | -0.9004 | 4.66619 | 33.3039 | 7.88E-09 | 7.44E-08 | DOWN | MVP | 0.77777 | 4.58166 | 61.109 | 5.40E-15 | 9.51E-14 | UP |
| FRY | -1.1443 | 1.85165 | 33.0531 | 8.97E-09 | 8.38E-08 | DOWN | FRY | 1.20434 | 1.88269 | 39.1959 | 3.83E-10 | 3.74E-09 | UP |
| CYP1B1 | -0.9046 | 7.52369 | 32.7438 | 1.05E-08 | 9.74E-08 | DOWN | CYP1B1 | 0.7612 | 7.42326 | 54.7477 | 1.37E-13 | 2.06E-12 | UP |
| APOL2 | -0.8281 | 4.08117 | 31.3216 | 2.19E-08 | 1.92E-07 | DOWN | APOL2 | 0.94455 | 4.14902 | 106.222 | 6.59E-25 | 2.81E-23 | UP |
| TMOD1 | -1.1327 | 1.40827 | 30.0571 | 4.20E-08 | 3.54E-07 | DOWN | TMOD1 | 1.79284 | 1.87155 | 89.0785 | 3.79E-21 | 1.20E-19 | UP |
| TTC9 | -0.9213 | 1.95543 | 29.5388 | 5.48E-08 | 4.54E-07 | DOWN | TTC9 | 0.75028 | 1.84028 | 23.7468 | 1.10E-06 | 6.13E-06 | UP |
| FTH1 | -0.7088 | 11.7871 | 29.4943 | 5.61E-08 | 4.64E-07 | DOWN | FTH1 | 1.21801 | 12.1182 | 207.422 | 5.02E-47 | 8.28E-45 | UP |
| TMEM140 | -1.4704 | 2.14081 | 27.4004 | 1.65E-07 | 1.28E-06 | DOWN | TMEM140 | 0.99929 | 1.80945 | 31.0603 | 2.50E-08 | 1.83E-07 | UP |
| PDZK1 | -1.5666 | 0.06328 | 27.3744 | 1.68E-07 | 1.30E-06 | DOWN | PDZK1 | 0.85791 | -0.3983 | 7.80207 | 0.0052186 | 0.0130293 | UP |
| WHRN | -0.6879 | 3.85099 | 27.28 | 1.76E-07 | 1.36E-06 | DOWN | WHRN | 0.86899 | 3.95681 | 86.7058 | 1.26E-20 | 3.81E-19 | UP |
| AKR1B1 | -0.9354 | 2.03462 | 27.0879 | 1.94E-07 | 1.49E-06 | DOWN | AKR1B1 | 1.23699 | 2.22769 | 65.7296 | 5.17E-16 | 1.01E-14 | UP |
| HID1 | -0.7332 | 5.56772 | 27.012 | 2.02E-07 | 1.54E-06 | DOWN | HID1 | 1.00495 | 5.7361 | 129.017 | 6.72E-30 | 4.16E-28 | UP |
| PTPRR | -0.7171 | 3.31989 | 26.8099 | 2.24E-07 | 1.70E-06 | DOWN | PTPRR | 1.06992 | 3.53901 | 81.4431 | 1.80E-19 | 4.90E-18 | UP |
| MUC4 | -1.3098 | 1.2727 | 26.2662 | 2.97E-07 | 2.22E-06 | DOWN | MUC4 | 1.57495 | 1.44885 | 44.8541 | 2.12E-11 | 2.43E-10 | UP |
| RND1 | -0.8298 | 2.66849 | 25.5341 | 4.35E-07 | 3.19E-06 | DOWN | RND1 | 0.69157 | 2.5751 | 30.0615 | 4.19E-08 | 2.96E-07 | UP |
| PITPNM3 | -0.7813 | 3.20015 | 24.9673 | 5.83E-07 | 4.20E-06 | DOWN | PITPNM3 | 0.97064 | 3.31368 | 55.1073 | 1.14E-13 | 1.72E-12 | UP |
| SERPINF2 | -0.9734 | 1.98847 | 23.879 | 1.03E-06 | 7.09E-06 | DOWN | SERPINF2 | 0.74586 | 1.8379 | 21.9083 | 2.86E-06 | 1.48E-05 | UP |
| NLRC5 | -0.649 | 3.48083 | 23.7816 | 1.08E-06 | 7.42E-06 | DOWN | NLRC5 | 0.85083 | 3.59865 | 71.2983 | 3.07E-17 | 6.75E-16 | UP |
| ANXA8 | -1.2186 | 0.13659 | 22.6771 | 1.92E-06 | 1.27E-05 | DOWN | ANXA8 | 0.79061 | -0.136 | 6.88633 | 0.0086857 | 0.0203424 | UP |
| ANG | -0.8473 | 1.86206 | 22.1724 | 2.49E-06 | 1.63E-05 | DOWN | ANG | 1.35392 | 2.19154 | 97.1161 | 6.54E-23 | 2.38E-21 | UP |
| ARG2 | -0.7328 | 2.27031 | 21.763 | 3.08E-06 | 1.98E-05 | DOWN | ARG2 | 2.13821 | 3.27055 | 304.926 | 2.78E-68 | 1.14E-65 | UP |
| PROS1 | -0.8518 | 3.66935 | 21.5461 | 3.45E-06 | 2.19E-05 | DOWN | PROS1 | 0.91337 | 3.69947 | 60.3784 | 7.83E-15 | 1.35E-13 | UP |
| TNFSF14 | -0.9869 | 1.18352 | 21.4762 | 3.58E-06 | 2.27E-05 | DOWN | TNFSF14 | 1.64287 | 1.62729 | 99.4115 | 2.05E-23 | 7.71E-22 | UP |
| CYSTM1 | -0.6035 | 5.31644 | 21.24 | 4.05E-06 | 2.55E-05 | DOWN | CYSTM1 | 0.62464 | 5.32289 | 56.6076 | 5.32E-14 | 8.40E-13 | UP |
| MAP2 | -0.7239 | 2.36435 | 20.6018 | 5.65E-06 | 3.46E-05 | DOWN | MAP2 | 0.89041 | 2.46024 | 48.5515 | 3.22E-12 | 4.12E-11 | UP |
| CFB | -0.6245 | 4.09823 | 20.3873 | 6.32E-06 | 3.83E-05 | DOWN | CFB | 0.92205 | 4.27822 | 94.4326 | 2.54E-22 | 8.94E-21 | UP |
| SERINC5 | -0.6781 | 3.93237 | 20.1114 | 7.31E-06 | 4.37E-05 | DOWN | SERINC5 | 0.71509 | 3.9462 | 40.7333 | 1.74E-10 | 1.78E-09 | UP |
| MFSD2A | -0.6263 | 3.20739 | 19.9473 | 7.96E-06 | 4.74E-05 | DOWN | MFSD2A | 0.61519 | 3.19327 | 35.5355 | 2.50E-09 | 2.18E-08 | UP |
| SLC1A1 | -0.8043 | 1.43849 | 19.7309 | 8.91E-06 | 5.27E-05 | DOWN | SLC1A1 | 0.67722 | 1.35319 | 15.9665 | 6.45E-05 | 0.0002577 | UP |
| CHPF2 | -0.6164 | 4.78749 | 19.5936 | 9.58E-06 | 5.62E-05 | DOWN | CHPF2 | 0.72636 | 4.84872 | 59.6491 | 1.13E-14 | 1.93E-13 | UP |
| MEF2C | -1.1228 | 0.86593 | 18.8688 | 1.40E-05 | 7.99E-05 | DOWN | MEF2C | 0.66317 | 0.57207 | 5.48274 | 0.0192052 | 0.040527 | UP |
| SRPX | -0.5894 | 4.22424 | 17.2047 | 3.36E-05 | 0.000179 | DOWN | SRPX | 0.59828 | 4.22307 | 31.4226 | 2.08E-08 | 1.54E-07 | UP |
| TMEM269 | -1.2669 | -0.3912 | 16.7319 | 4.31E-05 | 0.000224 | DOWN | TMEM269 | 0.77153 | -0.695 | 5.48635 | 0.0191655 | 0.0404622 | UP |
| RMDN2 | -0.928 | 2.09612 | 16.1809 | 5.76E-05 | 0.000294 | DOWN | RMDN2 | 0.9051 | 2.07113 | 28.0296 | 1.19E-07 | 7.89E-07 | UP |
| PDGFRL | -0.7474 | 3.20049 | 15.8003 | 7.04E-05 | 0.000352 | DOWN | PDGFRL | 0.7023 | 3.16743 | 19.562 | 9.74E-06 | 4.58E-05 | UP |
| ABAT | -0.843 | 0.68841 | 15.7366 | 7.28E-05 | 0.000363 | DOWN | ABAT | 1.04925 | 0.80964 | 26.9317 | 2.11E-07 | 1.34E-06 | UP |
| CAPN12 | -1.1654 | 1.33952 | 14.7649 | 0.000122 | 0.000581 | DOWN | CAPN12 | 0.76697 | 1.07809 | 9.00496 | 0.0026925 | 0.0073013 | UP |
| KLHDC7B | -0.7236 | 2.83689 | 14.1818 | 0.000166 | 0.000766 | DOWN | KLHDC7B | 1.28578 | 3.19913 | 103.8 | 2.24E-24 | 9.12E-23 | UP |
| IFITM10 | -0.7416 | 1.82972 | 13.9182 | 0.000191 | 0.000872 | DOWN | IFITM10 | 0.9719 | 1.96704 | 29.3413 | 6.07E-08 | 4.19E-07 | UP |
| MINAR1 | -0.8657 | 0.68767 | 13.5918 | 0.000227 | 0.001019 | DOWN | MINAR1 | 1.16478 | 0.86951 | 30.0306 | 4.25E-08 | 3.00E-07 | UP |
| SPOCK2 | -1.0489 | -0.092 | 13.5627 | 0.000231 | 0.001034 | DOWN | SPOCK2 | 1.75647 | 0.3769 | 44.7711 | 2.21E-11 | 2.53E-10 | UP |
| SGK3 | -1.3021 | 2.37509 | 13.4521 | 0.000245 | 0.001091 | DOWN | SGK3 | 1.02624 | 2.17502 | 10.7442 | 0.001046 | 0.0031686 | UP |
| CYP8B1 | -1.5848 | -1.17 | 13.3562 | 0.000258 | 0.001142 | DOWN | CYP8B1 | 1.23785 | -1.3867 | 8.18051 | 0.0042343 | 0.0108396 | UP |
| DISP1 | -0.784 | 1.09608 | 13.161 | 0.000286 | 0.001255 | DOWN | DISP1 | 0.63462 | 0.9996 | 9.81632 | 0.0017297 | 0.0049451 | UP |
| HSPB8 | -0.6805 | 1.59359 | 12.9147 | 0.000326 | 0.001411 | DOWN | HSPB8 | 1.78363 | 2.3405 | 162.214 | 3.71E-37 | 3.78E-35 | UP |
| ARSK | -0.655 | 2.13905 | 12.7702 | 0.000352 | 0.001513 | DOWN | ARSK | 0.76535 | 2.19766 | 31.4464 | 2.05E-08 | 1.53E-07 | UP |
| GDPD3 | -0.9526 | 0.26863 | 11.8368 | 0.000581 | 0.002372 | DOWN | GDPD3 | 1.10852 | 0.36028 | 16.1527 | 5.84E-05 | 0.0002358 | UP |
| TBX2 | -0.6302 | 2.60567 | 11.5149 | 0.00069 | 0.002751 | DOWN | TBX2 | 0.60552 | 2.58387 | 22.6601 | 1.93E-06 | 1.03E-05 | UP |
| FAM178B | -1.4187 | -1.273 | 10.7883 | 0.001021 | 0.003876 | DOWN | FAM178B | 1.10987 | -1.4583 | 6.30435 | 0.0120442 | 0.0271647 | UP |
| WNT4 | -0.6657 | 2.33764 | 10.0805 | 0.001498 | 0.005428 | DOWN | WNT4 | 1.78014 | 3.09904 | 134.621 | 4.00E-31 | 2.64E-29 | UP |
| STARD4 | -0.655 | 2.94779 | 9.9361 | 0.001621 | 0.005825 | DOWN | STARD4 | 1.0605 | 3.19615 | 56.6344 | 5.25E-14 | 8.31E-13 | UP |
| ATP2A3 | -1.3354 | -0.9811 | 9.55234 | 0.001997 | 0.006973 | DOWN | ATP2A3 | 1.28397 | -1.019 | 10.7505 | 0.0010425 | 0.0031586 | UP |
| RORC | -0.8214 | 0.29146 | 8.36437 | 0.003826 | 0.012326 | DOWN | RORC | 0.98858 | 0.38767 | 15.1333 | 0.0001002 | 0.0003842 | UP |
| CGB5 | -1.1244 | -1.0712 | 7.61188 | 0.005799 | 0.017586 | DOWN | CGB5 | 1.07349 | -1.1085 | 7.23402 | 0.0071535 | 0.017152 | UP |
| ANKRD37 | -0.6173 | 0.69908 | 7.28911 | 0.006937 | 0.020451 | DOWN | ANKRD37 | 0.91253 | 0.86845 | 21.1158 | 4.32E-06 | 2.17E-05 | UP |
| ASDURF | -0.6393 | 1.51428 | 6.48196 | 0.010897 | 0.030128 | DOWN | ASDURF | 0.651 | 1.51161 | 13.7148 | 0.0002128 | 0.0007564 | UP |
| LOC105374299 | -0.8134 | -0.6254 | 6.27438 | 0.01225 | 0.033347 | DOWN | LOC105374299 | 1.73222 | -0.0456 | 38.3214 | 6.00E-10 | 5.66E-09 | UP |
| CYP2E1 | -0.9362 | -0.1304 | 5.99442 | 0.014351 | 0.038146 | DOWN | CYP2E1 | 0.96908 | -0.1216 | 9.03625 | 0.0026468 | 0.0071874 | UP |
| ARX | -0.6759 | -0.2842 | 5.64273 | 0.017528 | 0.045196 | DOWN | ARX | 0.86879 | -0.1809 | 10.3305 | 0.0013085 | 0.0038632 | UP |
| APLN | 3.47409 | 5.34326 | 514.219 | 7.66E-114 | 6.07E-111 | UP | APLN | -0.8669 | 5.84334 | 78.2039 | 9.29E-19 | 2.38E-17 | DOWN |
| MMP9 | 3.15427 | 3.17793 | 366.34 | 1.17E-81 | 4.39E-79 | UP | MMP9 | -0.7252 | 3.69656 | 45.6627 | 1.40E-11 | 1.66E-10 | DOWN |
| TCN1 | 5.29417 | 0.94527 | 265.544 | 1.06E-59 | 2.27E-57 | UP | TCN1 | -0.7528 | 1.55238 | 18.2693 | 1.92E-05 | 8.53E-05 | DOWN |
| LY6K | 2.65908 | 2.67481 | 235.817 | 3.21E-53 | 4.92E-51 | UP | LY6K | -0.6327 | 3.16842 | 37.214 | 1.06E-09 | 9.62E-09 | DOWN |
| FLI1 | 3.71519 | 1.26222 | 233.538 | 1.01E-52 | 1.48E-50 | UP | FLI1 | -0.8219 | 1.78129 | 28.6047 | 8.88E-08 | 5.97E-07 | DOWN |
| MAP7D3 | 2.211 | 3.03164 | 223.835 | 1.32E-50 | 1.72E-48 | UP | MAP7D3 | -0.8315 | 3.38376 | 70.9126 | 3.73E-17 | 8.14E-16 | DOWN |
| MSMP | 2.17232 | 4.75156 | 207.712 | 4.34E-47 | 4.79E-45 | UP | MSMP | -1.1495 | 4.99306 | 156.538 | 6.46E-36 | 6.16E-34 | DOWN |
| ASB2 | 2.29573 | 2.83443 | 200.823 | 1.38E-45 | 1.44E-43 | UP | ASB2 | -1.5518 | 2.98381 | 179.889 | 5.12E-41 | 6.54E-39 | DOWN |
| CSF3 | 1.7326 | 8.49005 | 185.198 | 3.55E-42 | 3.19E-40 | UP | CSF3 | -0.9191 | 8.717 | 160.366 | 9.41E-37 | 9.51E-35 | DOWN |
| FAM43A | 2.25728 | 4.02677 | 178.828 | 8.74E-41 | 7.27E-39 | UP | FAM43A | -1.1246 | 4.29174 | 89.7395 | 2.72E-21 | 8.80E-20 | DOWN |
| LTB | 2.28191 | 3.59719 | 176.617 | 2.66E-40 | 2.14E-38 | UP | LTB | -0.7686 | 3.986 | 45.3672 | 1.63E-11 | 1.91E-10 | DOWN |
| SERPINA3 | 2.14737 | 2.65194 | 173.695 | 1.15E-39 | 8.94E-38 | UP | SERPINA3 | -0.7667 | 3.01528 | 43.5209 | 4.19E-11 | 4.64E-10 | DOWN |
| INHBB | 1.43086 | 7.47732 | 167.99 | 2.03E-38 | 1.47E-36 | UP | INHBB | -0.6733 | 7.71831 | 107.683 | 3.15E-25 | 1.41E-23 | DOWN |
| BDKRB2 | 1.98076 | 2.46247 | 155.061 | 1.36E-35 | 8.80E-34 | UP | BDKRB2 | -1.3479 | 2.60795 | 116.459 | 3.77E-27 | 1.96E-25 | DOWN |
| AADACL3 | 8.42918 | -0.1931 | 148.692 | 3.35E-34 | 2.08E-32 | UP | AADACL3 | -1.5511 | 0.18888 | 34.7231 | 3.80E-09 | 3.23E-08 | DOWN |
| LOC105373989 | 1.83218 | 4.38942 | 144.732 | 2.46E-33 | 1.46E-31 | UP | LOC105373989 | -0.7405 | 4.70216 | 54.4465 | 1.60E-13 | 2.38E-12 | DOWN |
| GBX2 | 6.05451 | 0.09996 | 137.228 | 1.08E-31 | 5.82E-30 | UP | GBX2 | -0.9793 | 0.62953 | 16.7422 | 4.28E-05 | 0.0001769 | DOWN |
| RPP25 | 2.1232 | 2.82525 | 132.694 | 1.06E-30 | 5.49E-29 | UP | RPP25 | -0.6095 | 3.24366 | 32.2211 | 1.38E-08 | 1.05E-07 | DOWN |
| TENM4 | 1.83059 | 3.0486 | 127.201 | 1.68E-29 | 8.29E-28 | UP | TENM4 | -0.7441 | 3.35722 | 35.2133 | 2.95E-09 | 2.55E-08 | DOWN |
| SLITRK5 | 1.92195 | 1.85046 | 114.391 | 1.07E-26 | 4.56E-25 | UP | SLITRK5 | -0.708 | 2.18836 | 22.7534 | 1.84E-06 | 9.90E-06 | DOWN |
| BRINP2 | 1.36951 | 4.10086 | 108.069 | 2.60E-25 | 1.03E-23 | UP | BRINP2 | -0.6846 | 4.32003 | 49.7712 | 1.73E-12 | 2.29E-11 | DOWN |
| HSD3B1 | 2.1799 | 1.10758 | 101.54 | 7.00E-24 | 2.61E-22 | UP | HSD3B1 | -0.7148 | 1.48723 | 17.7904 | 2.47E-05 | 0.0001073 | DOWN |
| FLRT1 | 1.96874 | 2.83762 | 100.691 | 1.08E-23 | 3.96E-22 | UP | FLRT1 | -0.8174 | 3.14821 | 44.2996 | 2.82E-11 | 3.18E-10 | DOWN |
| NRK | 2.86809 | 2.41951 | 98.1291 | 3.92E-23 | 1.37E-21 | UP | NRK | -1.1538 | 2.75781 | 38.2002 | 6.38E-10 | 6.00E-09 | DOWN |
| MYBL2 | 1.25309 | 8.83486 | 92.4115 | 7.04E-22 | 2.21E-20 | UP | MYBL2 | -0.7249 | 9.0063 | 107.446 | 3.55E-25 | 1.58E-23 | DOWN |
| ZFPM2 | 6.02655 | -0.8051 | 91.2233 | 1.28E-21 | 3.98E-20 | UP | ZFPM2 | -1.4661 | -0.4351 | 22.3882 | 2.23E-06 | 1.18E-05 | DOWN |
| SHISA3 | 1.78626 | 1.69814 | 90.6414 | 1.72E-21 | 5.28E-20 | UP | SHISA3 | -0.8421 | 1.95864 | 32.21 | 1.38E-08 | 1.06E-07 | DOWN |
| NKD1 | 3.44728 | 0.03028 | 86.7498 | 1.23E-20 | 3.53E-19 | UP | NKD1 | -0.7476 | 0.53693 | 9.43199 | 0.0021323 | 0.0059235 | DOWN |
| TNFRSF11B | 1.1669 | 5.46874 | 75.9963 | 2.84E-18 | 6.78E-17 | UP | TNFRSF11B | -2.1243 | 5.22974 | 491.175 | 7.91E-109 | 8.92E-106 | DOWN |
| IGSF23 | 1.98216 | 0.88033 | 75.3598 | 3.92E-18 | 9.23E-17 | UP | IGSF23 | -0.7201 | 1.21996 | 17.9242 | 2.30E-05 | 0.0001008 | DOWN |
| CXCL5 | 1.33245 | 3.02175 | 75.284 | 4.08E-18 | 9.57E-17 | UP | CXCL5 | -1.0464 | 3.10335 | 72.0472 | 2.10E-17 | 4.68E-16 | DOWN |
| SGK1 | 0.95135 | 6.01618 | 73.3189 | 1.10E-17 | 2.52E-16 | UP | SGK1 | -0.7813 | 6.06999 | 88.7277 | 4.53E-21 | 1.42E-19 | DOWN |
| PRRT4 | 2.82183 | -0.3309 | 62.9502 | 2.12E-15 | 3.98E-14 | UP | PRRT4 | -0.6032 | 0.15824 | 6.19375 | 0.0128202 | 0.028676 | DOWN |
| CD14 | 1.96782 | 0.57545 | 62.3404 | 2.89E-15 | 5.36E-14 | UP | CD14 | -2.3874 | 0.50247 | 89.3882 | 3.24E-21 | 1.04E-19 | DOWN |
| TNF | 1.48546 | 1.62886 | 62.1087 | 3.25E-15 | 6.00E-14 | UP | TNF | -0.6786 | 1.87647 | 13.7556 | 0.0002082 | 0.0007413 | DOWN |
| FOXI1 | 5.25728 | -1.2971 | 57.3082 | 3.73E-14 | 6.25E-13 | UP | FOXI1 | -1.9605 | -1.0565 | 22.6207 | 1.97E-06 | 1.05E-05 | DOWN |
| IL21R | 2.5353 | -0.0938 | 56.0423 | 7.09E-14 | 1.16E-12 | UP | IL21R | -0.8899 | 0.26549 | 11.3611 | 0.00075 | 0.0023544 | DOWN |
| TSPAN18 | 2.09244 | 0.30137 | 55.6262 | 8.76E-14 | 1.42E-12 | UP | TSPAN18 | -0.7268 | 0.65233 | 12.0816 | 0.0005092 | 0.0016614 | DOWN |
| PHACTR3 | 1.20347 | 2.30433 | 55.0837 | 1.16E-13 | 1.84E-12 | UP | PHACTR3 | -1.8948 | 2.12867 | 146.965 | 7.99E-34 | 6.36E-32 | DOWN |
| CEP19 | 1.01411 | 3.28737 | 54.8057 | 1.33E-13 | 2.10E-12 | UP | CEP19 | -0.5995 | 3.43091 | 36.0814 | 1.89E-09 | 1.68E-08 | DOWN |
| SLC45A2 | 4.13723 | -1.1128 | 53.0957 | 3.18E-13 | 4.82E-12 | UP | SLC45A2 | -0.7358 | -0.6 | 5.07948 | 0.0242106 | 0.0494414 | DOWN |
| CACNG8 | 1.22171 | 1.98558 | 52.6462 | 3.99E-13 | 6.00E-12 | UP | CACNG8 | -1.0729 | 2.02596 | 51.3566 | 7.70E-13 | 1.07E-11 | DOWN |
| ZNF302 | 4.03811 | -1.1925 | 51.9234 | 5.77E-13 | 8.58E-12 | UP | ZNF302 | -1.2333 | -0.8344 | 12.1513 | 0.0004905 | 0.0016086 | DOWN |
| CAPSL | 2.52982 | -0.4714 | 46.3336 | 9.97E-12 | 1.32E-10 | UP | CAPSL | -0.8068 | -0.0939 | 8.74606 | 0.0031027 | 0.0082664 | DOWN |
| XKRX | 1.0976 | 1.8787 | 46.098 | 1.12E-11 | 1.48E-10 | UP | XKRX | -0.6274 | 2.03673 | 19.9393 | 7.99E-06 | 3.83E-05 | DOWN |
| PRKDC | 0.9927 | 9.09048 | 42.3195 | 7.75E-11 | 9.34E-10 | UP | PRKDC | -0.6126 | 9.22022 | 28.7344 | 8.30E-08 | 5.60E-07 | DOWN |
| HPSE2 | 1.95983 | -0.1921 | 41.386 | 1.25E-10 | 1.47E-09 | UP | HPSE2 | -0.9315 | 0.05904 | 13.7005 | 0.0002144 | 0.0007607 | DOWN |
| PCDH18 | 1.2087 | 1.23075 | 39.4436 | 3.38E-10 | 3.81E-09 | UP | PCDH18 | -1.1702 | 1.23818 | 39.6942 | 2.97E-10 | 2.94E-09 | DOWN |
| TNFRSF6B | 0.97379 | 2.63675 | 39.4192 | 3.42E-10 | 3.85E-09 | UP | TNFRSF6B | -0.5861 | 2.77227 | 21.2708 | 3.99E-06 | 2.01E-05 | DOWN |
| SERPINA5 | 3.47178 | -1.3189 | 38.9183 | 4.42E-10 | 4.94E-09 | UP | SERPINA5 | -0.8689 | -0.8986 | 5.81421 | 0.0158972 | 0.0344521 | DOWN |
| KLK9 | 2.46198 | -0.8022 | 38.3349 | 5.96E-10 | 6.55E-09 | UP | KLK9 | -1.8809 | -0.7125 | 27.9189 | 1.27E-07 | 8.31E-07 | DOWN |
| MCM4 | 0.62198 | 8.11452 | 37.5067 | 9.11E-10 | 9.76E-09 | UP | MCM4 | -0.8431 | 8.02533 | 146.938 | 8.10E-34 | 6.41E-32 | DOWN |
| PADI3 | 0.75478 | 5.76581 | 37.4554 | 9.35E-10 | 1.00E-08 | UP | PADI3 | -0.9445 | 5.69263 | 146.761 | 8.85E-34 | 6.97E-32 | DOWN |
| FAM83D | 0.63595 | 6.89837 | 36.4196 | 1.59E-09 | 1.66E-08 | UP | FAM83D | -0.5981 | 6.90737 | 74.088 | 7.47E-18 | 1.74E-16 | DOWN |
| MYH7B | 2.11764 | -0.2332 | 36.0218 | 1.95E-09 | 2.01E-08 | UP | MYH7B | -0.9453 | 0.04189 | 10.6583 | 0.0010958 | 0.0032949 | DOWN |
| EFCAB6 | 2.13059 | -0.6138 | 35.5654 | 2.47E-09 | 2.50E-08 | UP | EFCAB6 | -0.8325 | -0.3083 | 8.67284 | 0.0032299 | 0.008558 | DOWN |
| HAS3 | 0.85227 | 5.07861 | 35.4955 | 2.56E-09 | 2.58E-08 | UP | HAS3 | -1.3005 | 4.92966 | 120.632 | 4.60E-28 | 2.54E-26 | DOWN |
| KIAA1755 | 1.24675 | 0.9959 | 35.2818 | 2.85E-09 | 2.86E-08 | UP | KIAA1755 | -0.9388 | 1.08594 | 25.5713 | 4.26E-07 | 2.55E-06 | DOWN |
| FANCL | 0.93271 | 3.139 | 34.0697 | 5.32E-09 | 5.16E-08 | UP | FANCL | -0.6097 | 3.25103 | 27.7294 | 1.40E-07 | 9.12E-07 | DOWN |
| ABCA1 | 1.05008 | 2.64177 | 31.8819 | 1.64E-08 | 1.47E-07 | UP | ABCA1 | -1.1023 | 2.61971 | 56.1799 | 6.61E-14 | 1.03E-12 | DOWN |
| LGALS12 | 1.41368 | 0.64655 | 31.1344 | 2.41E-08 | 2.11E-07 | UP | LGALS12 | -2.2497 | 0.47078 | 63.981 | 1.26E-15 | 2.32E-14 | DOWN |
| PRR29 | 1.61304 | -0.0483 | 31.057 | 2.51E-08 | 2.19E-07 | UP | PRR29 | -0.8108 | 0.17137 | 10.8049 | 0.0010123 | 0.0030816 | DOWN |
| TTC30B | 0.93296 | 1.98731 | 29.7262 | 4.98E-08 | 4.16E-07 | UP | TTC30B | -0.6538 | 2.08178 | 20.3416 | 6.48E-06 | 3.16E-05 | DOWN |
| INHBA | 0.65442 | 9.19734 | 29.0505 | 7.05E-08 | 5.75E-07 | UP | INHBA | -0.6293 | 9.19975 | 84.5209 | 3.80E-20 | 1.09E-18 | DOWN |
| ANKRD63 | 2.38325 | -0.9666 | 28.6037 | 8.88E-08 | 7.14E-07 | UP | ANKRD63 | -0.8191 | -0.6274 | 5.31016 | 0.0212014 | 0.0440595 | DOWN |
| MNX1 | 1.07786 | 2.42961 | 28.1281 | 1.14E-07 | 8.99E-07 | UP | MNX1 | -0.604 | 2.59266 | 11.6464 | 0.0006433 | 0.0020553 | DOWN |
| PEG10 | 0.65669 | 9.07737 | 27.9185 | 1.27E-07 | 9.96E-07 | UP | PEG10 | -0.7533 | 9.03273 | 77.1012 | 1.62E-18 | 4.03E-17 | DOWN |
| TTC30A | 1.23767 | 2.0029 | 27.5197 | 1.55E-07 | 1.21E-06 | UP | TTC30A | -0.7519 | 2.15589 | 20.1218 | 7.27E-06 | 3.51E-05 | DOWN |
| SVEP1 | 0.72588 | 3.10558 | 27.3288 | 1.72E-07 | 1.33E-06 | UP | SVEP1 | -1.4829 | 2.86424 | 124.935 | 5.26E-29 | 3.04E-27 | DOWN |
| ANKRD36C | 1.25766 | 3.06362 | 27.0309 | 2.00E-07 | 1.53E-06 | UP | ANKRD36C | -0.823 | 3.19628 | 16.7169 | 4.34E-05 | 0.0001791 | DOWN |
| IGFBP4 | 0.64938 | 7.14206 | 25.4435 | 4.56E-07 | 3.33E-06 | UP | IGFBP4 | -1.0441 | 6.99581 | 166.944 | 3.44E-38 | 3.82E-36 | DOWN |
| FCGBP | 1.02363 | 2.33937 | 25.4241 | 4.60E-07 | 3.36E-06 | UP | FCGBP | -2.5743 | 1.9914 | 155.854 | 9.11E-36 | 8.51E-34 | DOWN |
| LONRF2 | 2.22209 | -1.1908 | 24.9219 | 5.97E-07 | 4.30E-06 | UP | LONRF2 | -0.9823 | -0.9322 | 7.3197 | 0.0068203 | 0.0164545 | DOWN |
| GPR19 | 0.8401 | 1.72469 | 24.6087 | 7.02E-07 | 4.99E-06 | UP | GPR19 | -0.6847 | 1.77587 | 19.6702 | 9.20E-06 | 4.36E-05 | DOWN |
| PIANP | 1.77413 | -0.7267 | 24.1604 | 8.86E-07 | 6.19E-06 | UP | PIANP | -1.6896 | -0.7124 | 23.4279 | 1.30E-06 | 7.14E-06 | DOWN |
| TIMM8A | 0.61964 | 4.53489 | 24.1028 | 9.13E-07 | 6.37E-06 | UP | TIMM8A | -0.797 | 4.46248 | 70.3146 | 5.06E-17 | 1.10E-15 | DOWN |
| COL4A6 | 0.67626 | 3.65422 | 23.9557 | 9.86E-07 | 6.83E-06 | UP | COL4A6 | -0.6049 | 3.67557 | 31.4489 | 2.05E-08 | 1.53E-07 | DOWN |
| FZD4 | 0.6947 | 5.62459 | 23.2593 | 1.42E-06 | 9.54E-06 | UP | FZD4 | -1.1759 | 5.45263 | 156.411 | 6.88E-36 | 6.47E-34 | DOWN |
| FLT1 | 1.37688 | -0.0012 | 23.2373 | 1.43E-06 | 9.64E-06 | UP | FLT1 | -0.8 | 0.16492 | 10.2336 | 0.0013791 | 0.0040452 | DOWN |
| ECM2 | 1.2474 | 0.83248 | 22.6924 | 1.90E-06 | 1.26E-05 | UP | ECM2 | -0.755 | 0.9839 | 10.941 | 0.0009406 | 0.0028888 | DOWN |
| PGAM4 | 1.01801 | 1.07741 | 21.8982 | 2.88E-06 | 1.86E-05 | UP | PGAM4 | -0.6254 | 1.2093 | 7.81884 | 0.0051704 | 0.0129256 | DOWN |
| BTBD11 | 1.894 | -1.0195 | 21.7728 | 3.07E-06 | 1.97E-05 | UP | BTBD11 | -1.0629 | -0.8342 | 9.26267 | 0.0023387 | 0.0064453 | DOWN |
| DEPTOR | 1.45202 | -0.0206 | 21.0192 | 4.55E-06 | 2.84E-05 | UP | DEPTOR | -0.7982 | 0.16615 | 9.34988 | 0.00223 | 0.0061733 | DOWN |
| PHOSPHO2 | 0.74625 | 1.96105 | 20.3437 | 6.47E-06 | 3.92E-05 | UP | PHOSPHO2 | -0.6401 | 1.99569 | 18.9643 | 1.33E-05 | 6.10E-05 | DOWN |
| ABCB4 | 1.34833 | 0.282 | 20.163 | 7.11E-06 | 4.27E-05 | UP | ABCB4 | -1.0876 | 0.35151 | 19.0463 | 1.28E-05 | 5.87E-05 | DOWN |
| SLC22A4 | 0.76985 | 2.63515 | 20.0483 | 7.55E-06 | 4.51E-05 | UP | SLC22A4 | -0.8073 | 2.61657 | 45.3922 | 1.61E-11 | 1.89E-10 | DOWN |
| RBL1 | 0.84888 | 5.48919 | 19.9635 | 7.89E-06 | 4.70E-05 | UP | RBL1 | -0.6965 | 5.53699 | 34.0824 | 5.28E-09 | 4.37E-08 | DOWN |
| IGF2BP1 | 1.72536 | -0.3071 | 19.9608 | 7.90E-06 | 4.71E-05 | UP | IGF2BP1 | -0.8373 | -0.0752 | 7.20799 | 0.007258 | 0.0173589 | DOWN |
| SERPIND1 | 0.68901 | 3.15331 | 19.9417 | 7.98E-06 | 4.75E-05 | UP | SERPIND1 | -1.1652 | 2.98761 | 62.6685 | 2.45E-15 | 4.40E-14 | DOWN |
| PRR16 | 0.86065 | 1.64671 | 19.6311 | 9.39E-06 | 5.53E-05 | UP | PRR16 | -0.5996 | 1.73685 | 11.6093 | 0.0006562 | 0.0020932 | DOWN |
| ADORA1 | 1.35346 | -0.1504 | 18.7415 | 1.50E-05 | 8.51E-05 | UP | ADORA1 | -0.6297 | 0.06771 | 6.11818 | 0.0133798 | 0.0297313 | DOWN |
| LOC105377805 | 1.41623 | -0.5205 | 18.5523 | 1.65E-05 | 9.32E-05 | UP | LOC105377805 | -0.9959 | -0.411 | 11.0822 | 0.0008716 | 0.0026999 | DOWN |
| ZNF28 | 1.11223 | 0.18641 | 18.5178 | 1.68E-05 | 9.48E-05 | UP | ZNF28 | -0.6433 | 0.33617 | 7.24348 | 0.0071159 | 0.0170704 | DOWN |
| SERPINB3 | 0.95795 | 2.55036 | 17.597 | 2.73E-05 | 0.000148 | UP | SERPINB3 | -1.6793 | 2.34335 | 59.6802 | 1.12E-14 | 1.90E-13 | DOWN |
| GRAMD2A | 0.80815 | 1.39693 | 17.1173 | 3.51E-05 | 0.000187 | UP | GRAMD2A | -1.0971 | 1.29717 | 33.137 | 8.59E-09 | 6.88E-08 | DOWN |
| ASRGL1 | 0.66925 | 3.21933 | 16.7673 | 4.23E-05 | 0.00022 | UP | ASRGL1 | -0.6209 | 3.23353 | 25.6696 | 4.05E-07 | 2.43E-06 | DOWN |
| LILRB3 | 1.36762 | -0.542 | 16.0873 | 6.05E-05 | 0.000308 | UP | LILRB3 | -0.8729 | -0.4063 | 7.68162 | 0.0055786 | 0.0137881 | DOWN |
| C2orf78 | 1.73805 | -1.2116 | 15.9518 | 6.50E-05 | 0.000328 | UP | C2orf78 | -0.8606 | -1.0017 | 5.42997 | 0.019794 | 0.0416074 | DOWN |
| DMC1 | 2.0006 | -0.7971 | 15.9359 | 6.55E-05 | 0.000331 | UP | DMC1 | -0.9749 | -0.5624 | 5.90505 | 0.0150975 | 0.0330276 | DOWN |
| SERPINB4 | 1.14294 | 1.79633 | 15.9025 | 6.67E-05 | 0.000336 | UP | SERPINB4 | -1.0337 | 1.82491 | 19.5917 | 9.59E-06 | 4.52E-05 | DOWN |
| ARL10 | 1.23936 | -0.381 | 15.7134 | 7.37E-05 | 0.000367 | UP | ARL10 | -0.6715 | -0.2107 | 5.82084 | 0.0158374 | 0.0343537 | DOWN |
| SCG2 | 1.13294 | -0.0107 | 15.0589 | 0.000104 | 0.000503 | UP | SCG2 | -0.6461 | 0.14191 | 5.23141 | 0.0221825 | 0.045803 | DOWN |
| MEOX1 | 0.61318 | 2.87024 | 14.5772 | 0.000135 | 0.000636 | UP | MEOX1 | -0.6507 | 2.8504 | 34.9643 | 3.36E-09 | 2.88E-08 | DOWN |
| FAM186B | 0.93583 | 0.24302 | 14.3747 | 0.00015 | 0.0007 | UP | FAM186B | -0.6856 | 0.32345 | 9.19307 | 0.0024293 | 0.0066665 | DOWN |
| LOC107987158 | 1.14645 | -0.2543 | 14.36 | 0.000151 | 0.000704 | UP | LOC107987158 | -1.1348 | -0.2543 | 13.5064 | 0.0002378 | 0.000836 | DOWN |
| SLC26A9 | 1.023 | 0.13398 | 14.147 | 0.000169 | 0.00078 | UP | SLC26A9 | -0.6605 | 0.25087 | 6.49859 | 0.010796 | 0.024641 | DOWN |
| PFKFB1 | 1.15011 | -0.1516 | 13.5997 | 0.000226 | 0.001015 | UP | PFKFB1 | -0.9123 | -0.0845 | 11.4699 | 0.0007073 | 0.0022362 | DOWN |
| FANCI | 0.59268 | 6.46425 | 13.553 | 0.000232 | 0.001038 | UP | FANCI | -0.7453 | 6.39731 | 49.6748 | 1.81E-12 | 2.40E-11 | DOWN |
| ANXA8L1 | 1.58688 | -1.3623 | 12.422 | 0.000424 | 0.001791 | UP | ANXA8L1 | -1.2337 | -1.2867 | 8.62292 | 0.0033196 | 0.008758 | DOWN |
| FOXP3 | 1.12351 | -0.5723 | 11.8485 | 0.000577 | 0.002363 | UP | FOXP3 | -1.1132 | -0.5736 | 12.3311 | 0.0004455 | 0.0014766 | DOWN |
| DRP2 | 0.72143 | 1.13068 | 11.5028 | 0.000695 | 0.002767 | UP | DRP2 | -1.0796 | 1.00616 | 24.3909 | 7.86E-07 | 4.49E-06 | DOWN |
| H3C1 | 1.13878 | -0.6969 | 10.4128 | 0.001251 | 0.004625 | UP | H3C1 | -0.9324 | -0.6411 | 7.99239 | 0.0046974 | 0.0118947 | DOWN |
| CNTNAP3B | 0.66173 | 1.10532 | 10.0624 | 0.001513 | 0.005475 | UP | CNTNAP3B | -1.4339 | 0.86044 | 48.1322 | 3.98E-12 | 5.04E-11 | DOWN |
| NTF4 | 1.06158 | -0.3415 | 9.74635 | 0.001797 | 0.006363 | UP | NTF4 | -0.9396 | -0.3083 | 8.80211 | 0.0030088 | 0.0080495 | DOWN |
| FSD2 | 0.59935 | 1.0299 | 9.37035 | 0.002205 | 0.007615 | UP | FSD2 | -1.2199 | 0.81929 | 34.6953 | 3.86E-09 | 3.26E-08 | DOWN |
| MROH8 | 1.04236 | -0.7544 | 9.01255 | 0.002681 | 0.00901 | UP | MROH8 | -0.9801 | -0.7407 | 7.59886 | 0.0058405 | 0.0143438 | DOWN |
| RP1L1 | 0.98703 | -0.1618 | 8.97214 | 0.002741 | 0.009186 | UP | RP1L1 | -0.9422 | -0.1519 | 11.143 | 0.0008435 | 0.0026213 | DOWN |
| SAXO2 | 1.18102 | -1.171 | 8.57229 | 0.003413 | 0.011177 | UP | SAXO2 | -0.8856 | -1.0936 | 5.2667 | 0.0217372 | 0.0450139 | DOWN |
| ABCB11 | 0.60153 | 0.93813 | 8.43202 | 0.003687 | 0.01193 | UP | ABCB11 | -0.7396 | 0.88256 | 15.2343 | 9.50E-05 | 0.0003665 | DOWN |
| ZNF154 | 0.80832 | -0.126 | 8.23571 | 0.004107 | 0.013106 | UP | ZNF154 | -0.8416 | -0.1416 | 9.54495 | 0.002005 | 0.0056182 | DOWN |
| TNFAIP8L2-SCNM1 | 0.90237 | 1.02818 | 8.19736 | 0.004195 | 0.013313 | UP | TNFAIP8L2-SCNM1 | -0.9389 | 1.01148 | 6.57913 | 0.0103182 | 0.023674 | DOWN |
| CELF3 | 0.69266 | 0.23091 | 7.89497 | 0.004957 | 0.015406 | UP | CELF3 | -0.8233 | 0.18101 | 10.6442 | 0.0011042 | 0.0033164 | DOWN |
| C11orf65 | 0.94295 | -0.6826 | 7.1547 | 0.007477 | 0.021793 | UP | C11orf65 | -0.9307 | -0.6835 | 7.52162 | 0.0060963 | 0.0149043 | DOWN |
| C5orf63 | 0.7287 | 1.91846 | 6.02371 | 0.014115 | 0.037604 | UP | C5orf63 | -1.615 | 1.65083 | 79.4125 | 5.04E-19 | 1.32E-17 | DOWN |
| ZNF596 | 0.6313 | 0.39314 | 5.9165 | 0.015 | 0.039547 | UP | ZNF596 | -0.9026 | 0.29442 | 15.1883 | 9.73E-05 | 0.0003744 | DOWN |
| RLN1 | 0.62914 | 0.33474 | 5.85688 | 0.015516 | 0.04071 | UP | RLN1 | -1.0231 | 0.19811 | 15.9065 | 6.65E-05 | 0.0002649 | DOWN |
| LOC107985532 | 0.62154 | 0.18193 | 5.44194 | 0.019659 | 0.049711 | UP | LOC107985532 | -0.9452 | 0.06751 | 13.5158 | 0.0002366 | 0.0008326 | DOWN |

**Table S4. TPM of PC-3 cells with KR and KQ in presence or absence of NTZ in RNA-Seq.**

| **genes** | **tpm_KR_C1** | **tpm_KR_C2** | **tpm_KR_C3** | **tpm_KQ_C1** | **tpm_KQ_C2** | **tpm_KQ_C3** | **tpm_KQ_NTZ1** | **tpm_KQ_NTZ2** | **tpm_KQ_NTZ3** |
| --- | --- | --- | --- | --- | --- | --- | --- | --- | --- |
| SMCO4 | 0.922797124 | 1.347175378 | 1.090598159 | -1.124845022 | -1.251748355 | -1.130225069 | 0.143173424 | -0.082419044 | 0.085493405 |
| CYSTM1 | 0.880052007 | 0.633942447 | 0.552467827 | -1.335966847 | -1.345302255 | -1.298251002 | 0.52856738 | 0.676227547 | 0.708262895 |
| FUT3 | 1.302649091 | 0.909046328 | 0.989996693 | -1.121056238 | -1.309214325 | -1.207007594 | 0.206483928 | 0.107820644 | 0.121281473 |
| LOC105377805 | -0.402513117 | -0.970957642 | -1.205538716 | 1.649676526 | 0.766332118 | 1.264525428 | -0.514216011 | -0.073092575 | -0.514216011 |
| IL1RN | 0.662805365 | 0.813100198 | 1.057530892 | -1.653099962 | -1.276524561 | -0.915013716 | 0.417226598 | 0.510261363 | 0.383713823 |
| FAM43A | -1.020634234 | -1.243110671 | -1.086282755 | 1.070914039 | 1.106148448 | 1.355869868 | 0.030723775 | -0.121036038 | -0.092592433 |
| FCGBP | 0.027526911 | -0.155294798 | -0.124290526 | 1.083393387 | 1.65451983 | 0.742003407 | -1.01152108 | -1.01152108 | -1.204816051 |
| BDH1 | 1.329535333 | 1.018585925 | 1.061612482 | -1.092055307 | -1.123095385 | -1.186113028 | 0.280388742 | 0.063738529 | -0.352597291 |
| BICDL1 | 0.887688284 | 1.504529834 | 1.407743122 | -1.037186682 | -0.97607966 | -0.742344713 | -0.352498212 | -0.23750358 | -0.454348393 |
| AADACL3 | -1.035292847 | -1.035292847 | -1.035292847 | 1.342669966 | 1.437200853 | 0.835254678 | 0.100562108 | -0.410971899 | -0.198837165 |
| WNT9A | 1.203396812 | 1.026437201 | 1.189642515 | -0.986534196 | -1.319641121 | -1.162164121 | 0.119988845 | -0.055041757 | -0.016084179 |
| NT5E | 0.880744092 | 1.432185429 | 1.44921705 | -0.716115557 | -0.945413666 | -1.174175994 | -0.311745382 | -0.280180223 | -0.33451575 |
| FAM178B | 1.278687108 | 0.238041623 | 1.194526212 | -0.787031946 | -1.762397243 | -0.947737688 | 0.081227307 | 0.390054739 | 0.314629887 |
| GCNT3 | -0.054456997 | -0.028840556 | -0.132329875 | -1.144167033 | -1.110344368 | -0.977908183 | 0.562390134 | 1.460346464 | 1.425310414 |
| KRT17 | 1.423033537 | 1.123772132 | 1.19170813 | -1.002669463 | -0.985266273 | -1.077915944 | -0.17974796 | -0.237696213 | -0.255217947 |
| NR4A2 | 1.284391807 | 1.462174339 | 0.845802899 | -0.772254904 | -0.557800584 | -1.520627399 | -0.392050327 | 0.001744344 | -0.351380175 |
| PDGFA | 0.607441454 | 0.911513339 | 1.10882471 | -1.48681008 | -1.254764849 | -1.127391296 | 0.404016789 | 0.396680811 | 0.440489122 |
| SHISA3 | -1.216943447 | -1.230175833 | -0.859139037 | 1.419477451 | 0.963310028 | 1.138802794 | -0.161456137 | -0.014238513 | -0.039637307 |
| KDELR2 | 0.757873341 | 1.041384195 | 1.023219173 | -1.362005758 | -1.19182816 | -1.291009049 | 0.385999644 | 0.333579946 | 0.302786668 |
| DEPTOR | -1.635702193 | -0.267959772 | -0.828903218 | 0.907071204 | 0.76254846 | 1.676202854 | -0.162484846 | 0.109039964 | -0.559812452 |
| CNTNAP3B | -0.748611842 | 0.466321369 | 0.025444413 | 1.034613708 | 1.423438851 | 0.883673418 | -1.003056892 | -0.748611842 | -1.333211183 |
| AKR1C3 | 1.187677845 | 1.283015348 | 1.308249013 | -1.136909977 | -0.99055602 | -0.81473946 | -0.479701787 | -0.191948356 | -0.165086607 |
| GBX2 | -1.179142989 | -1.247280444 | -1.179142989 | 1.323287507 | 0.64243453 | 1.175020378 | 0.292166023 | 0.183241065 | -0.010583081 |
| DISP1 | 0.62573442 | 0.301332484 | 1.531708248 | -1.022967485 | -1.650713834 | -0.645998952 | 0.98215523 | -0.134489928 | 0.013239818 |
| NECTIN4 | -0.243142605 | -0.113432522 | -0.109685198 | -0.954087128 | -1.089024193 | -1.156875274 | 1.229265683 | 1.238919246 | 1.198061991 |
| RLN1 | -1.185160094 | 0.104959148 | 0.299098997 | 0.760261251 | 1.455966627 | 1.109559499 | -0.328568046 | -1.07330556 | -1.142811822 |
| ARL10 | 1.341782248 | 0.575712337 | -1.5209066 | 1.46590399 | -0.22966002 | -0.22966002 | -0.367998523 | -1.078586686 | 0.043413273 |
| LOC107985532 | -0.978601459 | -0.546942371 | 0.80654984 | 0.71482564 | 1.357476781 | 1.102702393 | -0.471638397 | -1.399426541 | -0.584945885 |
| HSPB8 | -0.08219685 | -0.18451517 | -0.436515374 | -0.941133551 | -0.989657496 | -1.104464818 | 1.202260968 | 1.163201397 | 1.373020895 |
| CSF1R | 0.78162775 | 1.272201375 | 1.298195977 | -0.990901675 | -1.233622718 | -0.990901675 | -0.427779985 | 0.578177327 | -0.286996377 |
| IL21R | -0.780169024 | -1.100557219 | -1.324102846 | 0.808509809 | 1.156908296 | 1.378383581 | 0.134088736 | 0.353129679 | -0.626191011 |
| MMP9 | -1.312793348 | -1.188741159 | -1.334694292 | 0.976565701 | 0.904179379 | 0.988240603 | 0.412920247 | 0.206172809 | 0.348150059 |
| TENM4 | -1.358369687 | -0.858488995 | -1.087037315 | 1.419852007 | 1.12594536 | 0.870006079 | 0.299245654 | -0.3091711 | -0.101982002 |
| FOXP3 | -0.242427899 | -0.860682196 | -0.607686704 | 1.371211183 | 1.611442368 | 0.816182862 | -0.302171321 | -0.925186097 | -0.860682196 |
| CYP8B1 | 0.138672426 | 0.138672426 | 2 | -0.803987063 | -1.247755761 | -1.023835661 | 0.535145316 | -0.064548152 | 0.239016071 |
| MCM4 | -0.533235211 | -0.177978613 | -0.280477658 | 1.13504415 | 1.504699899 | 1.17529994 | -0.871521691 | -0.916051719 | -1.035779098 |
| ABCA1 | -1.078121834 | -0.231766068 | -0.315821828 | 1.73614253 | 1.348087719 | 0.609280211 | -0.697537057 | -0.517693183 | -0.85257049 |
| NLRC5 | 0.468307065 | 0.804808498 | 0.248151526 | -1.009765326 | -1.146813728 | -1.662518255 | 0.909629066 | 0.995465511 | 0.392735642 |
| SCG2 | -1.241661941 | -0.368189698 | -0.748624721 | 1.496173264 | 1.416176357 | 0.091018445 | 0.51578552 | -0.046354428 | -1.114322796 |
| C2orf78 | -1.10570978 | -1.204499788 | -0.53152404 | 1.949430467 | 0.61529902 | 0.863244506 | -0.165286048 | -0.165286048 | -0.255668289 |
| PHACTR3 | -0.28981155 | -0.201071905 | -0.276915661 | 1.087760024 | 1.441092944 | 1.239147313 | -0.982114707 | -1.0447818 | -0.973304658 |
| FAM83D | -0.790353787 | -0.714418585 | -0.517781399 | 1.23256406 | 1.451554009 | 1.290728269 | -0.551877705 | -0.601473343 | -0.798941519 |
| PCDH18 | -1.061430496 | -0.265151053 | -0.517825366 | 1.547267419 | 1.288574803 | 0.93273102 | -1.061430496 | -0.195065975 | -0.667669855 |
| KIAA0319 | 0.535392889 | 0.572758296 | 0.682158534 | -1.447404256 | -1.260036786 | -1.260036786 | 0.889743929 | 0.764589862 | 0.522834318 |
| SLC26A9 | -1.202877108 | -0.464950633 | -0.792372398 | 0.618301797 | 0.896006134 | 1.874844903 | -0.274444134 | 0.27248555 | -0.926994111 |
| EFCAB6 | -1.342982318 | -0.780603728 | -1.017193205 | 1.648061317 | 0.975079417 | 0.911241448 | -0.179410625 | -0.107096154 | -0.107096154 |
| CALB1 | 1.097407903 | 1.44567152 | 1.421227131 | -0.705799096 | -0.795270761 | -0.779576148 | -0.550478801 | -0.531813554 | -0.601368195 |
| APLN | -1.278145441 | -1.295449448 | -1.271732107 | 0.847181286 | 1.012778614 | 1.001841651 | 0.326303934 | 0.35395587 | 0.303265641 |
| IL36RN | 0.653729373 | 1.163027189 | 1.502868888 | -1.079738089 | -1.297532526 | -1.030973745 | 0.201478481 | -0.024051635 | -0.088807936 |
| COL4A3 | 0.391726983 | 1.466353551 | 0.723345452 | -0.772176723 | -1.251099213 | -1.504037275 | 0.860593863 | -0.0085151 | 0.093808463 |
| PGAM4 | -0.634466313 | -0.664548129 | -1.266786163 | 1.304475043 | 0.690103393 | 1.377226329 | -1.17984333 | 0.272133311 | 0.10170586 |
| NTF4 | -1.03729061 | 0.161348526 | -0.614639071 | -0.083881935 | 1.776217328 | 1.501207328 | -0.870381557 | -0.401335951 | -0.431244059 |
| NRK | -1.302210446 | -1.075845026 | -0.832164445 | 1.263762738 | 1.605008938 | 0.51821623 | -0.028294375 | -0.094073381 | -0.054400233 |
| SERPINB4 | -0.933238847 | 0.097113201 | -1.019564461 | 1.026182167 | 1.968990581 | 0.346602023 | -0.332377741 | -0.220468075 | -0.933238847 |
| ZFPM2 | -1.046490524 | -1.046490524 | -1.046490524 | 1.497089783 | 1.140168432 | 1.03481184 | -0.177347177 | -0.114690914 | -0.240560392 |
| FIBCD1 | 0.463418926 | 0.493598746 | 0.670192854 | -1.190368451 | -1.618469487 | -1.100967847 | 0.894498257 | 0.894498257 | 0.493598746 |
| HSD3B1 | -1.320009613 | -1.29427772 | -0.972670074 | 1.002399738 | 1.253968243 | 0.936113402 | 0.414709771 | -0.08289718 | 0.062663433 |
| WHRN | 0.071648443 | 0.248168218 | 0.913532763 | -1.136677317 | -0.979836059 | -1.64533139 | 0.905314222 | 0.847334102 | 0.775847019 |
| MYBL2 | -0.86031045 | -1.226345717 | -1.088350274 | 1.225539696 | 1.164288706 | 1.277833339 | -0.172738045 | -0.164961458 | -0.154955797 |
| RMDN2 | -0.484613252 | 1.557448315 | 1.084187863 | -0.893000539 | -0.097814761 | -1.694332548 | 0.234359674 | 0.587443334 | -0.293678085 |
| MFSD2A | 0.813357616 | 0.677119865 | 0.708854468 | -1.269011572 | -1.246540875 | -1.436607604 | 0.854619488 | 0.360719739 | 0.537488874 |
| ZNF596 | -0.978350176 | 0.344905646 | -0.81925903 | 1.507994048 | 1.273490026 | 0.738766675 | -1.140887607 | -0.263188529 | -0.663471054 |
| CSF3 | -1.003528853 | -1.060424737 | -1.255031208 | 1.184916048 | 1.197675547 | 1.192839728 | -0.166574555 | -0.055446612 | -0.034425358 |
| CFB | 0.300809977 | 0.498434897 | 0.003572714 | -1.2714477 | -1.38244129 | -1.099982993 | 1.07182427 | 0.873217425 | 1.006012699 |
| TIMM8A | -0.360605155 | -0.445921485 | -0.369822004 | 0.959130128 | 1.566113149 | 1.327998505 | -0.883690337 | -0.827928782 | -0.965274019 |
| HAS3 | -0.273862167 | -0.278587972 | -0.156209181 | 0.940305836 | 1.581732081 | 1.165256522 | -0.724386014 | -1.044789465 | -1.20945964 |
| SGK1 | -1.071530019 | -0.496441327 | -0.806240344 | 1.293741715 | 1.511662109 | 1.080838543 | -0.712931078 | -0.380932407 | -0.418167192 |
| TMEM140 | 1.304955367 | 0.102345259 | 1.338981939 | -0.647639845 | -1.630454092 | -1.085899682 | 0.248442135 | 0.41576755 | -0.046498631 |
| PKIA | 0.871287959 | 1.472968916 | 1.393046811 | -0.976025713 | -0.976025713 | -1.012397763 | -0.25061514 | -0.235980928 | -0.286258428 |
| GDPD3 | 0.517326645 | 0.335732183 | 0.600278811 | -1.965794222 | -1.172967258 | -0.37650771 | 0.154903702 | 0.821240611 | 1.085787239 |
| TMEM269 | 0.730447271 | 0.982135281 | 1.466104169 | -1.652950806 | -0.567048936 | -0.940596967 | 0.406026829 | 0.069975966 | -0.494092807 |
| GPR19 | -0.597498218 | -1.119464771 | -0.405152032 | 1.524891299 | 0.985020947 | 1.249640826 | -0.405152032 | -1.087880129 | -0.14440589 |
| IGFBP4 | -0.022635528 | -0.288054801 | -0.247674878 | 1.065448735 | 1.265738127 | 1.357395396 | -0.971442159 | -1.051832405 | -1.106942486 |
| PFKFB1 | -1.098175448 | 0.206597194 | -0.98018119 | 1.314850867 | 1.609028827 | 0.40326175 | 0.106262533 | -0.581463342 | -0.98018119 |
| SYN3 | 0.766943918 | 1.032146832 | 1.014143204 | -1.266906498 | -1.273152467 | -1.30254153 | 0.472940549 | 0.370196928 | 0.186229063 |
| ASB2 | -0.888941472 | -1.038417705 | -0.894918532 | 1.322552089 | 1.387794771 | 1.14042545 | -0.380738316 | -0.31526933 | -0.332486956 |
| MROH8 | -0.119136286 | -0.819948522 | -0.46132675 | 1.29831699 | 1.064133164 | 0.773063138 | -1.870074917 | -0.231467007 | 0.36644019 |
| ANG | 0.172213338 | -0.177455819 | 0.624016442 | -0.930265956 | -1.318124696 | -1.378961378 | 1.022839146 | 1.10969 | 0.876048923 |
| MNX1 | -0.550445779 | -1.385406792 | -0.899978321 | 0.758232474 | 1.180634356 | 1.457619237 | 0.227530407 | -0.899978321 | 0.11179274 |
| BRINP2 | -1.154346837 | -1.133165928 | -1.0398516 | 1.302576147 | 1.003633777 | 1.222058464 | 0.14254517 | -0.058992736 | -0.284456457 |
| TTC30A | -1.539889583 | -0.853839492 | -0.217503435 | 1.271056861 | 1.629241435 | 0.451591459 | -0.045578977 | -0.087925899 | -0.607152369 |
| BTBD11 | -1.005911815 | -0.830443752 | -1.361775449 | 0.999957492 | 1.62421506 | 0.840641499 | 0.023428057 | 0.023428057 | -0.313539148 |
| PRR16 | -0.984588758 | -1.07865476 | -0.471645524 | 1.066636837 | 1.749826847 | 0.661193817 | -0.971317955 | 0.193143249 | -0.164593752 |
| ZNF154 | -0.840969175 | -0.693160674 | -0.114615195 | 1.510152623 | 1.766282122 | 0.306565662 | -0.546643094 | -0.546643094 | -0.840969175 |
| ARX | 0.206588835 | 0.206588835 | 0.795904557 | -1.324753931 | -1.153799382 | -1.324753931 | 0.504953475 | 1.009469965 | 1.079801579 |
| FOXI1 | -0.956281719 | -0.956281719 | -0.803002919 | 1.3827958 | 1.67184893 | 0.676186747 | -0.231538453 | -0.368609567 | -0.4151171 |
| H3C1 | -0.384414603 | -0.426609829 | -1.408462915 | 0.748911103 | 1.12786904 | 1.743079287 | -0.490931309 | -0.738115042 | -0.171325731 |
| SERPIND1 | -0.334597588 | 0.030932489 | -0.125670592 | 0.825894524 | 1.290619003 | 1.440315379 | -1.100771813 | -0.710467516 | -1.316253887 |
| ASRGL1 | 0.291925761 | -0.872976403 | -0.900952256 | 1.14214597 | 0.949775542 | 1.488035752 | -0.149933149 | -0.962138258 | -0.98588296 |
| FANCL | -1.509390038 | -0.410080238 | -0.704343829 | 1.439900346 | 1.43543555 | 0.648571442 | -0.045072257 | -0.167240823 | -0.687780153 |
| TTC9 | 0.928705664 | 0.834199744 | 0.952114184 | -1.033932668 | -1.63647469 | -1.130472337 | 0.209393853 | 0.23560175 | 0.6408645 |
| LILRB3 | -0.786330245 | -0.912135489 | -0.786330245 | 0.369828875 | 1.697067399 | 1.421327028 | -0.848975066 | 0.207124235 | -0.361576492 |
| DMC1 | -0.415153842 | -1.032018545 | -1.032018545 | 2 | 0.063895347 | 0.92072103 | 0.285856259 | -0.2730956 | -0.599154931 |
| TNFAIP8L2-SCNM1 | -0.092891595 | -0.988972083 | -0.0800871 | 0.729604244 | 1.866824716 | 0.224576065 | -0.546685286 | -1.561558289 | 0.44918933 |
| PIANP | -0.99311883 | -0.520576841 | -0.390796347 | 1.430902893 | 1.630703213 | 0.742657288 | -0.923785252 | -0.455409282 | -0.520576841 |
| PRR29 | -0.968844861 | -1.303145502 | -0.740741015 | 1.068891458 | 1.001838476 | 1.580461539 | -0.063055673 | -0.308499873 | -0.266904549 |
| ABCB4 | -0.806845201 | -1.273518514 | -0.188509592 | 0.736741893 | 1.878336427 | 0.967153253 | -0.707674522 | -0.325908671 | -0.279775073 |
| PRKDC | -1.523200625 | -0.777897214 | -0.419508431 | 1.630849285 | 1.329838601 | 0.383096417 | 0.023198858 | -0.084017199 | -0.56235969 |
| PDZK1 | 1.51738846 | 1.42135317 | 0.357135127 | -0.439964589 | -1.297244108 | -1.092578313 | -0.241350934 | 0.332658348 | -0.557397161 |
| FRY | 0.100768778 | 0.622571511 | 0.980783279 | -0.619689934 | -1.597486446 | -1.415551206 | 0.342395266 | 1.196395295 | 0.389813458 |
| SLITRK5 | -1.302973761 | -0.916478762 | -1.155023516 | 1.106234392 | 1.320831613 | 0.919120803 | 0.146510096 | 0.319478861 | -0.437699726 |
| TSPAN18 | -1.359122711 | -1.311716703 | -0.688809765 | 1.543126196 | 0.897674127 | 0.86639462 | 0.091138076 | -0.093418083 | 0.054734243 |
| BDKRB2 | -1.014589006 | -1.001325835 | -0.726368141 | 1.39715748 | 1.295815916 | 1.168391953 | -0.468545792 | -0.405528171 | -0.245008404 |
| LOC105373989 | -1.102921031 | -1.362274293 | -1.111119046 | 1.239264311 | 0.896868647 | 1.141280201 | 0.17466136 | 0.140523227 | -0.016283375 |
| CEP19 | -1.460499315 | -0.697398375 | -0.815929749 | 1.499673147 | 1.255068473 | 0.864540622 | -0.217757708 | -0.167436409 | -0.260260686 |
| CCNA1 | 0.926825359 | 1.317798559 | 1.00385823 | -1.330203875 | -0.923364589 | -1.299010418 | 0.289600408 | 0.04739986 | -0.032903534 |
| TJP3 | 1.165730975 | 0.712566498 | 1.271362953 | -0.895151613 | -1.760105839 | -0.764238447 | 0.011470086 | 0.136904174 | 0.121461213 |
| MVP | 1.125089504 | 0.661062778 | 0.652073363 | -1.251179151 | -1.588069019 | -1.033785342 | 0.597681503 | 0.482718767 | 0.354407596 |
| TNFRSF11B | -0.428896209 | 0.098506614 | 0.076344049 | 1.322001346 | 1.247625214 | 0.951300908 | -0.964875702 | -1.07791076 | -1.224095461 |
| LTB | -1.10756819 | -1.342141857 | -1.26244563 | 1.039565396 | 0.905576038 | 1.156436293 | 0.297970729 | 0.191100929 | 0.121506292 |
| SDCBP2 | 1.018442349 | 1.183762918 | 0.885553509 | -1.205518306 | -1.500544736 | -0.971688391 | 0.117270526 | 0.199599571 | 0.27312256 |
| ANKRD37 | 0.640242587 | -0.008175969 | 0.146135073 | -1.211781577 | -0.604758454 | -1.722882019 | 0.820655291 | 0.646379926 | 1.294185142 |
| UST | 0.675426084 | 1.439684019 | 1.063212437 | -1.261858032 | -0.951210386 | -1.327215744 | 0.259897341 | 0.11938832 | -0.017324038 |
| TMOD1 | 0.06403508 | 0.490228215 | -0.136171652 | -1.280124589 | -1.296225735 | -0.917889512 | 1.342404254 | 1.234709301 | 0.499034639 |
| SLC1A1 | -0.051255906 | 1.29947786 | 1.153909983 | -0.893449487 | -0.960226104 | -1.629931352 | 0.007137387 | 0.644658119 | 0.429679499 |
| IGSF23 | -1.679134566 | -1.08922569 | -0.671308802 | 1.137784996 | 1.097804234 | 0.97724642 | 0.213787692 | -0.139951247 | 0.152996962 |
| MAP2 | -0.13117213 | 0.952793962 | 0.59691466 | -1.20088339 | -0.756516856 | -1.685496507 | 1.038186719 | 0.795568598 | 0.390604946 |
| FLRT1 | -0.988185949 | -1.472517186 | -1.087718853 | 1.325145666 | 0.845650109 | 1.077416496 | 0.025005042 | 0.165832276 | 0.1093724 |
| SLC41A2 | 0.225585838 | 1.284083703 | 0.912153481 | -1.002758612 | -1.16946563 | -1.58206276 | 0.453388016 | 0.430295141 | 0.448780824 |
| ANKRD36C | -1.213978086 | -0.732463156 | -0.481489659 | 1.780075562 | 1.484312364 | 0.015682378 | 0.001586258 | -0.323426297 | -0.530299364 |
| AKR1B1 | 0.583884963 | 0.310464086 | 0.271630305 | -1.398955503 | -1.156469563 | -1.269013436 | 0.95416981 | 0.51952414 | 1.184765198 |
| SLC44A4 | 0.573710347 | 0.658647593 | 0.623509783 | -1.281922387 | -1.503072271 | -1.18534308 | 0.552146636 | 0.868890772 | 0.693432607 |
| WNT4 | -0.156838247 | -0.127275605 | -0.391933681 | -0.821691505 | -1.575995357 | -0.516510238 | 0.981376824 | 1.221749384 | 1.387118425 |
| SERINC5 | -0.187075261 | 0.819710415 | 0.963622523 | -0.515262671 | -0.984770872 | -1.95966761 | 0.797737696 | 0.7386356 | 0.32707018 |
| SLC45A2 | -1.277757403 | -1.162167399 | -1.219681838 | 0.733491548 | 0.691718056 | 1.5127329 | 0.257021468 | 0.073847071 | 0.390795596 |
| RBL1 | -1.642815986 | -0.325828366 | -0.065203301 | 1.621056472 | 1.366919643 | 0.300988154 | -0.196545952 | -0.479603621 | -0.578967043 |
| BTN3A3 | 0.84478237 | 1.110655189 | 1.120196363 | -1.086311531 | -1.510385965 | -1.070496458 | 0.104765747 | 0.068487827 | 0.418306458 |
| APOL2 | 0.797302495 | 0.20663863 | 0.680192273 | -1.31325743 | -1.268319772 | -1.351139402 | 0.654125941 | 0.820273601 | 0.774183664 |
| METRNL | 1.473571699 | 1.206131788 | 1.087904779 | -0.9214603 | -1.210214207 | -0.763676407 | -0.141010652 | -0.42103866 | -0.31020804 |
| RND1 | 1.126825992 | 0.950278123 | 0.52791149 | -1.109739462 | -1.382975009 | -1.33268732 | 0.200488765 | 0.325481955 | 0.694415467 |
| NAV1 | 0.346404763 | 0.387785694 | 0.679576826 | -1.109477848 | -1.370727773 | -1.412377852 | 0.964702456 | 0.964702456 | 0.549411278 |
| IFITM10 | 0.257477416 | 0.054491882 | 0.761564861 | -0.7088719 | -1.908462768 | -0.935232788 | 0.593967113 | 0.624861162 | 1.260205022 |
| ABCB11 | -0.63361107 | 0.317537009 | -0.998973404 | 1.199101545 | 1.27613717 | 1.199101545 | -0.63361107 | -1.092070656 | -0.63361107 |
| LGALS12 | -0.297408584 | -0.126380056 | -0.480497555 | 0.849227272 | 1.556791401 | 1.297888024 | -0.773688073 | -0.709153583 | -1.316778846 |
| PHOSPHO2 | -1.342455285 | -0.257521596 | -0.443695394 | 1.739135228 | 0.716553123 | 1.215700407 | -0.69410134 | -0.489919748 | -0.443695394 |
| ADORA1 | -0.309983522 | -1.1881836 | -1.441363038 | 1.606274569 | 0.830014435 | 0.793975847 | 0.147226443 | -0.6648406 | 0.226879467 |
| PEG10 | -1.128782409 | -0.278701692 | 0.067818351 | 1.574810476 | 1.411459572 | 0.58074645 | -0.490809508 | -0.661652617 | -1.074888623 |
| LRRK1 | 0.89520335 | 1.113791833 | 1.254788901 | -1.012200157 | -1.124806283 | -1.402414384 | 0.220143458 | 0.22405787 | -0.168564586 |
| IL32 | 1.209428532 | 0.922319176 | 0.952131896 | -1.221643494 | -1.485942681 | -0.97418847 | 0.191508038 | 0.127951765 | 0.278435239 |
| FANCI | -1.346122488 | 0.152749252 | 0.150568995 | 1.628792182 | 1.230803163 | 0.377218368 | -0.496718278 | -0.57967622 | -1.117614974 |
| ARSK | -0.717022698 | 1.215292583 | 0.780512023 | -0.836639821 | -0.906269073 | -1.500288266 | 0.990433872 | 0.780512023 | 0.193469359 |
| PITPNM3 | 0.271916117 | 0.271916117 | 0.81466326 | -0.95230664 | -1.597875521 | -1.203909193 | 0.848804101 | 0.347834612 | 1.198957148 |
| TTC30B | -1.267146461 | -0.79110301 | -0.497617866 | 1.346207653 | 1.670552678 | 0.657330664 | -0.220709501 | -0.57969394 | -0.317820218 |
| CAPSL | -1.178501701 | -1.229693163 | -1.095460583 | 0.844158239 | 1.560254731 | 0.851625006 | 0.139881252 | 0.079569834 | 0.028166384 |
| PRKAR1B | 0.942267701 | 0.720038024 | 0.637724221 | -1.487328967 | -1.156566606 | -1.29482358 | 0.432357633 | 0.451390117 | 0.754941459 |
| FA2H | 0.88884127 | 0.724718598 | 0.771973015 | -1.123337361 | -1.639072358 | -1.160227343 | 0.448807286 | 0.561559077 | 0.526737816 |
| TMPRSS2 | 1.240993186 | 1.144814393 | 1.014007668 | -1.125365061 | -1.312904395 | -1.060078753 | 0.007164611 | 0.007164611 | 0.08420374 |
| SPOCK2 | -0.343527681 | 0.717441001 | 0.551654032 | -1.318427739 | -0.586912 | -1.542656927 | 0.717441001 | 0.424621694 | 1.380366621 |
| IGF2BP1 | -0.493912863 | -1.113865651 | -0.987546395 | 1.834466941 | 1.315857468 | -0.017912918 | -0.615617651 | 0.213859562 | -0.135328494 |
| STK32A | 0.341365847 | 1.449663349 | 1.32096044 | -0.880651952 | -1.134798749 | -1.310967543 | -0.261680771 | 0.046690911 | 0.429418469 |
| NKD1 | -1.05903917 | -1.209076608 | -1.209076608 | 0.899633311 | 0.767782472 | 1.542288872 | 0.090840945 | 0.365238943 | -0.188592157 |
| PTPRR | -0.373517154 | 0.511469297 | 0.487324586 | -1.033247865 | -0.974491599 | -1.524693666 | 0.65947156 | 1.294907797 | 0.952777045 |
| SERPINA3 | -1.214803725 | -1.233830301 | -1.210078458 | 1.055201175 | 1.018809528 | 1.150048613 | 0.138279556 | 0.164781092 | 0.13159252 |
| KLK9 | -0.669297418 | -0.614104152 | -1.082319975 | 1.200209557 | 1.32782398 | 1.374497201 | -0.225920503 | -0.641591273 | -0.669297418 |
| ESPN | 1.406588378 | 0.710652681 | 1.067946224 | -1.344593924 | -1.416882289 | -0.720212486 | 0.181302952 | 0.023891707 | 0.091306757 |
| FLI1 | -1.311527124 | -1.27595269 | -1.123048229 | 0.996646612 | 1.132735446 | 0.978986595 | 0.162992926 | 0.091530683 | 0.347635781 |
| FZD4 | -0.788500645 | 0.074596413 | 0.278117774 | 1.365950585 | 1.360974299 | 0.711026022 | -0.82476624 | -0.986347412 | -1.191050796 |
| PADI3 | -0.320324966 | -0.443906949 | -0.489911906 | 1.342290986 | 1.253295014 | 1.304563826 | -0.695674467 | -0.981688241 | -0.968643296 |
| CELF3 | -0.807216282 | -0.377110596 | -0.096406267 | 1.240428173 | 1.746573406 | 0.451233434 | -0.662613522 | -0.096406267 | -1.398482079 |
| C5orf63 | 0.600537354 | -1.000663709 | -0.359353313 | 1.213102002 | 0.746149787 | 1.412265855 | -1.246439839 | -0.682799068 | -0.682799068 |
| S100A6 | 1.356853482 | 1.131611021 | 0.962198203 | -1.22921356 | -1.085081178 | -1.109836959 | -0.137489841 | -0.005410405 | 0.116369237 |
| AKR1C1 | 0.938493208 | 0.927782397 | 0.818776923 | -1.549489793 | -1.112103295 | -1.131703808 | 0.694691927 | -0.018500413 | 0.432052854 |
| ASDURF | -0.768798691 | 1.685935262 | 0.464277308 | -0.173351411 | -1.283145661 | -1.309162239 | 0.191257577 | 0.922384021 | 0.270603835 |
| PROS1 | -0.46727172 | 1.035533575 | 0.991665447 | -0.708329187 | -0.912105567 | -1.797459235 | 0.540332912 | 0.684907605 | 0.63272617 |
| LY6K | -1.287091705 | -1.448125695 | -1.087693061 | 1.01579263 | 0.93869051 | 0.919600747 | 0.361000138 | 0.266348859 | 0.321477576 |
| COL4A6 | -1.321291646 | -0.526160917 | -0.282550512 | 1.276960731 | 1.758570934 | 0.557234287 | -0.171137704 | -0.659264136 | -0.632361036 |
| CGB5 | 0.655748264 | 0.655748264 | 0.466117554 | 0.322443645 | -1.94049365 | -1.460371698 | 0.916722178 | 0.24866199 | 0.135423454 |
| SGK3 | -0.121255065 | 1.438241202 | 0.852759988 | -1.334706614 | -0.009349804 | -1.641153499 | -0.29776923 | 0.612899223 | 0.500333801 |
| FXYD3 | 1.313419575 | 1.285965596 | 1.275073538 | -1.015007149 | -0.754674502 | -0.968840369 | -0.373270405 | -0.265524087 | -0.497142196 |
| PDGFRL | 0.710569604 | 0.69221662 | 0.765137526 | -1.976311642 | -1.246376685 | -0.446375486 | 0.617966353 | 0.434600493 | 0.448573216 |
| INHBB | -1.137593167 | -1.225359264 | -1.086748043 | 1.208168429 | 1.110281464 | 1.147442171 | 0.086609596 | -0.023557556 | -0.079243631 |
| FLT1 | -0.790640414 | -0.681260547 | -1.237970647 | 1.782322477 | 1.04540905 | 0.66002741 | 0.161000634 | -0.14824755 | -0.790640414 |
| GALNT12 | 0.592604417 | 1.258818275 | 1.186313108 | -0.894464382 | -1.455489798 | -1.272030617 | 0.164067138 | 0.260511011 | 0.159670848 |
| MSMP | -1.190057355 | -0.80906719 | -1.300935081 | 1.158798033 | 1.243908161 | 1.143355866 | -0.194622067 | -0.016719723 | -0.034660643 |
| SVEP1 | -0.27371957 | -0.127417114 | 0.129068337 | 1.030089932 | 1.247510185 | 1.265164207 | -0.82672923 | -1.267855432 | -1.176111315 |
| ANXA8 | 0.833281352 | 1.196350468 | 0.766409427 | -0.738658164 | -1.115137353 | -1.147815502 | 0.942803142 | 0.345435984 | -1.082669353 |
| SAXO2 | -1.145566703 | -0.200308068 | -0.924189157 | 1.043899478 | -0.081205754 | 1.569569547 | -1.29996574 | 0.569718613 | 0.468047783 |
| TCN1 | -1.244215987 | -1.232468733 | -1.354830517 | 0.90394147 | 0.973769544 | 0.948584853 | 0.41357303 | 0.088258972 | 0.50338737 |
| MEOX1 | -0.491912681 | -0.981560257 | -0.571437341 | 1.526834926 | 1.084265705 | 1.296158797 | -0.38799456 | -0.589299638 | -0.88505495 |
| TNFRSF6B | -0.844822525 | -1.089328734 | -1.055159189 | 1.276296917 | 1.119825115 | 1.30026274 | -0.58266721 | 0.118644379 | -0.243051492 |
| FAM186B | -1.554482883 | -0.427914551 | -0.378256115 | 1.418362906 | 1.457242712 | 0.724674903 | -0.578887356 | -0.18283667 | -0.477902945 |
| ZNF28 | 0.521602871 | -1.033588132 | -1.365080441 | 0.737773051 | 1.030255599 | 1.351051254 | 0.115967291 | -1.142456475 | -0.215525018 |
| SAT1 | 0.217322034 | 0.798175245 | 0.652922531 | -1.195976522 | -1.383735447 | -1.353572025 | 0.763242254 | 0.717903691 | 0.783718239 |
| MUC4 | -0.137368432 | 0.514185667 | 0.24918515 | -1.035246313 | -0.785689404 | -1.695860192 | 0.514185667 | 1.164367975 | 1.212239884 |
| CHPF2 | 0.687793614 | 0.371167227 | 0.426429595 | -1.618178299 | -1.320534137 | -0.923921752 | 1.060131041 | 0.650841348 | 0.666271363 |
| ATP2A3 | 1.23218418 | -0.21314667 | 0.69430101 | -1.312039987 | -0.096350483 | -1.698985655 | 0.803592138 | 0.803592138 | -0.21314667 |
| FER1L6 | 0.640108685 | 0.940311984 | 1.743141485 | -0.742021761 | -0.987184578 | -1.459758524 | -0.047804028 | 0.008831794 | -0.095625058 |
| TBX2 | 1.403249659 | 0.440296925 | 0.214415246 | -0.984749856 | -1.509790652 | -1.196747853 | 0.467973183 | 0.257387199 | 0.907966149 |
| C11orf65 | -0.6347534 | -0.18795944 | -0.697411983 | 1.982678458 | 1.068487602 | 0.52880433 | -1.056584883 | -0.274226912 | -0.729033772 |
| FSD2 | -0.327583462 | 0.012697675 | -0.043026575 | 1.618419539 | 1.198213492 | 0.652938499 | -0.928767194 | -1.378006023 | -0.804885952 |
| ECM2 | -1.156829623 | -0.318369593 | -1.059627338 | 1.317000175 | 1.745809261 | 0.250608845 | -0.514600285 | 0.250608845 | -0.514600285 |
| IDO1 | 0.845393291 | 1.391977861 | 1.24928423 | -1.069697199 | -1.062168794 | -1.21621338 | -0.051980723 | -0.163538522 | 0.076943235 |
| TNFSF14 | -0.296695212 | -0.121974359 | 0.459436362 | -0.942500184 | -0.965966355 | -1.467354936 | 1.093312427 | 1.162392799 | 1.079349457 |
| RORC | 1.036096325 | 0.189047706 | 0.070656441 | -1.733825948 | -1.189799495 | -0.50977529 | 0.606880463 | 0.189047706 | 1.341672092 |
| HID1 | 0.541038384 | 0.186032354 | 0.291389922 | -0.895436501 | -1.567494356 | -1.313307836 | 1.10118228 | 0.729855931 | 0.926739822 |
| SERPINB3 | -0.56666064 | 0.165785058 | 0.039837634 | 0.763998714 | 1.796844579 | 0.828832038 | -1.230490393 | -0.799715387 | -0.998431603 |
| SLFN11 | 1.169084978 | 1.376623824 | 1.42269051 | -0.643216883 | -0.741442722 | -0.892304389 | -0.586546986 | -0.552444167 | -0.552444167 |
| LOC105374299 | -0.342587658 | -0.306399015 | 0.109860066 | -0.876614654 | -0.83665913 | -1.336926429 | 0.82745686 | 1.275549057 | 1.486320902 |
| ABAT | -0.104985106 | 0.746740078 | 0.519009175 | -1.3428132 | -0.890448342 | -1.401181052 | 1.268463833 | 0.968230987 | 0.236983628 |
| CXCL5 | -1.074245579 | -0.61299035 | -0.809584113 | 1.11043464 | 1.610973197 | 1.159748769 | -0.487745431 | -0.419464528 | -0.477126604 |
| PLEKHG1 | 0.841420102 | 1.21952197 | 1.417490152 | -0.641443361 | -0.939444432 | -1.56611846 | -0.07346558 | -0.057921714 | -0.200038677 |
| PLEKHA7 | 1.105593657 | 1.199497335 | 1.47382668 | -0.906408628 | -0.832354045 | -1.183712877 | -0.309607627 | -0.219719806 | -0.327114689 |
| MEF2C | 0.478456085 | 1.535871183 | 1.007381413 | -1.181014367 | -0.185589884 | -1.453473505 | 0.307397648 | -0.816426222 | 0.307397648 |
| STARD4 | -0.391726642 | 0.714328144 | 0.330698401 | -0.801932891 | -0.656973574 | -1.908114905 | 0.966085251 | 1.033308071 | 0.714328144 |
| CYP2E1 | -0.150504622 | 1.283117481 | 0.268462278 | 0.268462278 | -1.069467015 | -2 | 0.393100272 | 0.804150272 | 0.24735162 |
| RPP25 | -1.009745411 | -1.350862357 | -1.390685127 | 0.94949991 | 1.039124947 | 1.029987437 | 0.238673681 | 0.233064768 | 0.260942151 |
| SERPINF2 | 1.247499117 | 0.77607452 | 0.729072272 | -1.189978066 | -1.688517348 | -0.833974137 | 0.350317404 | 0.165495784 | 0.444010453 |
| CAPN12 | 1.293489424 | 0.899490606 | 0.586945128 | -0.1531479 | -2 | -0.918734846 | 0.129019025 | 0.113845391 | 0.113845391 |
| ADIRF | 1.496789569 | 1.202454518 | 1.180127574 | -1.148226659 | -0.728423315 | -0.697199045 | -0.447718326 | -0.522565678 | -0.335238639 |
| UGT2B7 | 1.211724575 | 1.362865435 | 1.327230308 | -0.857041657 | -0.895752271 | -0.944059109 | -0.406617681 | -0.381540268 | -0.416809332 |
| KIAA1755 | -0.833333792 | -0.833333792 | -1.117835721 | 1.251899994 | 1.046774875 | 1.515696206 | -0.472402668 | -0.385218958 | -0.172246143 |
| RP1L1 | -1.072376582 | 0.594949462 | -1.219586582 | 0.726277692 | 0.985709091 | 1.49215106 | -0.078554773 | -0.356192788 | -1.072376582 |
| TRIM31 | 1.122053226 | 1.322275643 | 1.399304735 | -0.772366901 | -0.852615422 | -0.988000462 | -0.680077225 | -0.029511832 | -0.521061763 |
| CYP1B1 | -0.174268752 | 1.17384891 | 1.209501391 | -0.611038933 | -0.94592272 | -1.802075715 | 0.659289215 | 0.415863576 | 0.074803028 |
| CD14 | -0.914027721 | -0.217947192 | -0.310022764 | 1.238344373 | 1.272486095 | 1.339110341 | -1.105289016 | -0.601898342 | -0.700755775 |
| SLC22A4 | -0.068879211 | -0.77688212 | -0.759895755 | 1.192690256 | 1.564962681 | 1.081930457 | -0.618351731 | -0.602024023 | -1.013550554 |
| IGDCC4 | -0.060972706 | 0.505275987 | 1.303573983 | -1.425803415 | -1.018862789 | -1.219050753 | 1.024837274 | 0.582171955 | 0.308830465 |
| DMPK | 1.109001452 | 0.806953453 | 0.942248446 | -1.190732614 | -1.526882527 | -1.075431453 | 0.419044598 | 0.349332506 | 0.166466139 |
| FTH1 | 0.29005721 | 0.068995091 | 0.023452027 | -1.154744296 | -1.425374149 | -1.031896244 | 1.049972383 | 1.051130427 | 1.128407551 |
| ARG2 | -0.3063064 | -0.399860472 | -0.12054875 | -0.939550025 | -0.962594843 | -1.057854235 | 1.137146456 | 1.333422998 | 1.31614527 |
| PRRT4 | -1.262059527 | -1.010303241 | -1.262059527 | 1.257772166 | 0.662599319 | 1.308378617 | 0.015778624 | 0.241247014 | 0.048646555 |
| INHBA | -1.490867348 | -0.053307266 | -0.245460621 | 1.525065072 | 1.312697258 | 0.72716317 | -0.404626467 | -0.556150003 | -0.814513796 |
| SERPINA5 | -1.001771424 | -1.356912806 | -1.001771424 | 0.472185478 | 1.314193743 | 1.431031877 | 0.288867318 | 0.099863976 | -0.245686737 |
| TNF | -0.945895688 | -1.133845813 | -1.144097082 | 0.9808056 | 1.394915543 | 1.014203564 | -0.664038911 | 0.317579795 | 0.180372992 |
| SRPX | 0.621457625 | 0.357288174 | 1.123100533 | -0.886524486 | -1.92546102 | -0.916866562 | 0.512134728 | 0.498021621 | 0.616849386 |
| COL4A4 | 0.693970428 | 1.382146512 | 1.544181382 | -0.81134391 | -0.867505214 | -1.214208207 | 0.031234984 | -0.537060025 | -0.22141595 |
| KLHDC7B | 0.229067474 | -0.377062434 | 0.279335523 | -0.604695075 | -1.338700803 | -1.432050571 | 1.012736211 | 1.073029675 | 1.158339998 |
| XKRX | -1.350416754 | -0.99930421 | -0.964054075 | 1.127704019 | 1.241319453 | 1.043027522 | -0.484642902 | 0.05470925 | 0.331657696 |
| LONRF2 | -1.146875762 | -1.146875762 | -0.726617888 | 1.314720377 | 1.314720377 | 0.914193216 | 0.101676665 | -0.726617888 | 0.101676665 |
| LOC107987158 | -1.183244665 | -0.555468747 | -0.072933824 | 0.844523143 | 1.064709993 | 1.494867881 | 0.162181641 | -0.312103202 | -1.44253222 |
| DRP2 | -0.427712924 | -0.487025915 | 0.031768496 | 2 | 0.726917568 | 0.726917568 | -0.667617501 | -1.300454124 | -0.606971621 |
| MYH7B | -0.774115969 | -1.134916335 | -1.043455605 | 0.471044501 | 1.505097485 | 1.505097485 | -0.426204121 | -0.256799917 | 0.154252476 |
| CACNG8 | -0.677094513 | -0.715720631 | -0.75461406 | 1.498052204 | 1.471851327 | 0.920421317 | -0.525191264 | -0.304767794 | -0.912936587 |
| TRIM29 | 1.312893299 | 1.25944829 | 1.293029947 | -1.033630716 | -0.846979694 | -0.921594353 | -0.321992927 | -0.41918092 | -0.321992927 |
| ANXA8L1 | -0.879510931 | -0.879510931 | -0.550998524 | 0.747256643 | 1.220799254 | 1.809794723 | -0.297978324 | -0.423442838 | -0.746409072 |
| MINAR1 | -0.615963849 | 0.913862274 | 0.414422162 | -0.877402216 | -1.078815392 | -1.354896741 | 1.487456364 | 0.471364981 | 0.639972417 |
| MAP7D3 | -1.420929832 | -1.154682661 | -1.011732457 | 1.096297901 | 1.172545242 | 0.997661438 | 0.166062428 | 0.156285072 | -0.001507132 |
| ZNF302 | -1.214907507 | -0.715755993 | -1.214907507 | 1.239543692 | 1.371462179 | 0.920880437 | -0.479883864 | 0.180255151 | -0.086686588 |
| HPSE2 | -1.106455306 | -0.758735996 | -1.166198582 | 1.355893475 | 0.974152598 | 1.355893475 | -0.428124711 | -0.164519385 | -0.06190557 |
| GRAMD2A | -0.286818258 | -0.193833753 | -0.60219521 | 1.37986851 | 1.606061023 | 0.724603178 | -1.217063714 | -0.833441346 | -0.57718043 |
| ANKRD63 | -1.103202337 | -0.925531697 | -0.925531697 | 0.153506039 | 1.735777076 | 1.190954982 | 0.153506039 | -0.665214526 | 0.385736123 |

**Table S5. The significance of overall survival of NTZ-downregulated genes or NTZ-upregulated genes in SU2C database.**

| **number** | **gene** | **median** | **pValue_logrank** | **change of NTZ treatment** | **number** | **gene** | **median** | **pValue_logrank** | **change of NTZ treatment** |
| --- | --- | --- | --- | --- | --- | --- | --- | --- | --- |
| 1 | PIANP | 0.565920904 | 0.002620633 | down | 1 | SPOCK2 | 2.43886644 | 0.014269849 | up |
| 2 | C2orf78 | 0 | 0.01497283 | down | 2 | BDH1 | 4.98534919 | 0.024702895 | up |
| 3 | TIMM8A | 3.574577183 | 0.016752014 | down | 3 | FTH1 | 8.46212111 | 0.03328519 | up |
| 4 | INHBB | 4.048218779 | 0.039787031 | down | 4 | COL4A3 | 0.4163165 | 0.043362368 | up |
| 5 | COL4A6 | 0.290939377 | 0.043387153 | down | 5 | COL4A4 | 0.51072035 | 0.043745408 | up |
| 6 | MYBL2 | 4.609446737 | 0.043887896 | down | 6 | CALB1 | 0.07771719 | 0.048162725 | up |
| 7 | CACNG8 | 0.113298268 | 0.048925006 | down | 7 | TMPRSS2 | 8.70485271 | 0.048542182 | up |
| 8 | FLRT1 | 0.991087147 | 0.058466986 | down | 8 | DISP1 | 2.32639948 | 0.061885205 | up |
| 9 | ECM2 | 2.568415412 | 0.062100841 | down | 9 | MEF2C | 2.9827504 | 0.087863395 | up |
| 10 | MEOX1 | 1.046975627 | 0.064704414 | down | 10 | DMPK | 4.24421897 | 0.09395527 | up |
| 11 | FSD2 | 1.294344775 | 0.067665516 | down | 11 | CYSTM1 | 5.4487822 | 0.098904004 | up |
| 12 | TNFRSF11B | 1.372828524 | 0.082324931 | down | 12 | MUC4 | 0.12540744 | 0.115264259 | up |
| 13 | MCM4 | 5.300407621 | 0.08539643 | down | 13 | NR4A2 | 2.27005445 | 0.139755577 | up |
| 14 | FZD4 | 4.431031806 | 0.089858484 | down | 14 | CYP8B1 | 0.08176889 | 0.140550858 | up |
| 15 | PRRT4 | 0.453197026 | 0.109818093 | down | 15 | MAP2 | 2.84654625 | 0.147643936 | up |
| 16 | RP1L1 | 0.054925047 | 0.109886591 | down | 16 | SMCO4 | 6.13451156 | 0.151113867 | up |
| 17 | BTBD11 | 1.287486838 | 0.116318865 | down | 17 | RND1 | 2.27992458 | 0.151593078 | up |
| 18 | ZNF302 | 4.65083722 | 0.120100607 | down | 18 | TTC9 | 2.74631442 | 0.161137901 | up |
| 19 | KIAA1755 | 0.932805073 | 0.121432743 | down | 19 | SLC41A2 | 2.5011655 | 0.163528209 | up |
| 20 | IGF2BP1 | 0.029202898 | 0.131902452 | down | 20 | CYP2E1 | 1.35624149 | 0.168026342 | up |
| 21 | ARL10 | 1.033654551 | 0.136717159 | down | 21 | HSPB8 | 2.17934725 | 0.178080442 | up |
| 22 | LTB | 3.075658064 | 0.174389439 | down | 22 | PDZK1 | 0.53121832 | 0.181130943 | up |
| 23 | CEP19 | 2.131376199 | 0.17778503 | down | 23 | BTN3A3 | 3.68580911 | 0.19252604 | up |
| 24 | PCDH18 | 2.267314382 | 0.178177725 | down | 24 | TMEM140 | 4.36465161 | 0.202654165 | up |
| 25 | SERPIND1 | 0.201799778 | 0.180783279 | down | 25 | WNT9A | 1.63113565 | 0.204722434 | up |
| 26 | FCGBP | 0.900381419 | 0.193707956 | down | 26 | PLEKHG1 | 1.89468832 | 0.205019055 | up |
| 27 | FLI1 | 2.904251024 | 0.194343427 | down | 27 | TJP3 | 2.85391734 | 0.20648233 | up |
| 28 | PGAM4 | 0.121289884 | 0.197784007 | down | 28 | ABAT | 3.93789313 | 0.217290812 | up |
| 29 | HAS3 | 0.818116566 | 0.205832448 | down | 29 | ESPN | 0.80036631 | 0.218111801 | up |
| 30 | TCN1 | 0.247082672 | 0.232502725 | down | 30 | ARSK | 2.37416421 | 0.251689696 | up |
| 31 | FAM83D | 3.794781688 | 0.234296468 | down | 31 | TMEM269 | 0.4352497 | 0.263475087 | up |
| 32 | PHOSPHO2 | 3.173598345 | 0.241486541 | down | 32 | FRY | 2.04541964 | 0.305752872 | up |
| 33 | CNTNAP3B | 0.102641155 | 0.245002289 | down | 33 | S100A6 | 6.72692449 | 0.308036388 | up |
| 34 | MMP9 | 3.948696519 | 0.245595145 | down | 34 | PROS1 | 3.46669947 | 0.312960252 | up |
| 35 | SERPINB4 | 0 | 0.246799122 | down | 35 | SGK3 | 3.10809307 | 0.323150656 | up |
| 36 | SERPINB3 | 0.042260991 | 0.258308605 | down | 36 | IL1RN | 1.44931777 | 0.330840076 | up |
| 37 | ANKRD36C | 0.916369005 | 0.259848903 | down | 37 | ADIRF | 5.98054431 | 0.346789757 | up |
| 38 | FAM186B | 0.478278169 | 0.275338443 | down | 38 | TRIM31 | 0.2070607 | 0.362069006 | up |
| 39 | ABCB4 | 0.626635065 | 0.276793698 | down | 39 | SERPINF2 | 1.75376068 | 0.373101741 | up |
| 40 | IL21R | 1.296494313 | 0.283848928 | down | 40 | RMDN2 | 1.54709838 | 0.384847414 | up |
| 41 | FOXP3 | 1.223897025 | 0.288150783 | down | 41 | WHRN | 3.42929502 | 0.397862331 | up |
| 42 | SLC26A9 | 0.050354501 | 0.304152104 | down | 42 | APOL2 | 4.81395131 | 0.400270214 | up |
| 43 | IGSF23 | 0.303500153 | 0.338159787 | down | 43 | ANXA8 | 0.04818453 | 0.40655423 | up |
| 44 | FOXI1 | 0 | 0.345869897 | down | 44 | PDGFA | 5.27572921 | 0.422822839 | up |
| 45 | LY6K | 0.459785601 | 0.363470322 | down | 45 | FXYD3 | 6.43765101 | 0.438317155 | up |
| 46 | TENM4 | 0.799087272 | 0.365026912 | down | 46 | CHPF2 | 5.11079026 | 0.442590288 | up |
| 47 | CSF3 | 0.036755817 | 0.368473594 | down | 47 | PLEKHA7 | 3.06657085 | 0.450530927 | up |
| 48 | HPSE2 | 0.126175465 | 0.37833981 | down | 48 | SAT1 | 8.88645493 | 0.458494064 | up |
| 49 | GBX2 | 0.232290344 | 0.384480875 | down | 49 | IDO1 | 1.28242844 | 0.471577403 | up |
| 50 | ABCA1 | 2.815936811 | 0.389474529 | down | 50 | HID1 | 6.65347734 | 0.488441853 | up |
| 51 | ZNF154 | 1.06081892 | 0.397670966 | down | 51 | TBX2 | 3.57826851 | 0.516634771 | up |
| 52 | CELF3 | 0.067958828 | 0.399795884 | down | 52 | SRPX | 3.01874973 | 0.520250383 | up |
| 53 | HSD3B1 | 0.037644885 | 0.414760927 | down | 53 | ARX | 0.82117587 | 0.520342577 | up |
| 54 | RLN1 | 0.713157666 | 0.460871825 | down | 54 | IL32 | 4.44159158 | 0.535950976 | up |
| 55 | ZNF596 | 1.908763433 | 0.482987341 | down | 55 | WNT4 | 1.7505174 | 0.538586429 | up |
| 56 | PRR29 | 1.057372573 | 0.484848132 | down | 56 | RORC | 4.77803231 | 0.543367691 | up |
| 57 | PADI3 | 0 | 0.487224549 | down | 57 | AKR1B1 | 4.71765848 | 0.560508284 | up |
| 58 | GPR19 | 0.837343267 | 0.488765968 | down | 58 | MFSD2A | 1.41763497 | 0.569923321 | up |
| 59 | TSPAN18 | 3.03297193 | 0.497803546 | down | 59 | GALNT12 | 2.15133749 | 0.576493172 | up |
| 60 | SCG2 | 1.111017825 | 0.504008849 | down | 60 | CFB | 3.12655052 | 0.581298598 | up |
| 61 | SERPINA3 | 3.124116101 | 0.505415051 | down | 61 | ANKRD37 | 4.98217304 | 0.607374633 | up |
| 62 | NRK | 0.052145238 | 0.518340008 | down | 62 | ATP2A3 | 4.23297218 | 0.611601554 | up |
| 63 | C5orf63 | 1.664698387 | 0.521817782 | down | 63 | BICDL1 | 3.26745179 | 0.614908108 | up |
| 64 | C11orf65 | 0.350710849 | 0.527739209 | down | 64 | IL36RN | 0.26693419 | 0.618561395 | up |
| 65 | LILRB3 | 1.328351242 | 0.539497514 | down | 65 | FIBCD1 | 0.48322124 | 0.618702318 | up |
| 66 | DMC1 | 0.162132012 | 0.547549573 | down | 66 | NLRC5 | 2.62371142 | 0.642107221 | up |
| 67 | ZFPM2 | 0.887710342 | 0.562158459 | down | 67 | PITPNM3 | 1.70050962 | 0.645319376 | up |
| 68 | CD14 | 4.994436225 | 0.563981769 | down | 68 | PKIA | 3.44423381 | 0.649471245 | up |
| 69 | SGK1 | 3.446035011 | 0.568336662 | down | 69 | SLC1A1 | 2.67921024 | 0.660307182 | up |
| 70 | PFKFB1 | 0.829818879 | 0.581449196 | down | 70 | PTPRR | 0.62962473 | 0.661512741 | up |
| 71 | PEG10 | 1.312621668 | 0.588746272 | down | 71 | FUT3 | 0.29947385 | 0.681542182 | up |
| 72 | IGFBP4 | 6.839243447 | 0.594911914 | down | 72 | KRT17 | 1.09491526 | 0.685923716 | up |
| 73 | NTF4 | 0.194736577 | 0.606529747 | down | 73 | NECTIN4 | 3.9264868 | 0.686878949 | up |
| 74 | MNX1 | 1.555752432 | 0.617960234 | down | 74 | PDGFRL | 2.17497098 | 0.686888556 | up |
| 75 | ABCB11 | 0.036492707 | 0.624465114 | down | 75 | CAPN12 | 0.69638122 | 0.702270348 | up |
| 76 | FLT1 | 3.153270239 | 0.625392118 | down | 76 | SLC44A4 | 7.83950827 | 0.705472236 | up |
| 77 | FAM43A | 4.558449082 | 0.63986105 | down | 77 | KIAA0319 | 0.52320956 | 0.708947886 | up |
| 78 | PHACTR3 | 0.216936959 | 0.651492125 | down | 78 | SDCBP2 | 1.69915319 | 0.71638859 | up |
| 79 | ZNF28 | 3.381056367 | 0.657650063 | down | 79 | SYN3 | 0.17495687 | 0.723945464 | up |
| 80 | KLK9 | 0 | 0.672905015 | down | 80 | FA2H | 1.92818643 | 0.740497953 | up |
| 81 | SLC45A2 | 0.68667932 | 0.689291905 | down | 81 | KLHDC7B | 0.35546911 | 0.742819554 | up |
| 82 | RPP25 | 2.958255968 | 0.689557977 | down | 82 | TRIM29 | 0.24071147 | 0.753638346 | up |
| 83 | EFCAB6 | 0.801259813 | 0.705422611 | down | 83 | TNFSF14 | 0.45890221 | 0.767878279 | up |
| 84 | APLN | 3.807444451 | 0.706495973 | down | 84 | PRKAR1B | 4.20653542 | 0.807946748 | up |
| 85 | SLITRK5 | 0.095046811 | 0.718153691 | down | 85 | FAM178B | 0.17396441 | 0.822207677 | up |
| 86 | SLC22A4 | 1.746325061 | 0.734951625 | down | 86 | SERINC5 | 6.5089876 | 0.823864649 | up |
| 87 | SVEP1 | 1.600987125 | 0.73957036 | down | 87 | CCNA1 | 0.07042432 | 0.832619669 | up |
| 88 | TTC30A | 2.302448155 | 0.745840035 | down | 88 | UGT2B7 | 0.02997736 | 0.841066322 | up |
| 89 | ANKRD63 | 0.095596526 | 0.752100611 | down | 89 | FER1L6 | 0.05269888 | 0.844666406 | up |
| 90 | BDKRB2 | 1.077102314 | 0.757974486 | down | 90 | TMOD1 | 1.49469995 | 0.852598027 | up |
| 91 | SAXO2 | 1.311701759 | 0.760783005 | down | 91 | GDPD3 | 2.0778604 | 0.867143655 | up |
| 92 | ASRGL1 | 5.381249919 | 0.768305857 | down | 92 | MVP | 5.32144649 | 0.869241114 | up |
| 93 | LGALS12 | 0.143204987 | 0.771297787 | down | 93 | STARD4 | 1.8104942 | 0.87375068 | up |
| 94 | MAP7D3 | 1.796046398 | 0.779852635 | down | 94 | STK32A | 0.43482113 | 0.881364708 | up |
| 95 | ADORA1 | 0.383222781 | 0.788381279 | down | 95 | UST | 1.68516569 | 0.885582166 | up |
| 96 | TNF | 0.93532081 | 0.789957473 | down | 96 | CGB5 | 0 | 0.891554136 | up |
| 97 | XKRX | 0.340040823 | 0.797072454 | down | 97 | LRRK1 | 2.64744604 | 0.892962841 | up |
| 98 | FANCI | 3.408912269 | 0.809493269 | down | 98 | AKR1C3 | 3.33774575 | 0.894627628 | up |
| 99 | NKD1 | 1.042299821 | 0.816925944 | down | 99 | NAV1 | 2.67252221 | 0.899307242 | up |
| 100 | ASB2 | 0.684547298 | 0.822014547 | down | 100 | CSF1R | 3.91337434 | 0.899323816 | up |
| 101 | INHBA | 2.672900657 | 0.826991342 | down | 101 | IGDCC4 | 0.55408325 | 0.908036974 | up |
| 102 | AADACL3 | 0 | 0.862739164 | down | 102 | CYP1B1 | 4.49011687 | 0.908092725 | up |
| 103 | DRP2 | 0.152820471 | 0.865167073 | down | 103 | GCNT3 | 0.12416523 | 0.9128846 | up |
| 104 | ANXA8L1 | 0.037862331 | 0.875801732 | down | 104 | SLFN11 | 2.59862177 | 0.91353782 | up |
| 105 | SHISA3 | 1.38997767 | 0.87682548 | down | 105 | ANG | 4.11772235 | 0.932960695 | up |
| 106 | MYH7B | 0.693631938 | 0.882077479 | down | 106 | NT5E | 2.42709205 | 0.9419372 | up |
| 107 | SERPINA5 | 0.313362169 | 0.889822739 | down | 107 | KDELR2 | 8.03900943 | 0.946008139 | up |
| 108 | PRKDC | 5.269338148 | 0.908434626 | down | 108 | ARG2 | 4.68463461 | 0.97062312 | up |
| 109 | BRINP2 | 0.05030519 | 0.936688981 | down | 109 | METRNL | 3.81407428 | 0.986083231 | up |
| 110 | RBL1 | 2.379541281 | 0.942142024 | down | 110 | AKR1C1 | 1.2358963 | 0.987849011 | up |
| 111 | DEPTOR | 4.131003294 | 0.944768239 | down | 111 | IFITM10 | 1.46436884 | 0.989829963 | up |
| 112 | MROH8 | 0.801807635 | 0.953250039 | down |  |  |  |  |  |
| 113 | PRR16 | 3.955374788 | 0.955393547 | down |  |  |  |  |  |
| 114 | FANCL | 3.962231168 | 0.964468972 | down |  |  |  |  |  |
| 115 | TNFRSF6B | 2.885869837 | 0.965140362 | down |  |  |  |  |  |
| 116 | LONRF2 | 2.554570087 | 0.965285081 | down |  |  |  |  |  |
| 117 | CAPSL | 0.452991416 | 0.975158297 | down |  |  |  |  |  |
| 118 | TTC30B | 2.335939581 | 0.982068374 | down |  |  |  |  |  |
| 119 | CXCL5 | 0.247211838 | 0.999274295 | down |  |  |  |  |  |
