# Supplementary figures and images for: Nitazoxanide inhibits acetylated KLF5-induced bone metastasis by modulating KLF5 function in prostate cancer
Source: BMC Med. 2023 Feb 21;21:68. doi: 10.1186/s12916-023-02763-4 (PMC9945734; doi:10.1186/s12916-023-02763-4)

**Ⅲ. Additional file 3**


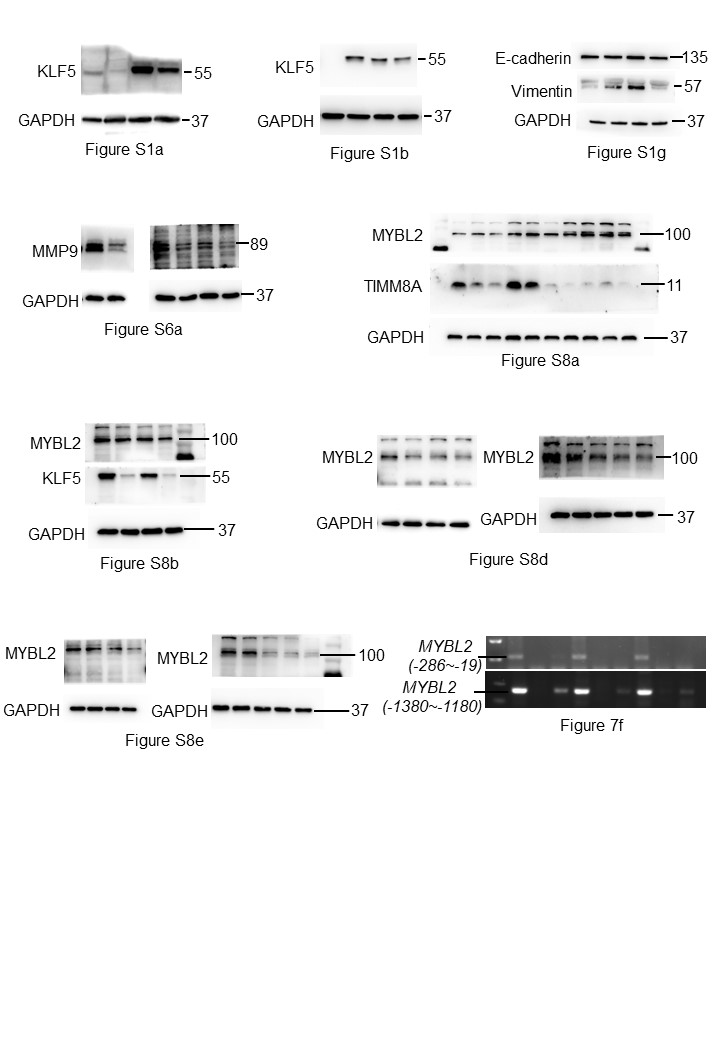


The uncropped immunoblot or gels images.

Supplement: Supplementary file 3 — Additional file 3. The uncropped immunoblot or gels images. [file 12916_2023_2763_MOESM3_ESM.docx]
